# Nine New and Five Known Polyketides Derived from a Deep Sea-Sourced *Aspergillus* sp. 16-02-1

**DOI:** 10.3390/md12063116

**Published:** 2014-05-27

**Authors:** Xiu-Wen Chen, Chang-Wei Li, Cheng-Bin Cui, Wei Hua, Tian-Jiao Zhu, Qian-Qun Gu

**Affiliations:** 1 Beijing Institute of Pharmacology and Toxicology, Beijing 100850, China; E-Mails: chenxiuwen2010@126.com (X.-W.C.); sdrlcw@126.com (C.-W.L.); huawei0917@outlook.com (W.H.); 2 College of Pharmacy, Guangxi Medical University, Nanning 530021, China; 3 Department of Biology and Chemistry, Hunan University of Science and Engineering, Yongzhou 425100, China; 4 Key Laboratory of Marine Drugs, Chinese Ministry of Education, Institute of Marine Drugs and Food, School of Medicine and Pharmacy, Ocean University of China, Qingdao 266003, China; E-Mails: zhutj@ouc.edu.cn (T.-J.Z.); guqianq@ouc.edu.cn (Q.-Q.G.)

**Keywords:** *Aspergillus* sp. 16-02-1, fungal strain from deep sea sediment, aspiketolactonol, aspilactonol, aspyronol, lactone, epiaspinonediol, polyketide, structure, cytotoxicity

## Abstract

Nine new C_9_ polyketides, named aspiketolactonol (**1**), aspilactonols A–F (**2**–**7**), aspyronol (**9**) and epiaspinonediol (**11**), were isolated together with five known polyketides, (*S*)-2-(2′-hydroxyethyl)-4-methyl-γ-butyrolactone (**8**), dihydroaspyrone (**10**), aspinotriol A (**12**), aspinotriol B (**13**) and chaetoquadrin F (**14**), from the secondary metabolites of an *Aspergillus* sp. 16-02-1 that was isolated from a deep-sea sediment sample. Structures of the new compounds, including their absolute configurations, were determined by spectroscopic methods, especially the 2D NMR, circular dichroism (CD), Mo_2_-induced CD and Mosher’s ^1^H NMR analyses. Compound **8** was isolated from natural sources for the first time, and the possible biosynthetic pathways for **1**–**14** were also proposed and discussed. Compounds **1**–**14** inhibited human cancer cell lines, K562, HL-60, HeLa and BGC-823, to varying extents.

## 1. Introduction

In recent five years from 2008 to 2012, more than 5500 new compounds have been discovered from marine natural products [1,2,3,4,5]. Marine microorganisms, especially the marine fungi, are increasingly a major focus of marine natural product researches, providing a lot of structurally novel and bioactive compounds [1,2,3,4,5,6,7,8,9,10]. Fungi from marine environments have great potential to be a rich source of drug leads [6,7,8,9,10] and over 30 compounds derived from marine microbes are currently in preclinical studies or clinical trials [10,11]. Recently, microorganisms from deep-sea habitats, including the hydrothermal vents, have become an interesting and newly emerging source of novel bioactive compounds [3,8,9,12]. Although a handful of reports described new compounds from fungi derived from these habitats [9], relevant researches have attracted considerable, growing research interest [13,14,15,16].

**Figure 1:**
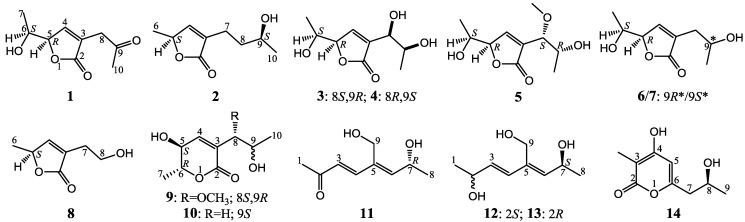
Structures of **1**–**14**.

During the ongoing search for new bioactive natural products from marine-sourced fungi, we have evaluated cytotoxicity and antifungal activities for 16 fungal strains from deep-sea habitats, and found that an *Aspergillus* sp. 16-02-1 produced metabolites with both cytotoxic and antifungal activities. The strain *Aspergillus* sp. 16-02-1 was isolated from a deep-sea sediment sample that was collected at a Lau Basin hydrothermal vent (depth 2255 m, temperature 114 °C) in southwest Pacific. We previously reported 8 known metabolites from this strain by a liquid fermentation [17]. In a continuation, we re-fermented this strain using solid-substrate fermentation medium and obtained nine new (**1**–**7**, **9** and **11**) and five known (**8**, **10**, and **12**–**14**) polyketides shown in Figure 1. We report herein the isolation, structure elucidation, and cytotoxicity evaluation of these compounds in detail.

## 2. Results and Discussion

### 2.1. Fermentation, Isolation, and Identification of Known Compounds

The fermentation and extraction of *Aspergillus* sp. 16-02-1 gave an ethyl acetate (EtOAc) extract that inhibited K562 cells with an inhibition rate (IR%) of 76.8% at 100 μg/mL. The repeated column chromatography of the EtOAc extract coupled with HPLC purification afforded 14 metabolites **1**–**14** (Figure 1). Among them, structures of the nine new compounds **1**–**7**, **9** and **11** were elucidated by modern spectroscopic methods, and named aspiketolactonol (**1**), aspilactonols A–F (**2**–**7**), aspyronol (**9**) and epiaspinonediol (**11**). The five known compounds **8**, **10**, and **12**–**14** were identified as (*S*)-2-(2′-hydroxyethyl)-4-methyl-γ-butyrolactone (**8**) [18], dihydroaspyrone (**10**) [19], aspinotriol A (**12**) [19], aspinotriol B (**13**) [19] and chaetoquadrin F (**14**) [20] by the physicochemical and spectroscopic data.

### 2.2. Structure Determination of New Compounds

Aspiketolactonol (**1**), colorless oil (from MeOH), 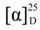 +10.5 (*c* 0.12, MeOH), was assigned the molecular formula C_9_H_12_O_4_ by HRESIMS (measured 185.0811 [M + H]^+^, calculated for C_9_H_13_O_4_ [M + H]^+^ 185.0814). It showed end UV absorption, and the IR spectrum showed the absorptions due to OH (3417 cm^−1^), CH_3_/CH_2_ (2980, 2938, 2906, 1422 and 1360 cm^−1^), α,β-unsaturated γ-lactone (1752 and 1656 cm^−1^) [21,22] and keto carbonyl (1722 cm^−1^) groups. The olefinic proton and carbon signals at the lower field (δ_H_ 7.43 and δ_C_ 149.4) [22] of ^1^H and ^13^C NMR spectra (Table 1 and Table 2) and the ester carbonyl carbon signal at δ_C_ 173.5 in the ^13^C NMR spectrum (Table 2) supported the presence of the α,β-unsaturated γ-lactone moiety. The carbonyl carbon signal at δ_C_ 203.6 in the ^13^C NMR spectrum further supported the presence of one keto carbonyl in **1**. Interpretation of the ^1^H–^1^H COSY, HMQC and HMBC data (Table S1 in the Supplementary Information) established the planar structure. The structural part related to the ^1^H spin system, C-7–C-4(via quaternary sp^2^ C-3)–C-8, was deduced from the ^1^H–^1^H COSY and HMQC data, including the allylic coupling between H-4/H_2_-8, which was confirmed by the HMBC correlations of H-4, H-5 and H_2_-8 with C-3. The acetyl group that consisted of the C-9 keto carbonyl and the C-10 methyl was linked to C-8 by the HMBCs on H_2_-8/C-9, H_3_-10/C-8 and H_3_-10/C-9. The ester carbonyl carbon (C-2, δ_C_ 173.5) was linked to C-3 by the HMBC correlations of H-4 and H_2_-8 with C-2. Then, C-2 was further linked to C-5 by an ester linkage to form the α,β-unsaturated γ-lactone ring according to the IR absorption at 1752 cm^−1^.

**Figure 2:**
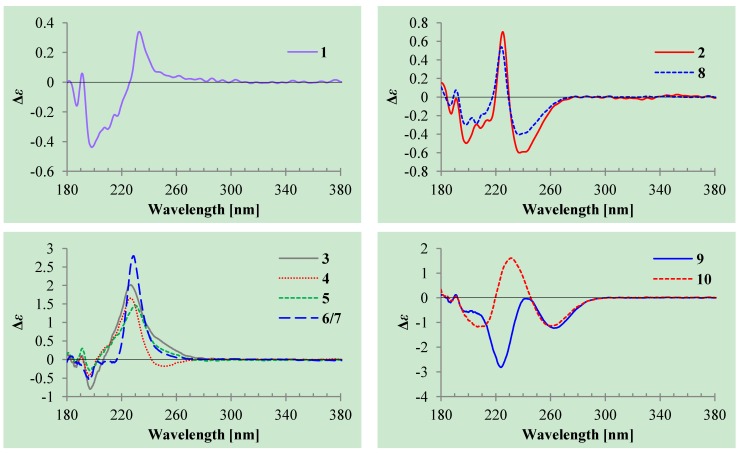
Circular dichroism (CD) spectra of compounds **1**–**10** in MeOH.

The stereochemistry of **1** was determined as follows. The coupling of H-5/H-6 (4.7 Hz) indicated the *erythro* relative configuration of 5,6-diol in **1**. The couplings of the same protons are larger than 4 Hz in *erythro* isomers but smaller than 2 Hz in *threo* isomers in the α,β-unsaturated γ-lactones [23,24,25]. The CD of the α,β-unsaturated γ-lactone rings with a chiral γ-carbon shows Cotton effects associated with the π→π* transition in the region 200–235 nm [26] and the n→π* transition in the region 235–270 nm [26,27,28,29]. Generally the π→π* Cotton effect is decisive to assign the absolute configuration of the α,β-unsaturated γ-lactone ring because of the easy influence of the n→π* Cotton effect by external asymmetry [26,27]. Compound **1** showed positive π→π* Cotton effect at 233 nm (Figure 2), indicating the *R* absolute configuration at C-5 [26,27]. The absolute configuration at C-6 was thus assigned to be *S* according to the *erythro* relative configuration of 5,6-diol in **1**.

Aspilactonol A (**2**), colorless oil (MeOH), 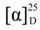 +24.6 (*c* 0.23, MeOH), was assigned the molecular composition C_9_H_14_O_3_ by HRESIMS (measured 171.1015 [M + H]^+^, calculated for C_9_H_15_O_3_ [M + H]^+^ 171.1021). The IR spectrum showed absorptions ascribable to the OH, CH_3_/CH_2_ and α,β-unsaturated γ-lactone groups (Experimental Section). Similar to **1**, the olefinic proton and carbon signals at the lower field (δ_H_ 7.04, H-4 and δ_C_ 149.8, C-4) [22] of the ^1^H and ^13^C NMR spectra (Table 1 and Table 2) together with the ester carbonyl (δ_C_ 174.3 C-2) and the sp^2^ carbon (δ_C_ 134.0, C-3) signals in the ^13^C NMR spectrum (Table 2) supported the presence of the α,β-unsaturated γ-lactone moiety in **2**. Then, the planar structure was established by interpretation of the ^1^H–^1^H COSY, HMQC and HMBC data (Table S2 in the Supplementary Information). The carbon backbone chain related to the ^1^H spin system, C-6–C-4(via quaternary sp^2^ C-3)–C-7–C-10, could be derived from the ^1^H–^1^H COSY and HMQC data (Table S2 in the Supplementary Information), including the allylic coupling between H-4/H_2_-7, which was confirmed by the HMBC correlations of H-4, H-5, H_2_-7 and H_2_-8 with C-3. The carbonyl carbon C-2 (δ_C_ 174.3) was linked to C-5 and C-3 by the HMBC correlations between H-5/C-2, H-4/C-2 and H_2_-7/C-2 to form the α,β-unsaturated γ-lactone ring with the ester linkage between C-5 and C-2.

**Table 1:** 400 MHz ^1^H NMR data of compounds **1**–**7** (δ_H_, *J* in Hz) ^a^.

Proton	1	2	3	4	5	6/7 ^b^
4	7.43 q (1.4)	7.04 q (1.6)	7.54 dd (3.0, 1.5)	7.56 dd (2.8, 1.6)	7.55 dd (1.5, 0.9)	7.41/7.32, dd (2.8, 1.6)
5	4.88 dq (4.7, 1.4)	5.01 qq (6.9,1.6)	4.96–4.92 m	4.92 ddd (5.0, 2.8, 1.6)	4.98 dt (4.5, 1.5)	4.87 dd (4.4, 2.8, 1.6)
6	4.02 qd (6.5, 4.7)	1.41 3H d (6.9)	3.95 qd (6.5, 4.6)	3.92 qd (6.4, 5.0)	3.97 qd (6.4, 4.5)	3.92 qd (6.4, 4.4)
7	1.28 3H d (6.5)	2.28–2.57 2H m	1.25 3H d (6.5)	1.26 3H d (6.4)	1.26 3H d (6.4)	1.25 3H d (6.4)
8	3.46 2H t (1.4)	1.60–1.78 2H m	4.36 dt (4.6, 1.5)	4.34 br d (4.9)	3.96 br d (4.7)	2.41 2H br d (6.2)
9	-	3.80 sext (6.4)	4.03 qd (6.4, 4.6)	3.99 qd (6.4, 4.9)	4.04 qd (6.4, 4.7)	4.02 sext (6.2)
10	2.24 3H s	1.21 3H d (6.4)	1.12 3H d (6.4)	1.12 3H d (6.4)	1.12 3H d (6.4 )	1.19 3H d (6.2)
OCH_3_	-	-	-	-	3.35 3H s	-

^a^ Data were taken in CDCl_3_ for **1**–**2** and in CD_3_OD for **3**–**7**, and the δ_H_ values were recorded using solvent signals (CDCl_3_: δ_H_ 7.26 for **1**–**2**; CD_3_OD: δ_H_ 3.31 for **3**–**7**) as references. Signal assignments were based on the results of ^1^H–^1^H COSY, HMQC and HMBC experiments. ^b^ A pair of H-4 signals in an approximate 1:1 ratio corresponded to the 1:1 mixture of **6**/**7**, the isomers at C-9.

**Table 2:** 100 MHz ^1^^3^C NMR data of compounds **1**–**7** (δ_C_, multiplicity) ^a^.

Position	1	2	3	4	5	6/7 ^b^
2	173.5 s	174.3 s	174.5 s	174.5 s	174.6 s	176.25 s/176.18 s
3	128.2 s	134.0 s	136.9 s	137.0 s	133.9 s	133.08 s/133.04 s
4	149.4 d	149.8 d	150.3 d	150.5 d	152.0 d	150.49 d/150.03 d
5	85.5 d	77.8 d	87.1 d	87.0 d	87.4 d	86.85 d/86.82 d
6	67.8 d	19.3 q	68.5 d	68.6 d	68.5 d	68.57 d/68.33 d
7	18.9 q	21.6 t	19.0 q	19.1 q	19.0 q	19.15 q/19.02 q
8	39.1 t	37.2 t	71.7 d	71.7 d	81.6 d	35.72 t/35.67 t
9	203.6 s	67.0 d	69.8 d	70.1 d	69.0 d	66.49 d/66.47 d
10	30.3 q	23.6 q	17.7 q	17.9 q	18.2 q	23.26 q
OCH_3_	-	-	-	-	58.1 q	-

^a^ Data were taken in CDCl_3_ for **1**–**2** and in CD_3_OD for **3**–**7**, and the δ_C_ values were recorded using solvent signals (CDCl_3_: δ_C_ 77.16 for **1**–**2**; CD_3_OD: δ_C_ 49.00 for **3**–**7**) as references. Signal assignments were based on the results of ^1^H–^1^H COSY, HMQC and HMBC experiments. ^b^ Pairs of C-2–C-9 signals in an approximate 1:1 ratio corresponded to the 1:1 mixture of **6**/**7**, the isomers at C-9.

**Figure 3:**
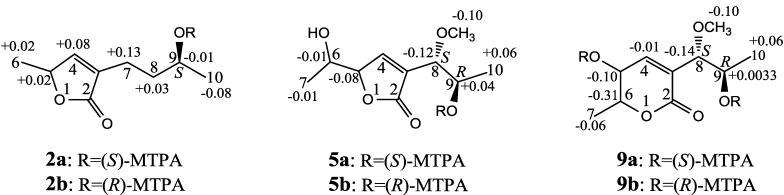
The Δδ (δ_S_–δ_R_) values from the (*S*)- and (*R*)-MTPA esters of **2**, **5** and **9**.

The absolute configuration at C-5 was assigned to be *S* by the positive π→π* Cotton effect at 225 nm and the negative n→π* Cotton effect around 239 nm (Figure 2) [26,27]. The absolute configuration at C-9 was assigned by the modified Mosher’s method [30,31]. Treatment of **2** with (*S*)-α-methoxy-α-trifluoromethyl phenylacetyl chloride [(*S*)-MTPA-Cl] and (*R*)-MTPA-Cl gave (*S*)-MTPA ester (**2a**) and (*R*)-MTPA ester (**2b**), respectively. The Δδ (δ_S_–δ_R_) values of relevant proton signals from **2a** and **2b** established the *S* absolute configuration of C-9 in **2** (Figure 3).

Aspilactonol B (**3**), 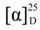 +27.2 (*c* 0.32, MeOH), and aspilactonol C (**4**), 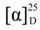 +34.5 (*c* 0.41, MeOH), colorless oils (MeOH), were assigned the molecular composition C_9_H_14_O_5_ by HRESIMS (measured 203.0919 [M + H]^+^ for **3** and 203.0910 [M + H]^+^ for **4**, calculated for C_9_H_15_O_4_ [M + H]^+^ 203.0919). Both they showed end UV absorptions, and similar to **1** and **2**, their IR spectra revealed the presence of α,β-unsaturated γ-lactone moieties (around 1740 and 1652 cm^−1^) [21,22]. This was supported by the lower field olefinic proton and carbon signals (around δ_H_ 7.55, H-4; around δ_C_ 150.4, C-4) [22] in the ^1^H and ^13^C NMR spectra (Table 1 and Table 2) and the ester carbonyl (δ_C_ 174.5, C-2) and the sp^2^ carbon (around δ_C_ 137, C-3) signals in the ^13^C NMR spectra (Table 2). Their IR spectra also indicated the presence of OH and CH_3_/CH_2_ groups, and the strong OH signals around 3384 cm^−1^ further revealed more OH groups in **3** and **4** than in **1** and **2** (the IR spectra in the Supplementary Information). Interpretation of the ^1^H–^1^H COSY and HMQC data (Tables S3 and S4 in the Supplementary Information) established the carbon backbone chain related to the proton spin system, C-7–C-4(via quaternary sp^2^ C-3)–C-8–C-10. The allylic couplings between H-4 and H-8 in **3** and **4** indicated the connection of C-4 and C-8 via the quaternary sp^2^ carbon C-3, and the C-3 carbon was assigned by the HMBC correlations of H-4, H-5, H-8 and H-9 with C-3 (Tables S3 and S4 in the Supplementary Information). The C-2 carbonyl carbons in **3** and **4** were linked to C-5 and C-3 by the HMBC correlations between H-5/C-2, H-4/C-2 and H-8/C-2 to form the α,β-unsaturated γ-lactone rings with the ester linkage between C-5 and C-2.

**Figure 4:**
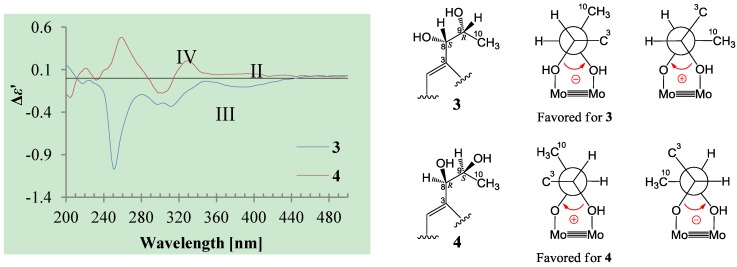
Induced CD (ICD) spectra from the Mo_2_-complexes of **3** and **4** in DMSO.

The absolute configuration at C-5 in **3** and **4** was assigned both to be *R* by the positive π→π* Cotton effect around 226 nm in the CD of **3** and **4** (Figure 2) [26,27]. Because the coupling of H-5 and H-6 (4.6 Hz for **3** and 5.0 Hz for **4**) indicated the *erythro* relative configuration of 5,6-diols in **3** and **4** [23,24,25], the absolute configuration at C-6 in **3** and **4** was also assigned both to be *S*. Thus, **3** and **4** should be stereoisomers at the vicinal diol methine carbons C-8/C-9. There are many reports recorded that the coupling of vicinal diol methine protons is generally lager than 6 Hz in *threo* isomers but smaller than 5 Hz in *erythro* isomers of the relevant compounds with a vicinal diol unit similar to the 8,9-diols in **3** and **4** [32,33,34,35,36]. The coupling of H-8 and H-9 (4.6 Hz for **3** and 4.9 Hz for **4**) indicated the *erythro* relative configuration of the 8,9-diols in **3** and **4** [32,33,34,35,36]. The absolute configuration of the *erythro*-8,9-diols in **3** and **4** was determined by the dimolybdenum induced CD (ICD) analysis. In the ICDs by the Snatzke’s method using dimolybdenum tetraacetate (Mo_2_(OAc)_4_) in DMSO [37,38], the Mo_2_-complex of **3** gave negative CD bands II (near 400 nm) and IV (around 329 nm), while the Mo_2_-comlex of **4** gave the positive bands II and IV (Figure 4). By the Snatzke’s helicity rule, the sign of O–C–C–O torsional angle in the favored conformation of the chiral Mo_2_-complexes determines the sign of the bands II and IV [37,38]. We have demonstrated that in the *erythro*-diols closely resembled **3** and **4**, the conformation with an antiperiplanar orientation of the OH and methyl groups, O–C–C–CH_3_, is favored conformation of the Mo_2_-complexes [39], as shown for **3** and **4** in Figure 4. Therefore, the absolute configuration at C-8 and C-9 in **3** and **4** could be assigned to be 8*S*,9*R* for **3** and 8*R*,9*S* for **4** on the basis of their band II and IV signs (Figure 4), respectively.

Aspilactonol D (**5**), colorless oil (MeOH), 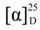 −1.7 (*c* 0.38, MeOH), was assigned the molecular formula C_10_H_16_O_5_ by HRESIMS (measured 217.1071 [M + H]^+^, calculated for C_10_H_17_O_5_ [M + H]^+^ 217.1076), which had a CH_2_ composition more than in **3** and **4**. It showed UV and IR absorptions similar to **3** and **4**, and the IR absorptions revealed the presence of OH, CH_3_/CH_2_ and α,β-unsaturated γ-lactone groups (Experimental Section). The ^1^H and ^13^C NMR spectra of **5** in CD_3_OD showed signals similar to **3** and **4** except additional signals ascribable to an *O*-methyl group were detected (Table 1 and Table 2). These NMR data indicated that **5** was an *O*-methylated. Analyses of the ^1^H–^1^H COSY, HMQC and HMBC spectra (Table S5 in the Supplementary Information) established its planar structure. The α,β-unsaturated γ-lactone ring was confirmed by the HMBC correlations between H-5/C-2, H-4/C-2 and H-8/C-2. The *O*-methyl group was located at C-8 by the HMBC correlations of the *O*-methyl protons with C-8. The absolute configuration at C-5 in **5** was assigned to be *R* by the positive π→π* Cotton effect at 231.5 nm in the CD spectrum (Figure 2) [26,27]. The coupling of H-5/H-6 (4.5 Hz) indicated the *erythro* relative configuration of 5,6-diol in **5** [23,24,25], and thus the absolute configuration at C-6 was assigned to be *S*. The *R* absolute configuration of C-9 was determined by the modified Mosher’s method [30,31] on the basis of the Δδ (δ_S_–δ_R_) values from the (*S*)- and (*R*)-MTRA esters (Figure 3). Since the coupling of H-8/H-9 (4.7 Hz) indicated the *erythro* relative configuration of 8,9-diol in **5** [32,33,34,35,36], the absolute configuration at C-8 was assigned to be *S*.

Aspilactonols E/F (**6**/**7**) were obtained as a mixture of epimers as a colorless oil from MeOH, 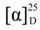 +78.1 (*c* 1.00, MeOH), and the molecular formula C_9_H_14_O_4_ was determined by HRESIMS (measured 187.0967 [M + H]^+^, calculated for C_9_H_15_O_5_ [M + H]^+^ 187.0970). In the UV spectrum, **6**/**7** showed the end absorption, and the IR spectrum showed absorptions due to the OH, CH_3_/CH_2_ and α,β-unsaturated γ-lactone groups (Experimental Section). The ^1^H and ^13^C NMR spectra of **6**/**7** in CD_3_OD showed signals similar to **3**–**5**, but they were characterized by the appearance of methylene signals instead of the signals from an oxygenated methine in **3**–**5** and an *O*-methyl group in **5** (Table 1 and Table 2). These NMR data indicated the same skeletal structures in **3**–**7**. The appearance of the proton H-4 and the carbon signals except for the C-10 signal as pairs in an approximate 1:1 ratio indicated that **6**/**7** was a 1:1 mixture of stereoisomers. The planar structure of **6**/**7** was deduced by the ^1^H–^1^H COSY, HMQC and HMBC data (Table S6 in the Supplementary Information), coupled with the IR absorptions at 1748 and 1651 cm^−1^ from an α,β-unsaturated γ-lactone ring [21,22]. The absolute configuration at C-5 in **6**/**7** was assigned both to be *R* by the strong positive π→π* Cotton effect at 228.5 nm in the CD spectrum (Figure 2) [26,27]. Since the coupling of H-5/H-6 (4.4 Hz) indicated the *erythro* relative configuration of 5,6-diols in **6**/**7** [23,24,25], the absolute configuration at C-6 was assigned to be *S* for both **6**/**7**. Thus, **6**/**7** was a 1:1 mixture of epimers at C-9. Although a Doctor’s Thesis has recorded the same planar structure of **6**/**7**, its stereochemistry was not elucidated [40]. We therefore named **6**/**7** as aspilactonols E/F as new compounds.

Aspyronol (**9**), colorless oil from MeOH, 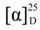 −41.6 (*c* 0.22, MeOH), was assigned the molecular formula C_10_H_16_O_5_ by HRESIMS (measured 217.1073 [M + H]^+^, calculated for C_10_H_17_O_5_ [M + H]^+^ 217.1076). It showed end UV absorption and the IR absorptions due to OH (3398 cm^−1^), CH_3_/CH_2_ (2982, 2938, 2905, 2835, 1451 and 1384 cm^−1^), C=O (1714 cm^−1^) and C=C (1649 cm^−1^) groups. The ^1^H and ^13^C NMR spectra (Table 3) resembled those of dihydroaspyrone (**10**) except additional signals from a methoxy and an oxygenated methine groups were detected instead of the methylene signals in **10**. There were also slight changes in several ^1^H and ^13^C signals. These NMR data suggested that **9** was a methoxylated derivative of **10**, and this was confirmed by analysis of the ^1^H–^1^H COSY, HMQC and HMBC spectra (Table S7 in the Supplementary Information) to complete the planar structure. The carbon chain related to the proton spin system, C-7–C-4(via quaternary sp^2^ C-3)–C-8–C-10, was deduced by interpretation of the ^1^H–^1^H COSY and HMQC data (Table S7 in the Supplementary Information). The allylic coupling between H-4 and H-8 suggested the connectivity of C-4 and C-8 via a quaternary sp^2^ carbon C-3, and C-3 was assigned by the HMBC correlations between H-8/C-3 and H-9/C-3. The OCH_3_ group was located at C-8 by the HMBCs of the methoxy protons with the carbon C-8. The carbonyl carbon C-2 was linked to C-3 by the HMBCs on H-4/C-2 and H-8/C-2. The ester linkage of the C-2 carbonyl was then linked to C-6 to form a δ-lactone ring by the coupling of H-5/H-6 (9.4 Hz), which requires C-5/C-6 fixed in a six-membered ring with *trans* orientated H-5/H-6. This was supported by the chemical shift of C-6, δ_C_ 80.0 in **9** and δ_C_ 79.6 in **10**. The *R* absolute configuration at C-9 was determined by the modified Mosher’s method [30,31] on the basis of the Δδ (δ_S_–δ_R_) values from the (*S*)- and (*R*)-MTRA esters of **9** (Figure 3), and the absolute configuration at C-8 was assigned to be *S* because the coupling of H-8/H-9 (4.6 Hz) indicated the *erythro* relative configuration of 8,9-diol in **9**[32,33,34,35,36]. The absolute configuration at C-5 and C-6 in **9** was determined to be 5*S*,6*R*, the same as **10**, according to the negative signs of the Cotton effects around 260 nm both from the chiral α,β-unsaturated δ-lactone units in **9** and **10** (Figure 2) [28]. This was also supported by the co-generation of **9** and **10** by the same *Aspergillus* sp. 16-02-1 strain from a biogenetic consideration.

**Table 3:** 400 MHz ^1^H and 100 MHz ^13^C NMR data of **9** ^a^.

NO.	in CD_3_OD		in CDCl_3_	
	δ_C_	δ_H_ (*J* in Hz)	δ_H_ (*J* in Hz)	NOE
2	165.7 s	-	-	-
3	129.9 s	-	-	-
4	147.8 d	6.78 t (1.4)	6.79 dd (2.6, 0.9)	
5	68.7 d	4.21 dd (9.4,1.4)	4.25−4.20 m (masked by H-8)	H-7
6	80.0 d	4.24 dq (9.4, 5.8)	4.35 dq (8.5, 6.4)	
7	18.20 q	1.44 3H d (5.8)	1.48 3H d (6.4)	H-5
8	83.5 d	4.08 dd (4.9, 1.4)	4.22 br d (4.6)	
9	70.1 d	3.82 qd (6.4,4.9)	3.94 qd (6.5,4.6)	
10	18.17 q	1.12 3H d (6.4)	1.10 3H d (6.5)	
OCH_3_	58.0 q	3.32 3H s	3.31 3H s	

^a^ Chemical shifts were recorded in δ values using the solvent signals (CDCl_3_: δ_H_ 7.26; CD_3_OD: δ_H_ 3.31/δ_C_ 49.00) as references, respectively. Signals were assigned on the basis of ^1^H–^1^H COSY, HMQC and HMBC experiments.

Epiaspinonediol (**11**), yellow oil (MeOH), 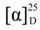 −5.4 (*c* 0.14, MeOH), was assigned the molecular formula C_9_H_14_O_3_ by HRESIMS (measured 193.0842 [M + Na]^+^, calculated for C_9_H_14_O_3_Na [M + Na]^+^ 193.0841). The ^1^H and ^13^C NMR data of **11** in CD_3_OD were identical with those of aspinonediol in CD_3_OD [19], indicating the same planar structures of both compounds. Aspinonediol had the absolute configuration 7*S* and showed 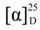 +2.4 (*c* 0.63, MeOH) [19]. In contrast, **11** showed the opposite optical rotation. Thus, the epiaspinonediol (**11**) was determined to be the epimer of aspinonediol at C-7 with the 7*R* absolute configuration.

### 2.3. Inhibitory Effects of **1***–***14** on Several Human Cancer Cell Lines

Antitumor activities of **1***–***14** were tested by the MTT method using the human cancer K562, HL-60, HeLa, and BGC-823 cell lines. Compounds **1***–***14** inhibited some of the tested four cell lines and the IR% values at 100 μg/mL are given below. **1**: 11.7% (HL-60); **2**: 17.7% (HL-60); **3**: 22.2% (HL-60); **4**: 20.0% (K562), 16.7% (HL-60); **5**: 20.0% (HL-60), 13.2% (BGC-823); **6**/**7**: 14.1% (K562), 13.4% (HeLa); **8**: 14.2% (HL-60), 15.7% (HeLa); **9**: 27.9% (K562), 67.2% (HL-60), 14.0% (HeLa); **10**: 19.9% (HL-60), 10.1% (HeLa); **11**: 79.7% (K562); 72.5% (HL-60), 14.9% (HeLa), 21.8 (BGC-823); **12**: 17.0% (K562); 14.1% (HeLa); **13**: 20.3% (K562), 39.4% (HL-60), 12.3% (HeLa), 15.7% (BGC-823); **14**: 13.5% (HeLa). The half-inhibitory concentration (IC_50_) of **9** on HL-60 cells was determined to be 52.1 μg/mL (241.2 μM), and the IC_50_ for **11** on K562 and HL-60 cells to be 44.3 μg/mL (260.6 μM) and 32.8 μg/mL (192.9 μM), respectively. The positive control docetaxol inhibited these cell lines with the IR% values of 55.6% (K562), 49.9% (HL-60), 45.1% (HeLa), and 61.5% (BGC-823) at 100 μg/mL.

### 2.4. Discussions

Chemical investigation of a deep sea-sourced *Aspergillus* sp. 16-02-1 has resulted in the elucidation of 14 secondary metabolites **1**–**14**, including nine new (**1**–**7**, **9** and **11**) and five known (**8**, **10**, and **12**–**14**) compounds, shown in Figure 1. Although compound **8** has been chemically prepared [18], it is the first time to report **8** from natural sources in present study. Structures of the new compounds, including their absolute configurations, were determined by extensive spectroscopic methods, especially the 2D NMR, CD, ICD and Mosher’s ^1^H NMR analyses. The determination of the absolute configuration of α,β-unsaturated γ-lactone ring in **1**–**7** mainly relied on the CD data. In most cases, a chiral α,β-unsaturated γ-lactone ring gave both π→π* and n→π* Cotton effects with the opposite sign [26,27] in 200–235 nm and 235–270 nm regions, respectively [26,27,28,29]. However, usually the n→π* Cotton effect is weak and sometimes could not be observed [26,27] or even appeared with the same sign of the π→π* Cotton effect [26]. The same is true of the case of **1**–**8**. As shown in Figure 2, **2** and **8** gave both opposite n→π* and π→π* Cotton effects in similar magnitude and **4** also showed a weak n→π* Cotton effect opposite to the π→π* transition. However, the others did not give opposite n→π* Cotton effect and rather they showed a weak CD curve with the same sign of the π→π* Cotton effect in the n→π* transition region (Figure 2). Since the easy influence of the n→π* Cotton effect by the external asymmetry [26] and the decisive role of the π→π* Cotton effect in absolute configuration assignment has been known [26,27], the absolute configurations of the α,β-unsaturated γ-lactone rings in **1**–**7** could be assigned by the sign of their π→π* Cotton effects shown in Figure 2.

**Scheme 1:**
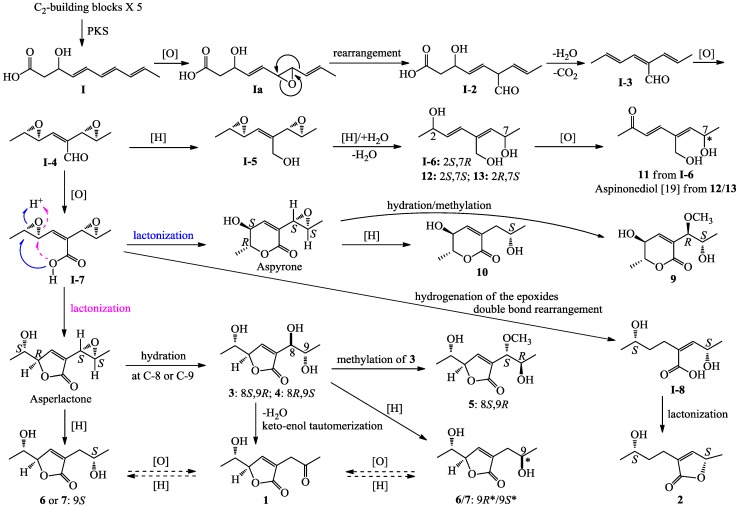
Plausible biosynthetic pathways of **1**–**7** and **9**–**13**.

Compounds **1**–**14** are all polyketides derived from a branched C_9_ (**1**–**7** and **9**–**14**) or C_7_ (**8**) carbon skeleton, and are derivatives of α,β-unsaturated γ-lactone (**1**–**8**), α,β-unsaturated δ-lactone (**9**–**10** and **14**) or branched, acyclic linear C_8_ carbon backbone chain with a conjugated diene group (**11**–**13**). The biosynthesis of aspyrone [41,42,43,44,45,46], asperlactone and isoasperlactone [44,45,46], and aspinonene [47], their structures were closely related to the compounds, **9**–**10**/**14**, **1**–**8**, and **11**–**13**, respectively, have been exhaustively studied [41,42,43,44,45,46,47], and as indicated in Scheme 1 [46,47], it has been demonstrated that these metabolites derive from a common biosynthetic precursor, **I-4**, which originates from the intermediate **I**, the ultimate product of polyketide synthesis (PKS), by post-PKS modifications (see in Scheme 1). Reduction of the aldehyde in **I-4** into the primary alcohol give **I-5**, which undergo further modification to produce aspinonene [47], while oxidation of the aldehyde in **I-4** into the carboxyl afford **I-7**, which switches the formation of aspyrone and asperlactone by nucleophilic attack of the carboxyl group on either site of the carbons in one of the two epoxide groups [44,45,46,47].

Relating to the above mentioned metabolites, plausible biosynthetic pathways for **1**–**7** and **9**–**13** are proposed in Scheme 1. Reduction of either one of the two epoxides in **I-5**, the precursor of aspinonene [47], coupled with hydration at either site of the other epoxide ring followed by dehydration, would give **I-6** and compounds **12** and **13**, which further underwent oxidation at C-2 to produce compound **11** and aspinonediol, the epimer of **11** at C-7 (Scheme 1). Aspyrone and asperlactone are proposed to be intermediates for **9**–**10** and **1**/**3**–**7**. Reduction of the epoxide in aspyrone would give **10**, and hydration at C-8 of the epoxide, followed by methylation, would afford **9** (Scheme 1). Reduction or hydration of the epoxide in asperlactone and further modification of the products by methylation, reduction or dehydration followed by keto-enol tautomerization, and oxidation/reduction reactions would produce **1** and **3**–**7**, as shown in Scheme 1. The γ-lactone **2** seems likely to be produced from the intermediate **I-7**. Reduction of the epoxides in **I-7** coupled with double bond rearrangement, followed by lactonization of the product **I-8**, would give **2** (Scheme 1).

In view of the structural features of **1**, **3**–**4** and **6**–**7**, one of these compounds may probably form from some or other during extraction with EtOAc at the slightly acidic conditions by the acid-catalyzed chemical reactions artificially. In order to confirm whether this occurred in truth, each 0.3 mg of the crude compound samples was dissolved in 0.2 mL water-saturated EtOAc in a 0.5 mL Eppendorf tube, capped the tube and kept at room temperature for 6 days, and then further treated at 50 °C for 16 h. These treatment conditions simulated the extraction conditions (whole extraction was achieved within 4 days with a total of 12 h evaporating times at the temperature lower than 40 °C). Then, the aqueous EtOAc was removed by blowing inside of the tube with nitrogen gas to dryness. The residue was dissolved in MeOH and then subjected to HPLC analysis. No any one of them was detected in other compound samples by the HPLC analysis (Figure S1 in the Supplementary Information), confirming that none of these compounds are artificial product formed from the others in the extraction conditions.

**Scheme 2:**
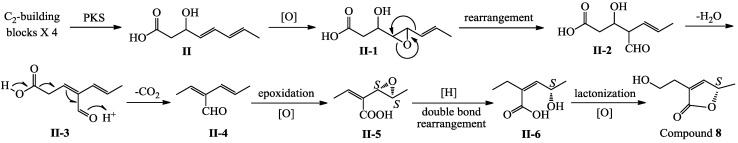
Plausible biosynthetic pathway of **8**.

Differing from **1**–**7**/**9**–**13**, they are all pentaketide derivatives as shown in Scheme 1, compound **8** seems likely to be a tetraketide derivative. A plausible pathway for the **8** biosynthesis is proposed in Scheme 2. A first intermediate in this pathway was proposed to be **II**, the ultimate tetraketide product of PKS, like the intermediate **I** in Scheme 1. Similar to the **1**–**7**/**9**–**13** biosynthesis from **I** as shown in Scheme 1, post-PKS modifications of **II** would give a key intermediate **II-4**, which underwent further modification would produce **8**.

**Scheme 3:**
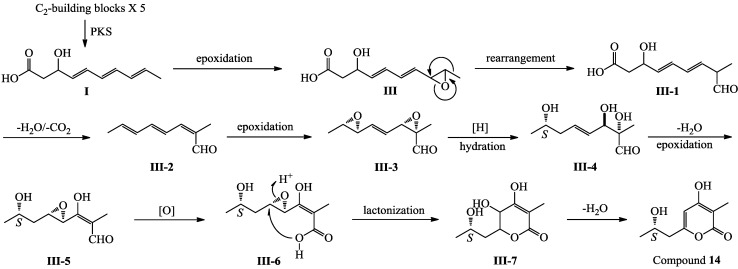
Plausible biosynthetic pathway of **14**.

Compound **14** is also a pentaketide derivative that seems likely to be derived from the intermediate **I** shown for **1**–**7** and **9**–**13** in Scheme 1. A plausible biosynthetic pathway for **14** is proposed in Scheme 3. Similar to the biosynthesis of **1**–**7**/**9**–**13** (Scheme 1), epoxidation of the double bond adjacent to the methyl group in **I**, followed by rearrangement, dehydration and decarboxylation reactions, would give a key intermediate **III-2**, which underwent further modification would produce **14** (Scheme 3).

In present MTT assay, **9** and **11** showed stronger inhibitory effect than the others on human cancer K562 and/or HL-60 cell lines. Compound **9** inhibited the HL-60 cells with the IR% value of 67.2% at 100 μg/mL, while **11** inhibited the K562 and HL-60 cells with the IR% values of 79.7% and 72.5% at 100 μg/mL, respectively. Both compounds also inhibited to some extents the K562 (**9**: an IR% of 27.9% at 100 μg/mL) and the BGC-823 (**11**: an IR% of 21.8% at 100 μg/mL) cells. These data suggested that the new compounds **9** and **11** showed somewhat selective inhibitory effect on the HL-60 cells and the K562 and HL-60 cells, respectively, although the inhibitory effect of both compounds by the IC_50_ of **9** (52.1 μg/mL or 241.2 μM on HL-60) and **11** (44.3 μg/mL or 260.6 μM on K562 and 32.8 μg/mL or 192.9 μM on HL-60) is not so exciting. On the other hand, except three other new compounds **3**–**4** weakly inhibited the K562 and HL-60 cells with the IR% values larger than 20% at 100 μg/mL, all the others showed very weak effect on some of the tested four cancer cell lines with the IR% values lower than 20% at 100 μg/mL, as shown in Section 2.3. Aspyrone and asperlactone have been identified for some biological activities, including the remarkable insect growth regulator activity of asperlactone against *Tribolium castaneum* and *Nezara viridula* [48], the nematicidal activity of aspyrone on *Pratylenchus penetrans* [49], and the antifungal and antibacterial activities of both compounds on several fungal and bacterial strains [50]. Antibacterial activities were also reported for several chlorine containing derivatives from aspyrone and asperlactone by opening of the epoxy ring therein [22,51]. Although aspinonene and mono (*S*)- and (*R*)-MTRA esters of dihydroaspyrone (**10**) at C-9 have been reported to show a low cytotoxicity on mouse lymphocytic leukemia cells [19], there are few of reports recorded the antitumor activities for the compounds structurally closely related to **1**–**14**. The present bioassay results, as well as the report in the literature [19], seem to suggest that the branched C_9_ polyketides structurally related to **1**–**14** were likely worthy for further extended studies to obtain antitumor agents with more strong activity and higher selectivity.

## 3. Experimental Section

### 3.1. General Experimental

Melting point was measured on a Beijing Tiandiyu X-4 exact micro melting point apparatus (Tiandiyu science and technology Co., Ltd., Beijing, China) and the temperatures were not corrected. Optical rotations were measured on an Optical Activity Limited polAAr 3005 spectropolarimeter (Optical Activity Limited, Ramsey, UK). ESIMS was recorded on an Applied Biosystems API 3000 LC-MS spectrometer (AB SCIEX, Framingham, MA, USA) and HRESIMS was measured on an Agilent 6520 Q-TOF LC-MS spectrometer (Agilent Technologies, Santa Clara, CA, USA). IR spectra were taken on a Bruker Tensor-27 infrared spectrophotometer (Bruker, Karlsruhe, Germany). CD and ICD data were recorded on a Biologic Science MOS450 CD spectropolarimeter (Bio-Logic, Pont-de-Claix, France). 1D and 2D NMR spectra were obtained on a JEOL JNM-GX 400 (400 MHz ^1^H and 100 MHz ^13^C NMR) NMR spectrometer (JEOL Ltd., Tokyo, Japan). The chemical shifts of ^1^H and ^13^C NMR signals were recorded in δ values using the solvent signals (CDCl_3_: δ_H_ 7.26/δ_C_ 77.1; CD_3_OD: δ_H_ 3.31/δ_C_ 49.0) as references, respectively.

Precoated silica gel GF_254_ plates (10 cm × 20 cm, 0.25 mm thickness, Yantai Chemical Industrial Institute, Yantai, China) were used in TLC, and spots were detected under UV lights (254 and 365 nm) or by using the 10% sulfuric acid reagent or Vaughan’s reagent [39,52]. Silica gel H (100–200 mesh, Yantai Chemical Industrial Institute, Yantai, China), YMC*GEL^®^ ODS-A-HG (12 nm S-50 μm, YMC Co., Ltd., Kyoto, Japan), and Sephadex™ LH-20 (GE Healthcare, Uppsala, Sweden) were used for column chromatography. HPLC was performed on a Waters HPLC systems equipped with Waters 600 controller, Waters 600 pump, Waters 2414 refractive index detector, Waters 2996 (for analytical HPLC) or 2998 (for preparative HPLC) photodiode array detector, and Waters Empower™ software. The Capcell Pak C18 columns (UG120 Å, 4.6 × 250 mm and 20 × 250 mm; Shiseido Co., Ltd., Tokyo, Japan) were used in analytical and preparative HPLC, respectively.

Human chronic myelogenous leukemia K562 cell line was provided by Prof. Dr. Lili Wang (Beijing Institute of Pharmacology and Toxicology, Beijing, China). Human acute promyelocytic leukemia HL-60, human cervical cancer HeLa, and Human gastric adenocarcinoma BGC-823 cell lines were provided by Prof. Dr. Wenxia Zhou (Beijing Institute of Pharmacology and Toxicology). Fetal bovine serum was purchased from Tianjin Hao Yang Biological manufacture Co., Ltd. (Tianjin, China). The RPMI-1640 medium was purchased from Gibco (lot No. 1403238) and MTT from Amresco (lot No. 0793). Streptomycin (lot No. 071104) and penicillin (lot No. X1103302) were purchased from North China Pharmaceutical Group Corporation, China. Docetaxol (DOC, lot No.20110326) was purchased from Beijing Chimivo Technology Co., Ltd. (Beijing, China).

### 3.2. Producing Strain, Fermentation and Extraction

The producing fungal strain 16-02-1 was isolated from a deep-sea sediment, DY19-4-TVG11, which was collected at a Lau Basin hydrothermal vent (depth 2255 m, temperature 114 °C) in the southwest Pacific (south latitude 20.9280°, west longitude 176.2401°) during round-the-world ocean research of Dayangyihao in May 2007. This strain was identified as a species of the genus *Aspergillus* by sequence analysis of the ITS region of the rDNA and by morphological characteristics.

For fermentation of the 16-02-1 strain, a spore suspension was prepared using fresh spores by the method that we have previously reported [39,52] at first. Then, the fermentation was carried out in sixteen of 500 mL Erlenmeyer flasks, each containing 80 g of rice. Distilled water (120 mL) was added to each flask, and the contents were soaked overnight before autoclaving at 121 °C for 30 min. After cooling to room temperature, each flask was inoculated with 200 μL of the 16-02-1 spore suspension and incubated at 28 °C for 36 days. The fermented material was extracted repeatedly with EtOAc (3 × 6 L), and the organic solvent was evaporated under reduced pressure to obtain an EtOAc extract (21.9 g). The EtOAc extract inhibited K562 cells with an IR% of 76.8% at 100 μg/mL.

### 3.3. Isolation of 1–14

The EtOAc extract (21.9 g) was subjected to silica gel column (silica gel 140 g, bed 6.0 × 30 cm) chromatography by stepwise elution with b.p. 60–90 °C petroleum ether (*P*)–dichloromethane (d)–MeOH (M) to obtain three fractions: **Fr**-**4** (5.7 g, eluted by DM 30:1, IR% 55.9% at 100 μg/mL on K562 cells: the following IR% value in each bracket all indicate the IR% of the fraction at 100 μg/mL on K562 cells), **Fr**-**5** (5.4 g, eluted by DM 20:1, IR% 80.2%) and **Fr**-**6** (2.5 g, eluted by DM 9:1, IR% 78.6%).

**Fr**-**4** (5.7 g) was separated by Sephadex LH-20 column (bed 1.5 × 135 cm) chromatography using DM 1:1 as eluting solvent to obtain three fractions **Fr**-**4**-**1**–**Fr**-**4**-**3**. **Fr-4-1** (2.2 g, IR% 75.4%) was subjected to ODS column (bed 1.2 × 10 cm) and eluted with MeOH–H_2_O (20:80→90:10) to afford three fractions **Fr**-**4-1**-**1**–**Fr**-**4-1**-**3**. **Fr**-**4-1**-**2** was then subjected to preparative HPLC (Capcell Pak C18, 20 mm × 250 mm, room temperature; 20% MeOH at initial time 0 min→100% MeOH at 20 min→100% MeOH at 50 min; flow rate, 10 mL/min; detecting wave length 210 nm) to obtain **2** (6.5 mg, *t*_R_ = 14.1 min) and **8** (13 mg, *t*_R_ = 9.9 min). **Fr**-**4-2** (2.7 g, IR% 42.3%) was subjected to ODS column (bed 1.2 × 10 cm) chromatography and stepwise elution by MeOH–H_2_O (20:80→90:10) afforded three fractions **Fr**-**4-2**-**1**–**Fr**-**4-2**-**3**. A preparative HPLC separation (Capcell Pak C18 column, 20 mm × 250 mm, at the room temperature; 20% MeOH at initial time 0 min→100% MeOH at 30 min; flow rate, 10 mL/min; detecting wave length 210 nm) of **Fr**-**4-2**-**3** (1.8 g, IR% 40.1%) gave **1** (9 mg, *t*_R_ = 9.2 min) and five fractions **Fr**-**4-2**-**3-1**–**Fr**-**4-2**-**3-5**. Separation of **Fr**-**4-2**-**3-3** (28 mg) by preparative HPLC at the same condition except for the mobile phase MeOH–H_2_O 95:5 gave **5** (6.5 mg, *t*_R_ = 35.5 min), and separation of **Fr**-**4-2**-**3-4** (28 mg) by preparative HPLC at the same condition using the mobile phase MeOH–H_2_O 94:6 afforded **9** (7 mg, *t*_R_ = 33.3 min).

**Fr**-**5** (5.4 g) was subjected Sephadex LH-20 column (bed 1.5 × 135 cm) chromatography eluted with DM 1:1 to separate into three fractions **Fr**-**5**-**1**–**Fr**-**5**-**3**. **Fr**-**5**-**2** (1.2 g, IR% 40.1%) was subjected to preparative HPLC (Capcell Pak C18 column, 20 mm × 250 mm, room temperature; 20% MeOH at initial time 0 min→100% MeOH at 50 min; flow rate, 10 mL/min; detecting wave length 210 nm) to obtain **11** (12 mg, *t*_R_ = 13.2 min) and **10** (141 mg, *t*_R_ = 11.8 min).

**Fr**-**6** (2.5 g) was subjected to ODS column (bed 1.2 × 10 cm) chromatography eluted with MeOH–H_2_O (20:80→90:10) to give three fractions **Fr**-**6-1**–**Fr**-**6-3**. **Fr**-**6-2** (1.7 g, IR% 62.5%) was separated by preparative HPLC (Capcell Pak C18 column, 20 mm × 250 mm, room temperature; 10% MeOH at initial time 0 min→100% MeOH at 50 min; flow rate, 10 mL/min; detecting wave length 210 nm) to obtain **6**/**7** (19 mg, *t*_R_ = 13.6 min), **13** (19 mg, *t*_R_ = 15.8 min) and a fraction (29 mg, *t*_R_ = 6 min) containing **3** and **4**. This fraction (29 mg) was further separated by preparative HPLC at the same condition except for the mobile phase MeOH–H_2_O 90:10 to obtain **3** (9 mg, *t*_R_ = 13.6 min) and **4** (7 mg, *t*_R_ = 16.0 min). **Fr**-**6-3** (872 mg, IR% 30.1%) was separated by preparative HPLC at the same conditions as described for **Fr**-**6-2** to obtain **12** (21 mg, *t*_R_ = 18.8 min) and **14** (31 mg, *t*_R_ = 23.7 min).

### 3.4. Physicochemical and Spectroscopic Data of 1–14

Aspiketolactonol (**1**): Colorless oil (MeOH), 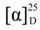 +10.5 (*c* 1.2, MeOH). Positive ion ESIMS *m*/*z*: 185 [M + H]^+^, 207 [M + Na]^+^; Negative ion ESIMS *m*/*z*: 183 [M − H]^−^. Positive ion HRESIMS *m*/*z*: measured 185.0811 [M + H]^+^, calculated for C_9_H_13_O_4_ [M + H]^+^ 185.0814; measured 207.0631 [M + Na]^+^, calculated for C_9_H_12_O_4_Na [M + Na]^+^ 207.0633. UV λ_max_ nm in MeOH: 213 nm. IR ν_max_ cm^−1^ (Diamond ATR crystal): 3417, 3090, 2980, 2938, 2906, 1752, 1722, 1656, 1422, 1360, 1323, 1207, 1166, 1138, 1081, 1024, 982, 935, 896. CD Δε (nm): 0 (192.5), −0.44 (198.0), −0.30 (207.5), −0.32 (209.5), −0.21 (215.0), −0.23 (216.5), 0 (226.0), +0.34 (233.0), +0.07 (244.5), 0 (282). ^1^H and ^13^C NMR data: Table 1 and Table 2.

Aspilactonol A (**2**): Colorless oil (MeOH), 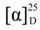 +24.6 (*c* 0.23, MeOH). Positive ion ESIMS *m*/*z*: 171 [M + H]^+^, 193 [M + Na]^+^. Positive ion HRESIMS *m*/*z*: measured 171.1015 [M + H]^+^, calculated for C_9_H_15_O_3_ [M + H]^+^ 171.1021; measured 193.0836 [M + Na]^+^, calculated for C_9_H_14_O_3_Na [M + Na]^+^ 193.0841; measured 209.0573 [M + K]^+^, calculated for C_9_H_14_O_3_K [M + Na]^+^ 209.0580. UV λ_max_ nm in MeOH: 210 nm. IR ν_max_ cm^−1^ (Diamond ATR crystal): 3436, 3080, 2971, 2932, 1746, 1653, 1449, 1406, 1374, 1320, 1201, 1030, 960, 870, 786. CD Δε (nm): 0 (191.0), −0.49 (198.0), −0.30 (206.0), −0.33 (208.5), −0.25 (214.0), −0.26 (215.5), 0 (219.5), +0.70 (225.0), 0 (229.5), −0.60 (237.5), −0.59 (240.5), 0 (279.5). ^1^H and ^13^C NMR data: Table 1 and Table 2.

Aspilactonol B (**3**): Colorless oil (MeOH), 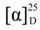 +34.5 (*c* 0.41, MeOH). Positive ion ESIMS *m*/*z*: 203 [M + H]^+^, 225 [M + Na]^+^; Negative ion ESIMS *m*/*z*: 201 [M − H]^−^. Positive ion HRESIMS *m*/*z*: measured 203.0919 [M + H]^+^, calculated for C_9_H_15_O_5_ [M + H]^+^ 203.0919; measured 225.0736 [M + Na]^+^, calculated for C_9_H_14_O_5_Na [M + Na]^+^ 225.0739. UV λ_max_ nm in MeOH: 214 nm. IR ν_max_ cm^−1^ (Diamond ATR crystal): 3388, 3095, 2978, 2936, 2900, 2831, 1742, 1653, 1451, 1406, 1378, 1314, 1200, 1136, 1069, 996, 934, 894, 870, 830, 803, 770, 721, 698. CD Δε (nm): 0 (192.0), −0.78 (197.0), 0 (206.5), +2.02 (226.5), 0 (282.5). ^1^H and ^13^C NMR data: Table 1 and Table 2.

Aspilactonol C (**4**): Colorless oil (MeOH), 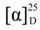 +27.2 (*c* 0.32, MeOH). Positive ion ESIMS *m*/*z*: 203 [M + H]^+^, 225 [M + Na]^+^; Negative ion ESIMS *m*/*z*: 201 [M − H]^−^. Positive ion HRESIMS *m*/*z*: measured 203.0910 [M + H]^+^, calculated for C_9_H_15_O_5_ [M + H]^+^ 203.0919; measured 225.0732 [M + Na]^+^, calculated for C_9_H_14_O_5_Na [M + Na]^+^ 225.0739; measured 241.0476 [M + K]^+^, calculated for C_9_H_14_O_5_K [M + K]^+^ 241.0478. UV λ_max_ nm in MeOH: 214 nm. IR ν_max_ cm^−1^ (Diamond ATR crystal): 3379, 3092, 2979, 2935, 1737, 1651, 1450, 1402, 1377, 1300, 1200, 1133, 1063, 994, 933, 894, 868, 826, 763, 693. CD Δε (nm): 0 (192.0), −0.43 (196.5), −0.39 (197.0), −0.41 (197.5), 0 (201.5), +0.37 (210.5), +1.64 (225.5), +1.65 (226.0), 0 (242.0), −0.19 (251.5), 0 (275.0). ^1^H and ^13^C NMR data: Table 1 and Table 2.

Aspilactonol D (**5**): Colorless oil (MeOH), 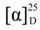 −1.7 (*c* 0.38, MeOH). Positive ion ESIMS *m*/*z*: 217 [M + H]^+^, 239 [M + Na]^+^. Positive ion HRESIMS *m*/*z*: measured 217.1071 [M + H]^+^, calculated for C_10_H_17_O_5_ [M + H]^+^ 217.1076; measured 239.0890 [M + Na]^+^, calculated for C_10_H_16_O_5_Na [M + Na]^+^ 239.0895; measured 255.0635 [M + K]^+^, calculated for C_10_H_16_O_5_K [M + K]^+^ 255.0635. UV λ_max_ nm in MeOH: 213 nm. IR ν_max_ cm^−1^ (Diamond ATR crystal): 3401, 3088, 2978, 2937, 2902, 2832, 1750, 1655, 1451, 1378, 1298, 1256, 1200, 1136, 1108, 1066, 1022, 977, 950, 936, 894, 853, 833, 803, 780, 717. CD Δ*ε* (nm): 0 (193.5), −0.29 (196.5), 0 (203.5), +1.41 (231.5), 0 (271.0). ^1^H and ^13^C NMR data: Table 1 and Table 2.

Aspilactonols E/F (**6**/**7**): Colorless oil (MeOH), 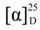 +78.1 (*c* 1.00, MeOH). Positive ion ESIMS *m*/*z*: 187 [M + H]^+^, 209 [M + Na]^+^; Negative ion ESIMS *m*/*z*: 185 [M − H]^−^. Positive ion HRESIMS *m*/*z*: measured 187.0967 [M + H]^+^, calculated for C_9_H_15_O_5_ [M + H]^+^ 187.0970; measured 209.0789 [M + Na]^+^, calculated for C_9_H_14_O_5_Na [M + Na]^+^ 209.0790. UV λ_max_ nm in MeOH: 213 nm. IR ν_max_ cm^−1^ (Diamond ATR crystal): 3396, 3093, 2975, 2934, 1748, 1651, 1456, 1417, 1377, 1205, 1127, 1070, 990, 942, 882, 841, 677. CD Δε (nm): 0 (185.0), −0.55 (196.5), −0.09 (201.5), −0.05 (202.5), −0.07 (214.0), 0 (216.5), +2.78 (228.5), 0 (280.5). ^1^H and ^13^C NMR data: Table 1 and Table 2.

Aspyronol (**9**): Colorless oil (MeOH), 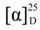 −41.6 (*c* 0.22, MeOH). Positive ion ESIMS *m*/*z*: 217 [M + H]^+^, 239 [M + Na]^+^. Positive ion HRESIMS *m*/*z*: measured 217.1073 [M + H]^+^, calculated for C_10_H_17_O_5_ [M + H]^+^ 217.1076; measured 239.0896 [M + Na]^+^, calculated for C_10_H_16_O_5_Na [M + Na]^+^ 239.0895; measured 255.0634 [M + K]^+^, calculated for C_10_H_16_O_5_K [M + K]^+^ 255.0635. UV λ_max_ nm in MeOH: 208 nm. IR ν_max_ cm^−1^ (Diamond ATR crystal): 3398, 2982, 2938, 2905, 2835, 1714, 1649, 1451, 1384, 1309, 1216, 1044, 1018, 783, 721, 669. CD Δε (nm): 0 (192.0), −0.53 (196.5), −0.72 (209.5), −2.81 (223.5), 0 (242.0), −1.22 (263.0), 0 (301.0). ^1^H and ^13^C NMR data: Table 3.

Epiaspinonediol (**11**): Colorless oil (MeOH), 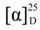 −5.4 (*c* 0.14, MeOH). Positive ion ESIMS *m*/*z*: 193 [M + Na]^+^, 209 [M + K]^+^. Positive ion HRESIMS *m*/*z*: measured 171.1003 [M + H]^+^, calculated for C_9_H_15_O_3_ [M + H]^+^ 171.1021; measured 193.0842 [M + Na]^+^, calculated for C_9_H_14_O_3_Na [M + Na]^+^ 193.0841. ^1^H NMR (400 MHz, CD_3_OD) δ: 7.20 (1H, d, *J* = 16.2 Hz, H-4), 6.41 (1H, d, *J* = 16.2 Hz, H-3), 6.05 (1H, d, *J* = 8.4 Hz, H-6), 4.77 (1H, dq, *J* = 8.4, 6.4 Hz, H-7), 4.33 (2H, s, H-9), 2.31 (3H, s, H-1), 1.29 (3H, d, *J* = 6.4 Hz, H-8). ^13^C NMR (100 MHz, CD_3_OD) δ: 201.6 (C-2), 148.3 (C-6), 147.5 (C-4), 137.0 (C-5), 128.4 (C-3), 64.8 (C-7), 56.9 (C-9), 27.3 (C), 23.6 (C-8).

The data for five known compounds, **8**, **10** and **12**–**14**, and the spectra of the new compounds, **1**–**7**, **9** and **11**, are all given in the Supplementary Information.

### 3.5. Preparation of (S)- and (R)-MTPA Esters of 1, 5 and 9

Each of duplicate **2** (0.3 mg, 1.8 μmol), **5** (0.5 mg, 2.3 μmol) and **9** (0.3 mg, 1.4 μmol) in 0.2 mL unhydrous pyridine-*d*_6_ in NMR tubes was reacted with (*S*)- and (*R*)-MPTA-Cl (1.65 μL, 8.8 μmol for **2**; 4.40 μL, 23.1 μmol for **5**; 2.63 μL, 13.9 μmol for **9**), respectively. The reaction was performed at room temperature for 72 h. Then, the ^1^H NMR data of the (*S*)- and (*R*)-MTPA esters were obtained without purification.

Compound **2a** (pyridine-*d*_5_, 400 MHz) δ: 7.10 (1H, br s, H-4), 5.30 (1H, m, H-9), 4.99 (1H, m, H-5), 2.41 (2H, m, H-7), 1.87 (2H, m, H-8), 1.24 (3H, d, *J* = 6.5 Hz, H-6), 1.23 (3H, d, *J* = 6.2 Hz, H-10).

Compound **2b** (pyridine-*d*_5_, 400 MHz) δ: 7.02 (1H, br s, H-4), 5.31 (1H, m, H-9), 4.97 (1H, m, H-5), 2.28 (2H, m, H-7), 1.84 (2H, m, H-8), 1.31 (3H, d, *J* = 6.3 Hz, H-10), 1.22 (3H, d, *J* = 6.9 Hz, H-6).

Compound **5a** (pyridine-*d*_5_, 400 MHz) δ: 5.87 (1H, m, H-9), 5.25 (1H, m, H-5), 4.59 (1H, m, H-6), 4.42 (1H, br d, *J* = 4.0 Hz, H-8), 3.24 (3H, s, OCH_3_), 1.79 (3H, d, *J* = 7.4 Hz, H-7), 1.37 (3H, d, *J* = 6.5 Hz, H-10).

Compound **5b** (pyridine-*d*_5_, 400 MHz) δ: 5.83 (1H, m, H-9), 5.33 (1H, m, H-5), 4.60 (1H, m, 6-H), 4.54 (1H, br d, *J* = 3.9 Hz, H-8), 3.34 (3H, s, OCH_3_), 1.80 (3H, d, *J* = 7.4 Hz, H-7), 1.31 (3H, d, *J* = 6.5 Hz, H-10).

Compound **9a** (pyridine-*d*_5_, 400 MHz) δ: 7.18 (1H, m, H-4), 5.93 (1H, m, H-5), 5,7860 (1H, m, H-9), 4.58 (1H, m, H-8), 4.57 (1H, m, H-6), 3.19 (3H, s, OCH_3_), 1.39 (3H, d, *J* = 6.3 Hz, H-7), 1.28 (3H, d, *J* = 6.4 Hz, H-10).

Compound **9b** (pyridine-*d*_5_, 400 MHz) δ: 7.19 (1H, m, H-4), 6.03 (1H, m, H-5), 5.7827 (1H, m, H-9), 4.88 (1H, m, H-6), 4.72 (1H, m, H-8), 3.29 (3H, s, OCH_3_), 1.45 (3H, d, *J* = 6.5 Hz, H-7), 1.22 (3H, d, *J* = 6.6 Hz, H-10).

### 3.6. Measurement of ICD Spectra of 3 and 4 Using Mo_2_(OAc)_4_

ICD was measured according to our previous procedure [39] using spectroscopy grade unhydrous DMSO. A mixture of the ligand (**3** or **4**) and Mo_2_(OAc)_4_ in DMSO in an approximate 1:2 molar ratio was subjected to the ICD measurement. The first CD spectrum was recorded immediately after mixing, and its time evolution was monitored until stationary ICD was reached about 10 min after mixing. After the inherent CD data of the compound were subtracted, the ICD spectrum was normalized to the molar concentration of **3** or **4** and is presented as the Δε′ values. The observed signs of the bands II and IV in the ICD were correlated to the absolute configuration of the 1,2-diol moiety.

### 3.7. MTT Assay

All samples and DOC were dissolved in MeOH to prepare the MeOH solution at 10.0 mg/mL, and serial dilutions were made for compounds **9** and **11**. These solutions were subjected to MTT assay. DOC was used as positive control and MeOH was used as blank control. The assay was run in triplicate on human cancer K562, HL-60, HeLa and BGC-823 cell lines by the method that we have previously reported [52]. The OD was read at 570 nm, and the IR% value was calculated using the OD mean values by the formula, IR% = (OD_control_ − OD_sample_)/OD_control_ × 100%. The IC_50_ for **9** and **11** was obtained from their IR% values at different concentrations.

## 4. Conclusions

Chemical investigation on a deep sea-sourced *Aspergillus* sp. 16-02-1 has resulted in the elucidation of 14 secondary metabolites, including nine new branched C_9_ polyketides, named aspiketolactonol (**1**), aspilactonols A–F (**2**–**7**), aspyronol (**9**) and epiaspinonediol (**11**), and five known branched polyketides identified as (*S*)-2-(2′-hydroxyethyl)-4-methyl-γ-butyrolactone (**8**), dihydroaspyrone (**10**), aspinotriol A (**12**), aspinotriol B (**13**) and chaetoquadrin F (**14**). Structures of the new compounds, including their absolute configurations, were determined by extensive spectroscopic methods, especially the 2D NMR, CD, ICD and Mosher’s ^1^H NMR analyses. Compound **8** was isolated from the natural sources for the first time, and the possible biosynthetic pathways for **1**–**14** were also proposed and discussed. Compounds **1**–**14** inhibited human cancer K562, HL-60, HeLa and BGC-823 cells to varying extents.

## Supplementary Files

Supplementary File 1 Supplementary Information (PDF, 6176 KB)

## References

[1] Blunt J.W., Copp B.R., Keyzers R.A., Munro M.H.G., Prinsep M.R. (2014). Marine natural products. Nat. Prod. Rep..

[2] Blunt J.W., Copp B.R., Keyzers R.A., Munro M.H.G., Prinsep M.R. (2013). Marine natural products. Nat. Prod. Rep..

[3] Blunt J.W., Copp B.R., Keyzers R.A., Munro M.H.G., Prinsep M.R. (2012). Marine natural products. Nat. Prod. Rep..

[4] Blunt J.W., Copp B.R., Munro M.H.G., Northcote P.T., Prinsep M.R. (2011). Marine natural products. Nat. Prod. Rep..

[5] Blunt J.W., Copp B.R., Munro M.H.G., Northcote P.T., Prinsep M.R. (2010). Marine natural products. Nat. Prod. Rep..

[6] Bugni T.S., Ireland C.M. (2004). Marine-derived fungi: A chemically and biologically diverse group of microorganisms. Nat. Prod. Rep..

[7] Saleem M., Ali M.S., Hussain S., Jabbar A., Ashraf M., Lee Y.S. (2007). Marine natural products of fungal origin. Nat. Prod. Rep..

[8] Bhatnagar I., Kim S.-K. (2010). Immense essence of excellence: Marine microbial bioactive compounds. Mar. Drugs.

[9] Rateb M.E., Ebel R. (2011). Secondary metabolites of fungi from marine habitats. Nat. Prod. Rep..

[10] Pejin B., Jovanović K.K., Mojović M., Savić A.G. (2013). New and highly potent antitumor natural products from marine-derived fungi: Covering the period from 2003 to 2012. Curr. Top. Med. Chem..

[11] Newman D.J., Cragg G.M. (2014). Marine-sourced anti-cancer and cancer pain control agents in clinical and late preclinical development. Mar. Drugs.

[12] Skropeta D. (2008). Deep-sea natural products. Nat. Prod. Rep..

[13] Li D.H., Cai S.X., Zhu T.J., Wang F.P., Xiao X., Gu Q.Q. (2010). Three new sorbicillin trimers, trisorbicillinones B, C, and D, from a deep ocean sediment derived fungus, *Phialocephala* sp. FL30r. Tetrahedron.

[14] Li Y., Ye D.Z., Shao Z.Z., Cui C.B., Che Y.S. (2012). A sterol and spiroditerpenoids from a *Penicillium* sp. isolated from a deep sea sediment sample. Mar. Drugs.

[15] Wang F.Z., Huang Z., Shi X.F., Chen Y.C., Zhang W.M., Tian X.P., Li J., Zhang S. (2012). Cytotoxic indole diketopiperazines from the deep sea-derived fungus *Acrostalagmus luteoalbus* SCSIO F457. Bioorg. Med. Chem. Lett..

[16] Chen Y., Mao W.J., Wang B.F., Zhou L., Gu Q.Q., Chen Y.L., Zhao C.Q., Li N., Wang C.Y., Shan J.M. (2013). Preparation and characterization of an extracellular polysaccharide produced by the deep-sea fungus *Penicillium griseofulvum*. Bioresour. Technol..

[17] Chen X.W., Li C.W., Hua W., Wu C.J., Cui C.B., Zhu T.J., Gu Q.Q. (2013). Metabolites of *Aspergillus* sp. 16-02-1 isolated from a deep sea sediment and preliminary test of their antitumor and antifungal activities. Chin. J. Mar. Drugs.

[18] Harmange J.C., Figadėre B., Hocquemiller R. (1991). Enantiospecific preparation of the lactone fragment of murisolin. Tetrahedron: Asymmetry.

[19] Kito K., Ookura R., Yoshida S., Namikoshi M., Ooi T., Kusumi T. (2007). Pentaketides relating to aspinonene and dihydroaspyrone from a marine-derived fungus, *Aspergillus ostianus*. J. Nat. Prod..

[20] Fujimoto H., Nozawa M., Okuyama E., Ishibashi M. (2003). Six new constituents from an ascomycete, *Cheatomium quadrangulatum*, found in a screening study focused on monoamine oxidase inhibitory activity. Chem. Pharm. Bull..

[21] Garson M.J., Staunton J. (1984). New polyketide metabolites from *Aspergmus melleus*: Structural and stereochemical studies. J. Chem. Soc. Perkin Trans. I.

[22] Namikoshi M., Negishi R., Nagai H., Dimitrenok M., Kobayashi H. (2003). Three new chlorine containing antibiotics from a marine-derived fungus *Aspergillus ostianus* collected in Pohnpei. J. Antibot..

[23] Sy A.A., Swenson D.C., Gloer J.B., Wicklow D.T. (2008). Botryolides A–E, decarestrictine analogues from a fungicolous *Botryotrichum* sp. (NRRL 38180). J. Nat. Prod..

[24] Buchanan M., Hashimoto T., Takaoka S., Asakawa Y. (1995). (+)-Osmundalactone, γ-lactones and spiromentins from the fungus *Paxillus atrotomentosus*. Phytochemistry.

[25] Franck X., Araujo M.E.V., Jullian J.-C., Hocquemiller R., Figadère B. (2001). Synthesis and structure determination of *iso*-cladospolide B. Tetrahedron Lett..

[26] Uchida I., Kuriyama K. (1974). The π-π* circular dichroism of α,β-unsaturated γ-lactones. Tetrahedron Lett..

[27] Gawronski J.K., Oeveren A.V., Deen H.V.D., Leung C.W., Feringa B.L. (1996). Simple circular dicroic method for the determination of absolute configuration of 5-substitutaed 2(5*H*)-furanones. J. Org. Chem..

[28] Beecham A.F. (1972). The CD of α,β-unsaturated γ-lactones. Tetrahedron.

[29] Lee C.-L., Chang F.-R., Hsieh P.-W., Chiang M.-Y., Wu C.-C., Huang Z.-Y., Lan Y.-H., Chen M., Lee K.-H., Yen H.-F. (2008). Cytotoxic *ent*-abietane diterpenes from *Gelonium aequoreum*. Phytochemistry.

[30] Dale J.A., Mosher H.S. (1973). Nuclear magnetic resonance enantiomer regents. Configurational correlations via nuclear magnetic resonance chemical shifts of diastereomeric mandelate, *O*-methylmandelate, and α-methoxy-α-trifluoromethylphenylacetate (MTPA) esters. J. Am. Chem. Soc..

[31] Ohtani I., Kusumi T., Kashman Y., Kakisawa H. (1991). High-field FT NMR application of Mosher’s method. The absolute configurations of marine terpenoids. J. Am. Chem. Soc..

[32] Jarvis B.B., Stahly G.P., Pavanasasivam G., Midiwo J.O., DeSilva T., Holmlund C.E., Mazzola E.P., Geoghegan R.F. (1982). Isolation and characterization of the trichoverroids and new roridins and verrucarins. J. Org. Chem..

[33] Jarvis B.B., Comezoglu S.N., Rao M.M., Pena N.B. (1987). Isolation of macrocyclic trichothecenes from a large-scale extract of *Baccharis megapotamica*. J. Org. Chem..

[34] Takeshita M., Sato T. (1989). Synthesis of optically active 1-phenyl-1,2-propanediol by use of Baker’s yeast. Chem. Pharm. Bull..

[35] Ayer W.A., Trifonov L.S. (1993). Metabolites of *Peniophora polygonia*, part 2. Some aromatic compounds. J. Nat. Prod..

[36] Jarvis B.B., Wang S., Ammon H.L. (1996). Trichoverroid stereoisomers. J. Nat. Prod..

[37] Bari L.D., Pescitelli G., Pratelli C., Pini D., Salvadori P. (2001). Determination of absolute configuration of acyclic 1,2-diols with Mo_2_(OAc)_4_. 1. Snatzke’s method revisited. J. Org. Chem..

[38] Frelek J., Ruśkowska P., Suszczyńska A., Szewczyk K., Osuch A., Jarosz S., Jagodziński J. (2008). Configurational assignment of sugar *erythro*-1,2-diols from their electronic circular dichroism spectra with dimolybdenum tetraacetate. Tetrahedron Asymmetry.

[39] Xia M.-W., Cui C.-B., Li C.-W., Wu C.-J. (2014). Three new and eleven known unusual C25 steroids: Activated production of silent metabolites in a marine-derived fungus by chemical mutagenesis strategy using diethyl sulphate. Mar. Drugs.

[40] Zhang G.J. (2011). Studies on the Meroterpenoidal Constituents from Two Marine-Derived Fungal Strains. Ph. D. Thesis.

[41] Staunton J., Sutkowski A.C. (1991). Biosynthesis of aspyrone, a metabolite of *Aspergillus melleus*: Advanced precursor studies to identify the product of the polyketide synthesis. J. Chem. Soc. Chem. Commun..

[42] Staunton J., Sutkowski A.C. (1991). The polyketide synthase (PKS) of aspyrone biosynthesis evidence: Evidence for the enzyme bound intermediates from incorporation studies with *N*-acetylcysteamine thioesters in intact cells of *Aspergillus melleus*. J. Chem. Soc. Chem. Commun..

[43] Jacobs A., Staunton J., Sutkowski A.C. (1991). Aspyrone biosynthesis in *Aspergillus melleus*: Identification of the intermediates formed on the polyketide synthase (PKS) in the first chain extension cycle leading to crotonate. J. Chem. Soc. Chem. Commun..

[44] Brereton R., Garson M., Staunton J. (1984). Biosynthesis of fungal metabolites: Asperlactone and its relationship to other metabolites of *Aspergillus melleus*. J. Chem. Soc. Perkin Trans. I.

[45] Ahmed S.A., Simpson T.J., Staunton J., Sutkowski A.C., Trimble L.A., Vederas J.C. (1985). Biosynthesis of aspyrone and asperlactone, petaketide metabolites of *Aspergillus melleus*. Incorporation studies with [1-1^3^C,^18^O_2_] acetate and ^18^O_2_ gas. J. Chem. Soc. Chem. Commun..

[46] Staunton J., Sutkowski A.C. (1991). ^17^O NMR biosynthetic studies: Aspyrone, asperlactone and isoasperlactone, metabolites of *Aspergillus melleus*. J. Chem. Soc. Chem. Commun..

[47] Fuchser J., Thiericke R., Zeeck A. (1995). Biosynthesis of aspinonene, a branched pnetaketide produced by *Aspergillus ochraceus*, related to aspyrone. J. Chem. Soc. Perkin Trans. I.

[48] Balcells M., Canela R., Coll J., Sanchís V., Torres M. (1995). Effect of fungal metabolites and some derivatives against *Tribolium castaneum* (Herbst) and *Nezara viridula* (L.). Pesitic. Sci..

[49] Kimura Y., Nakahara S., Fujioka S. (1996). Aspyrone, a nematicidal compound isolated from the fungus, *Aspergillus melleus*. Biosci. Biotech. Biochem..

[50] Torres M., Balcells M., Sala N., Sanchís V., Canela R. (1998). Bactericidal and fungicidal activity of *Aspergillus ochraceus* metabolites and some derivatives. Pesitic. Sci..

[51] Zhang D., Yang X., Kang J.S., Choi H.D., Son B.H. (2008). Chlorohydroaspyrones A and B, antibacterial aspyrone derivatives from the marine-derived fungus *Exophiala* sp. J. Nat. Prod..

[52] Wu C.-J., Li C.-W., Cui C.-B. (2014). Seven new and two known lipopeptides as well as five known polyketides: The activated production of silent metabolites in a marine-derived fungus by chemical mutagenesis strategy using diethyl sulphate. Mar. Drugs.

