# Supplementary material for: Nine New and Five Known Polyketides Derived from a Deep Sea-Sourced Aspergillus sp. 16-02-1
Source: Mar Drugs. 2014 May 27;12(6):3116–37. doi: 10.3390/md12063116 (PMC4071568; doi:10.3390/md12063116)
Supplement: Supplementary File 1 — Supplementary Information (PDF, 6176 KB) [file marinedrugs-12-03116-s001.pdf]

Supplementary Information

S1. Physicochemical and spectroscopic data of five known compounds, **8**, **10**, and **12–14**.
Table S1. 400 MHz ^{1}H NMR and 100 MHz ^{13}C NMR data of **1** in $CDCl_3$.
Table S2. 400 MHz ^{1}H NMR and 100 MHz ^{13}C NMR data of **2** in $CDCl_3$.
Table S3. 400 MHz ^{1}H NMR and 100 MHz ^{13}C NMR data of **3** in CD_3OD.
Table S4. 400 MHz ^{1}H NMR and 100 MHz ^{13}C NMR data of **4** in CD_3OD.
Table S5. 400 MHz ^{1}H NMR and 100 MHz ^{13}C NMR data of **5** in CD_3OD.
Table S6. 400 MHz ^{1}H NMR and 100 MHz ^{13}C NMR data of **6/7** in CD_3OD.
Table S7. 400 MHz ^{1}H NMR and 100 MHz ^{13}C NMR data of **9** in CD_3OD.
Figure S1. HPLC analysis of **1**, **3**, **4** and **6/7** after treatment in a water-saturated EtOAc at the conditions simulated their extraction conditions.
Figure S2. Positive ion ESI-MS spectrum of **1**.
Figure S3. Positive ion HR-ESI-MS spectrum of **1**.
Figure S4. IR spectrum of **1**.
Figure S5. 400 MHz ^{1}H NMR spectrum of **1** in $CDCl_3$.
Figure S6. 100 MHz ^{13}C NMR spectrum of **1** in $CDCl_3$.
Figure S7. HMQC spectrum of **1** in $CDCl_3$.
Figure S8. ^{1}H–^{1}H COSY spectrum of **1** in $CDCl_3$.
Figure S9. HMBC spectrum of **1** in $CDCl_3$.
Figure S10. Positive ion ESI-MS spectrum of **2**.
Figure S11. Positive ion HR-ESI-MS spectrum of **2**.
Figure S12. IR spectrum of **2**.
Figure S13. 400 MHz ^{1}H NMR spectrum of **2** in $CDCl_3$.
Figure S14. 100 MHz ^{13}C NMR spectrum of **2** in $CDCl_3$.
Figure S15. ^{1}H–^{1}H COSY spectrum of **2** in $CDCl_3$.
Figure S16. HMQC spectrum of **2** in $CDCl_3$.
Figure S17. HMBC spectrum of **2** in $CDCl_3$.
Figure S18. 400 MHz ^{1}H NMR of the (*S*)- and (*R*)-MTPA esters of **2** in pyridine-d_5.
Figure S19. Positive ion ESI-MS spectrum of **3**.
Figure S20. Positive ion HR-ESI-MS spectrum of **3**.
Figure S21. IR spectrum of **3**.
Figure S22. 400 MHz ^{1}H NMR spectrum of **3** in CD_3OD.
Figure S23. 100 MHz ^{13}C NMR spectrum of **3** in CD_3OD.
Figure S24. HMQC spectrum of **3** in CD_3OD.
Figure S25. ^{1}H–^{1}H COSY spectrum of **3** in CD_3OD.
Figure S26. HMBC spectrum of **3** in CD_3OD.
Figure S27. Positive ion ESI-MS spectrum of **4**.
Figure S28. Positive ion HR-ESI-MS spectrum of **4**.
Figure S29. IR spectrum of **4**.
Figure S30. 400 MHz ^{1}H NMR spectrum of **4** in CD_3OD.
Figure S31. 100 MHz ^{13}C NMR spectrum of **4** in CD_3OD.
Figure S32. HMQC spectrum of **4** in CD_3OD.

Figure S33. ^1H–^1H COSY spectrum of **4** in CD_3OD.
Figure S34. HMBC spectrum of **4** in CD_3OD.
Figure S35. Positive ion ESI-MS spectrum of **5**.
Figure S36. Positive ion HR-ESI-MS spectrum of **5**.
Figure S37. IR spectrum of **5**.
Figure S38. 400 MHz ^1H NMR spectrum of **5** in CD_3OD.
Figure S39. 100 MHz ^{13}C NMR spectrum of **5** in CD_3OD.
Figure S40. HMQC spectrum of **5** in CD_3OD.
Figure S41. ^1H–^1H COSY spectrum of **5** in CD_3OD.
Figure S42. HMBC spectrum of **5** in CD_3OD.
Figure S43. 400 MHz ^1H NMR spectrum of the (*S*)- and (*R*)-MTPA esters of **5** in pyridine-d_5.
Figure S44. Positive ion ESI-MS spectrum of **6**/**7**.
Figure S45. Positive ion HR-ESI-MS spectrum of **6**/**7**.
Figure S46. IR spectrum of **6**/**7**.
Figure S47. 400 MHz ^1H NMR spectrum of **6**/**7** in CD_3OD.
Figure S48. 100 MHz ^{13}C NMR spectrum of **6**/**7** in CD_3OD.
Figure S49. HMQC spectrum of **6**/**7** in CD_3OD.
Figure S50. ^1H–^1H COSY spectrum of **6**/**7** in CD_3OD.
Figure S51. HMBC spectrum of **6**/**7** in CD_3OD.
Figure S52. Positive ion ESI-MS spectrum of **9**.
Figure S53. Positive ion HR-ESI-MS spectrum of **9**.
Figure S54. IR spectrum of **9**.
Figure S55. 400 MHz ^1H NMR spectrum of **9** in CD_3OD.
Figure S56. 100 MHz ^{13}C NMR spectrum of **9** in CD_3OD.
Figure S57. HMQC spectrum of **9** in CD_3OD.
Figure S58. ^1H–^1H COSY spectrum of **9** in CD_3OD.
Figure S59. HMBC spectrum of **9** in CD_3OD.
Figure S60. 400 Hz ^1H NMR spectrum of **9** in $CDCl_3$.
Figure S61. ^1H–^1H COSY spectrum of **9** in $CDCl_3$.
Figure S62. Difference NOE spectrum of **9** in $CDCl_3$.
Figure S63. 400 M Hz ^1H NMR spectrum of the (*S*)- and (*R*)-MTPA esters of **9** in pyridine-d_6.
Figure S64. Positive ion ESI-MS spectrum of **11**.
Figure S65. Positive ion HR-ESI-MS spectrum of **11**.
Figure S66. 400 Hz ^1H NMR spectrum of **11** in CD_3OD.
Figure S67. 100 Hz ^{13}C NMR spectrum of **11** in CD_3OD.

S1. Physicochemical and Spectroscopic Data of Five Known Compounds, 8, 10, and 12–14.

(*S*)-2-(2'-hydroxyethyl)-4-methyl-γ-butyrolactone (**8**): Colorless oil (MeOH), $[\alpha]_D^{25}$ +55.2 (*c* 0.54, MeOH). Positive ion ESIMS *m*/*z*: 143 $[M + H]^+$, 165 $[M + Na]^+$; Negative ion ESIMS *m*/*z*: 141 $[M - H]^-$. CD Δε (nm): 0 (192.5), −0.29 (198.0), −0.22 (202.5), −0.29 (206.0), −0.18 (210.5), 0 (217.5), +0.54 (224.0), 0 (229.0), −0.40 (237.0), −0.39 (240.5), −0.38 (242.0), 0 (275.0). ^{1}H NMR (400 MHz, acetone-d_6) δ: 7.37 (1H, dd, *J* = 3.0, 1.6 Hz, H-4), 5.05 (1H, qdd, *J* = 6.8, 3.0, 1.6 Hz, H-5), 3.80 (1H, br t, *J* = 6.2 Hz, 8-OH), 3.72 (2H, q, *J* = 6.2 Hz, H-8), 2.43 (2H, tt, *J* = 6.2, 1.6, H-7), 1.36 (3H, d, *J* = 6.8 Hz, H-6). ^{13}C NMR (100 MHz, acetone-d_6) δ: 174.2 (C-2), 152.4 (C-4), 131.3 (C-3), 78.3 (C-5), 60.2 (C-8), 29.5 (C-7), 19.3 (C-6).

Dihydroaspyrone (**10**): Colorless oil (MeOH), $[\alpha]_D^{25}$ +20.7 (*c* 0.35, MeOH). Positive ion ESIMS *m*/*z*: 187 $[M + H]^+$, 209 $[M + Na]^+$; Negative ion ESIMS *m*/*z*: 185 $[M - H]^-$. CD Δε (nm): 0 (192.0), −1.15 (211.5), 0 (219.5), +1.61 (231.0), 0 (245.5), −1.13 (260.0), 0 (300.0). ^{1}H NMR (400 MHz, $CDCl_3$) δ: 6.64 (1H, d, *J* = 3.2 Hz, H-4), 4.38 (1H, m, H-6), 4.15 (1H, dd, *J* = 7.9, 2.8 Hz, H-5), 3.97 (1H, m, H-9), 3.42 (1H, br s, O<u>H</u>), 3.34 (1H, br s, O<u>H</u>), 2.40 (2H, m, H-8), 1.42 (3H, d, *J* = 6.4 Hz, H-7), 1.20 (3H, d, *J* = 6.3 Hz, H-10). ^{13}C NMR (100 MHz, CDCl3) δ: 165.6 (C-2), 145.0 (C-4), 128.9 (C-3), 79.6 (C-6), 67.5 (C-5), 67.0 (C-9), 39.5 (C-8), 23.3 (C-10), 18.2 (C-7).

Aspinotriol A (**12**): Colorless oil (MeOH), $[\alpha]_D^{25}$ −20.9 (*c* 0.9, MeOH). Positive ion ESIMS *m*/*z*: 195 $[M + Na]^+$, 211 $[M + K]^+$; Negative ion ESIMS *m*/*z*: 171 $[M - H]^-$. ^{1}H NMR (400 MHz, CD_3OD) δ: 6.13 (1H, d, *J* = 15.9 Hz, H-4), 5.93 (1H, dd, *J* = 15.9, 6.2 Hz, H-3), 5.56 (1H, d, *J* = 8.7 Hz, H-6), 4.72 (1H, dq, *J* = 8.7, 6.3 Hz, H-7), 4.34–4.26 (1H, m, H-2), 4.29 (2H, s, H-9), 1.26 (3H, d, *J* = 6.4 Hz, H-1), 1.25 (3H, d, *J* = 6.3 Hz, H-8). ^{13}C NMR (100 MHz, CD_3OD) δ: 138.8 (C-3), 137.7 (C-5), 134.9 (C-6), 131.7 (C-4), 69.4 (C-2), 64.6 (C-7), 57.5 (C-9), 23.9 (C-1), 23.7 (C-8).

Aspinotriol B (**13**): Colorless oil (MeOH), $[\alpha]_D^{25}$ +3.8 (*c* 0.7, MeOH). Positive ion ESIMS *m*/*z*: 195 $[M + Na]^+$, 211 $[M + K]^+$; Negative ion ESIMS *m*/*z*: 171 $[M - H]^-$. ^{1}H NMR (400 MHz, CD_3OD) δ: 6.13 (1H, d, *J* = 15.9 Hz, H-4), 5.93 (1H, dd, *J* = 15.9, 6.3 Hz, H-3), 5.56 (1H, d, *J* = 8.7 Hz, H-6), 4.72 (1H, dq, *J* = 8.7, 6.3 Hz, H-7), 4.34–4.26 (1H, m, H-2), 4.29 (2H, s, H-9), 1.26 (3H, d, *J* = 6.4 Hz, H-1), 1.25 (3H, d, *J* = 6.3 Hz, H-8). ^{13}C NMR (100 MHz, CD_3OD) δ: 138.9 (C-3), 137.7 (C-5), 135.0 (C-6), 131.7 (C-4), 69.4 (C-2), 64.7 (C-7), 57.5 (C-9), 23.9 (C-1), 23.7 (C-8).

Chaetoquadrin F (**14**): White crystalline powder (MeOH), m.p. 137−140 °C, $[\alpha]_D^{25}$ +32.8 (*c* 1.0, MeOH). Positive ion ESIMS *m*/*z*: 185 $[M + H]^+$, 207 $[M + Na]^+$; Negative ion ESIMS *m*/*z*: 183 $[M - H]^-$. ^{1}H NMR (400 MHz, CD_3OD) δ: 6.06 (1H, s, H-5), 4.10 (1H, m, H-8), 2.58 (1H, dd, *J* = 14.5, 5.2 Hz, Ha-7), 2.52 (1H, dd, *J* = 14.5, 7.6 Hz, Hb-7), 1.85 (3H, s, 3-CH3), 1.22 (3H, d, *J* = 6.4 Hz, H-9). ^{13}C NMR (100 MHz, CD_3OD) δ: 169.1 (C-2), 167.7 (C-4), 162.0 (C-6), 102.8 (C-5), 99.2 (C-3), 66.2 (C-8), 43.9 (C-7), 23.4 (C-9), 8.3 (3-<u>C</u>H3).

Table S1. 400 MHz ^{1}H NMR and 100 MHz ^{13}C NMR data of **1** in $CDCl_3$ [a].

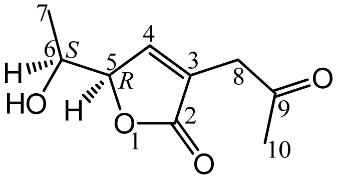

1

Position	δ_C [b]	δ_H (*J* in Hz) [b]	COSY [c]	HMBC [d]
2	173.5 s	-	-	-
3	128.2 s	-	-	-
4	149.4 d	7.43 q (1.4)	H-5, H-8	C-2,3,5,8
5	85.5 d	4.88 dq (4.7, 1.4)	H-4, H-6, H-8	C-3,4,6,7
6	67.8 d	4.02 qd (6.5, 4.7)	H-5, H-7	C-4,5,7
7	18.9 q	1.28 3H d (6.5)	H-6	C-5,6
8	39.1 t	3.46 2H t (1.4)	H-4, H-5	C-2,3,4,5,6,9
9	203.6 s	-	-	-
10	30.3 q	2.24 3H s		C-3,8,9

[a] Signals assignments were based on the results of ^{1}H–^{1}H COSY, HMQC, and HMBC experiments. [b] Chemical shift values (δ_H and δ_C) were recorded using the solvent signals ($CDCl_3$: δ_H 7.26/δ_C 77.1) as references, respectively. [c] The numbers in each line of this column indicate the protons that correlated with the proton in the corresponding line in ^{1}H–^{1}H COSY. [d] The numbers in each line of this column indicate the carbons that showed HMBC correlations with the proton in the corresponding line in the HMBC experiments optimized for the 8.3 Hz of long-range J_{CH} value.

Table S2. 400 MHz ^{1}H NMR and 100 MHz ^{13}C NMR data of **2** in $CDCl_3$ [a].

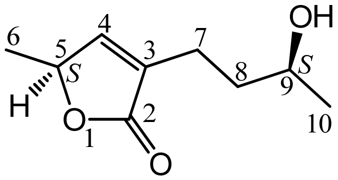

2

Position	δ_C [b]	δ_H (*J* in Hz) [b]	COSY [c]	HMBC [d]
2	174.3 s	-	-	-
3	134.0 s	-	-	-
4	149.8 d	7.04 q (1.6)	H-5, H-7	C-2,3,5,7
5	77.8 d	5.01 qq (6.9,1.6)	H-4, H-6, H-7	C-2,3,4,6
6	19.3 q	1.41 3H d (6.9)	H-5	C-4,5
7	21.6 t	2.28–2.57 2H m	H-4, H-5, H-8	C-2,3,4,8,9
8	37.2 t	1.60–1.78 2H m	H-7, H-9	C-3,7,9
9	67.0 d	3.80 sext (6.4)	H-8, H-10	C-7
10	23.6 q	1.21 3H d (6.4)	H-9	C-8,9

[a] Signals assignments were based on the results of ^{1}H–^{1}H COSY, HMQC, and HMBC experiments. [b] Chemical shift values (δ_H and δ_C) were recorded using the solvent signals ($CDCl_3$: δ_H 7.26/δ_C 77.1) as references, respectively. [c] The numbers in each line of this column indicate the protons that correlated with the proton in the corresponding line in ^{1}H–^{1}H COSY. [d] The numbers in each line of this column indicate the carbons that showed HMBC correlations with the proton in the corresponding line in the HMBC experiments optimized for the 8.3 Hz of long-range J_{CH} value.

Table S3. 400 MHz ^{1}H NMR and 100 MHz ^{13}C NMR data of **3** in CD_3OD [a].

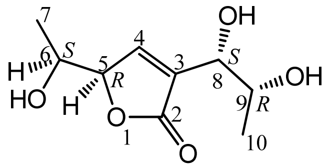

3

Position	δ_C [b]	δ_H (J in Hz) [b]	COSY [c]	HMBC [d]
2	174.5 s	-	-	-
3	136.9 s	-	-	-
4	150.3 d	7.54 dd (3.0, 1.5)	H-5, H-8	C-2,3,5,8
5	87.1 d	4.96~4.92 m	H-4, H-6	C-2,3,4,6,7
6	68.5 d	3.95 qd (6.5, 4.6)	H-5, H-7	C-4,5,7
7	19.0 q	1.25 3H d (6.5)	H-6	C-5,6
8	71.7 d	4.36 dt (4.6, 1.5)	H-4, H-5, H-9	C-2,3,4,9,10
9	69.8 d	4.03 qd (6.4, 4.6)	H-8, H-10	C-3
10	17.7 q	1.12 3H d (6.4)	H-9	C-8

[a] Signals assignments were based on the results of ^{1}H–^{1}H COSY, HMQC, and HMBC experiments. [b] Chemical shift values (δ_H and δ_C) were recorded using the solvent signals (CD_3OD: δ_H 3.31/δ_C 49.0) as references, respectively. [c] The numbers in each line of this column indicate the protons that correlated with the proton in the corresponding line in ^{1}H–^{1}H COSY. [d] The numbers in each line of this column indicate the carbons that showed HMBC correlations with the proton in the corresponding line in the HMBC experiments optimized for the 8.3 Hz of long-range J_{CH} value.

Table S4. 400 MHz ^{1}H NMR and 100 MHz ^{13}C NMR data of **4** in CD_3OD [a].

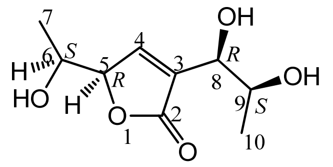

4

Position	δ_C [b]	δ_H (J in Hz) [b]	COSY [c]	HMBC [d]
2	174.5 s	-	-	-
3	137.0 s	-	-	-
4	150.5 d	7.56 dd (2.8, 1.6)	H-5, H-8	C-2,3,5,8
5	87.0 d	4.92 ddd (5.0, 2.8, 1.6)	H-4, H-5, H-6	C-2,3,4,6,7
6	68.6 d	3.92 qd (6.4, 5.0)	H-5, H-7	C-4,5,7
7	19.1 q	1.26 3H d (6.4)	H-6	C-5,6
8	71.7 d	4.34 br d (4.9)	H-4, H-5, H-9	C-2,3,4,9,10
9	70.1 d	3.99 qd (6.4, 4.9)	H-8, H-10	C-3
10	17.9 q	1.12 3H d (6.4)	H-9	C-8

[a] Signals assignments were based on the results of ^{1}H–^{1}H COSY, HMQC, and HMBC experiments. [b] Chemical shift values (δ_H and δ_C) were recorded using the solvent signals (CD_3OD: δ_H 3.31/δ_C 49.0) as references, respectively. [c] The numbers in each line of this column indicate the protons that correlated with the proton in the corresponding line in ^{1}H–^{1}H COSY. [d] The numbers in each line of this column indicate the carbons that showed HMBC correlations with the proton in the corresponding line in the HMBC experiments optimized for the 8.3 Hz of long-range J_{CH} value.

Table S5. 400 MHz ^{1}H NMR and 100 MHz ^{13}C NMR data of **5** in CD_3OD [a].

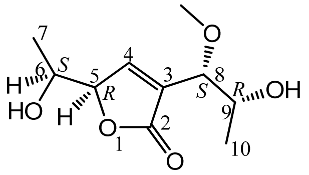

5

Position	δ_C [b]	δ_H (*J* in Hz) [b]	COSY [c]	HMBC [d]
2	174.6 s	-	-	-
3	133.9 s	-	-	-
4	152.0 d	7.55 dd (1.5, 0.9)	H-5, H-8	C-2,3,5,8
5	87.4 d	4.98 dt (4.5, 1.5)	H-4, H-6, H-8	C-2,3,4,6,7
6	68.5 d	3.97 qd (6.4, 4.5)	H-5, H-7	C-4,5,7
7	19.0 q	1.26 3H d (6.4)	H-6	C-5,6
8	81.6 d	3.96 br d (4.7)	H-4, H-5, H-9	C-2,3,4,9,10,11
9	69.0 d	4.04 qd (6.4, 4.7)	H-8, H-10	C-3,8,10
10	18.2 q	1.12 3H d (6.4)	H-9	C-8,9
OCH_3	58.1 q	3.35 3H s	-	C-8

[a] Signals assignments were based on the results of ^{1}H–^{1}H COSY, HMQC, and HMBC experiments. [b] Chemical shift values (δ_H and δ_C) were recorded using the solvent signals (CD_3OD: δ_H 3.31/δ_C 49.0) as references, respectively. [c] The numbers in each line of this column indicate the protons that correlated with the proton in the corresponding line in ^{1}H–^{1}H COSY. [d] The numbers in each line of this column indicate the carbons that showed HMBC correlations with the proton in the corresponding line in the HMBC experiments optimized for the 8.3 Hz of long-range J_{CH} value.

Table S6. 400 MHz ^{1}H NMR and 100 MHz ^{13}C NMR data of **6**/**7** in CD_3OD [a].

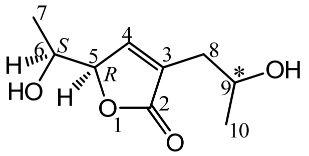

6/**7**: 9*R**/9*S**

Position	δ_C [b]	δ_H (*J* in Hz) [b]	COSY [c]	HMBC [d]
2	176.25 s/176.18 s	-	-	-
3	133.08 s/133.04 s	-	-	-
4	150.49 d/150.03 d	7.41 dd (2.8, 1.6)/7.32 dd (2.8, 1.6)	H-5, H-8	C-3,5,8
5	86.85 d/86.82 d	4.87 dd (4.4, 2.8, 1.6)	H-4 ,H-6, H-8	C-3,4,6
6	68.57 d/68.33 d	3.92 qd (6.4, 4.4)	H-5, H-7	C-4,5,7
7	19.15 q/19.02 q	1.25 3H d (6.4)	H-6	C-5,6
8	35.72 t/35.67 t	2.41 2H br d (6.2)	H-4, H-5, H-9	C-2,3,4,9,10
9	66.49 d/66.47 d	4.02 sext (6.2)	H-8, H-10	C-3,8,10
10	23.26 q	1.19 3H d (6.2)	H-9	C-8,9

[a] Signals assignments were based on the results of ^{1}H–^{1}H COSY, HMQC, and HMBC experiments. [b] Chemical shift values (δ_H and δ_C) were recorded using the solvent signals (CD_3OD: δ_H 3.31/δ_C 49.0) as references, respectively. [c] The numbers in each line of this column indicate the protons that correlated with the proton in the corresponding line in ^{1}H–^{1}H COSY. [d] The numbers in each line of this column indicate the carbons that showed HMBC correlations with the proton in the corresponding line in the HMBC experiments optimized for the 8.3 Hz of long-range J_{CH} value.

Table S7. 400 MHz ^{1}H NMR and 100 MHz ^{13}C NMR data of **9** in CD_3OD [a].

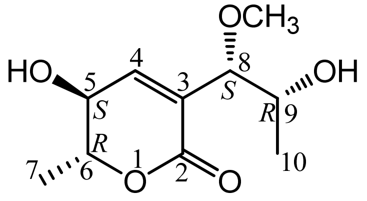

9

Position	δ_C [b]	δ_H (J in Hz) [b]	COSY [c]	HMBC [d]
2	165.7 s	-	-	-
3	129.9 s	-	-	-
4	147.8 d	6.78 t (1.4)	H-5, H-8	C-2, 6, 8
5	68.7 d	4.21 dd (9.4,1.4)	H-4, H-6	C-4, 6, 7
6	80.0 d	4.24 qd (9.4, 5.8)	H-5, H-7	C-4
7	18.20 q	1.44 3H d (5.8)	H-6	C-5, 6
8	83.5 d	4.08 dt (4.9, 1.4)	H-4, H-9	C-2,3,4,9,11
9	70.1 d	3.82 qd (6.4,4.9)	H-8, H-10	C-3, 10
10	18.17 q	1.12 3H d (6.4)	H-9	C-8, 9
OCH_3	58.0 q	3.32 3H s	-	C-8

[a] Signals assignments were based on the results of ^{1}H–^{1}H COSY, HMQC and HMBC experiments. [b] Chemical shift values (δ_H and δ_C) were recorded using the solvent signals (CD_3OD: δ_H 3.31/δ_C 49.0) as references, respectively. [c] The numbers in each line of this column indicate the protons that correlated with the proton in the corresponding line in ^{1}H–^{1}H COSY. [d] The numbers in each line of this column indicate the carbons that showed HMBC correlations with the proton in the corresponding line in the HMBC experiments optimized for the 8.3 Hz of long-range J_{CH} value.

Figure S1. HPLC analysis of **1**, **3**, **4** and **6**/**7** after treatment in a water-saturated EtOAc at the conditions simulated their extraction conditions.

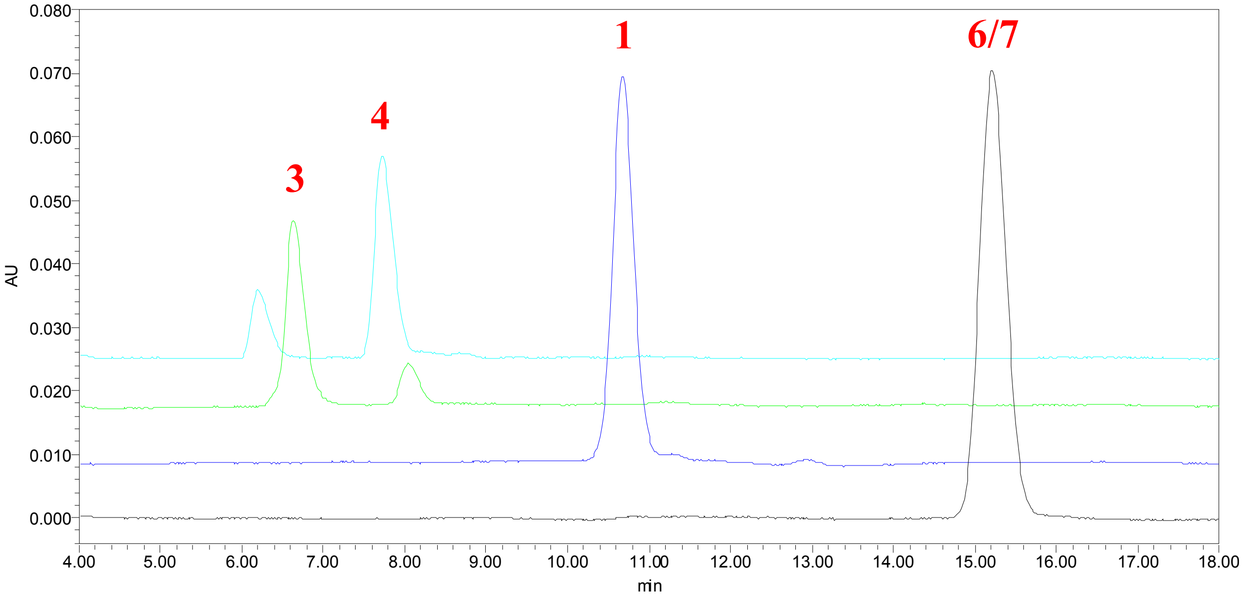

Each 0.3 mg of the crude compound samples was dissolved in 0.2 mL water-saturated EtOAc in a 0.5 mL Eppendorf tube, capped the tube and kept at room temperature for 6 days, and then further treated at 50 °C for 16 h. These treatment conditions simulated the extraction conditions (whole extraction was achieved within 4 days with a total of 12 h evaporating times at the temperature lower than 40 °C). Then, the aqueous EtOAc was removed by blowing inside of the tube with nitrogen gas to dryness. The residue was dissolved in MeOH and then subjected to HPLC analysis. No any one of them was detected in other compound samples by the HPLC analysis, confirming that none of these compounds are artificial product formed from the others in the extraction conditions.

Column: Capcell MGII 4.6 × 250 nm, Mobile phase: 15% MeOH; Fllow rate: 0.8 mL/min; Wave length: 213 nm.

Figure S2. Positive ion ESI-MS spectrum of **1**.

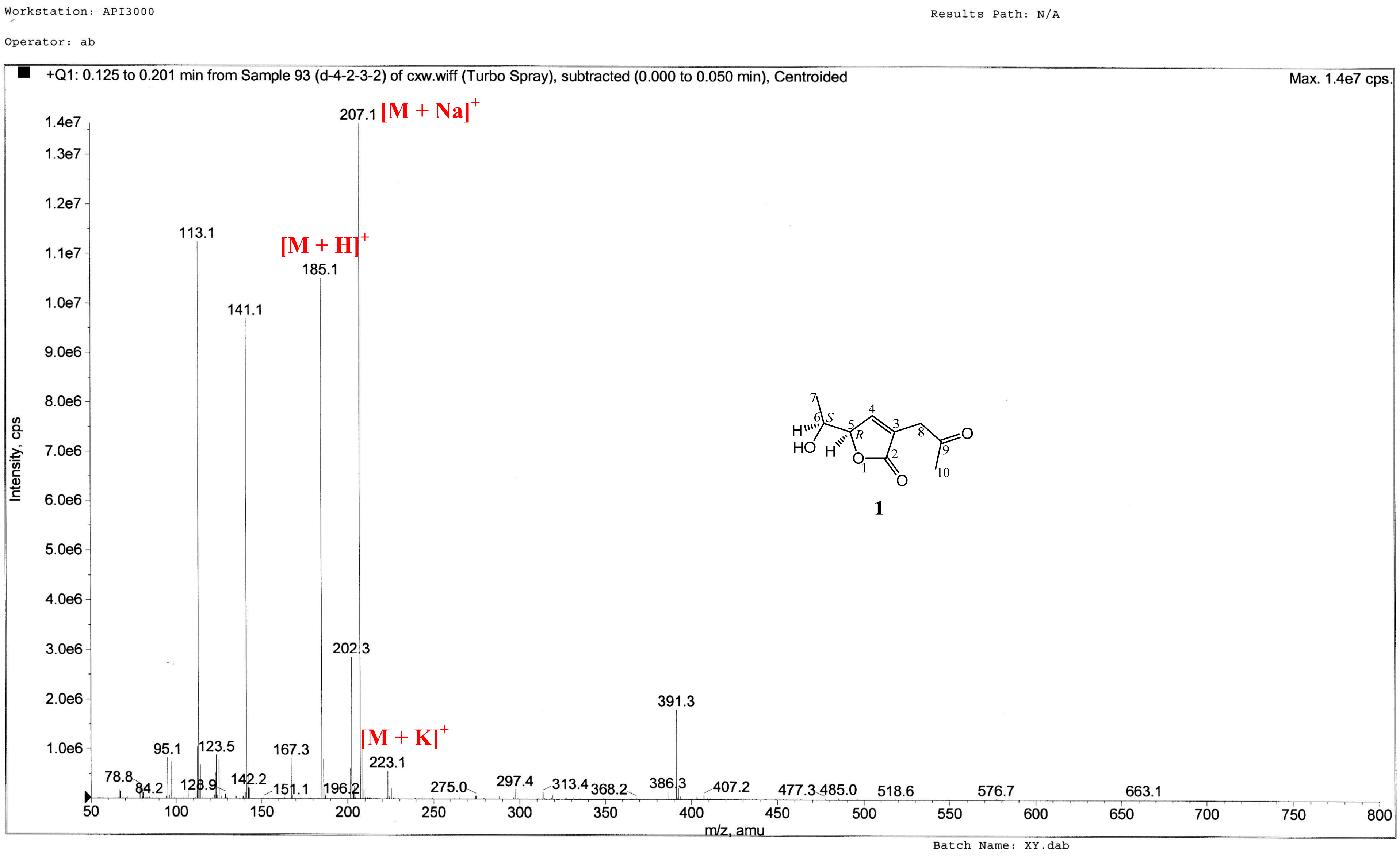

Figure S3. Positive ion HR-ESI-MS spectrum of **1**.

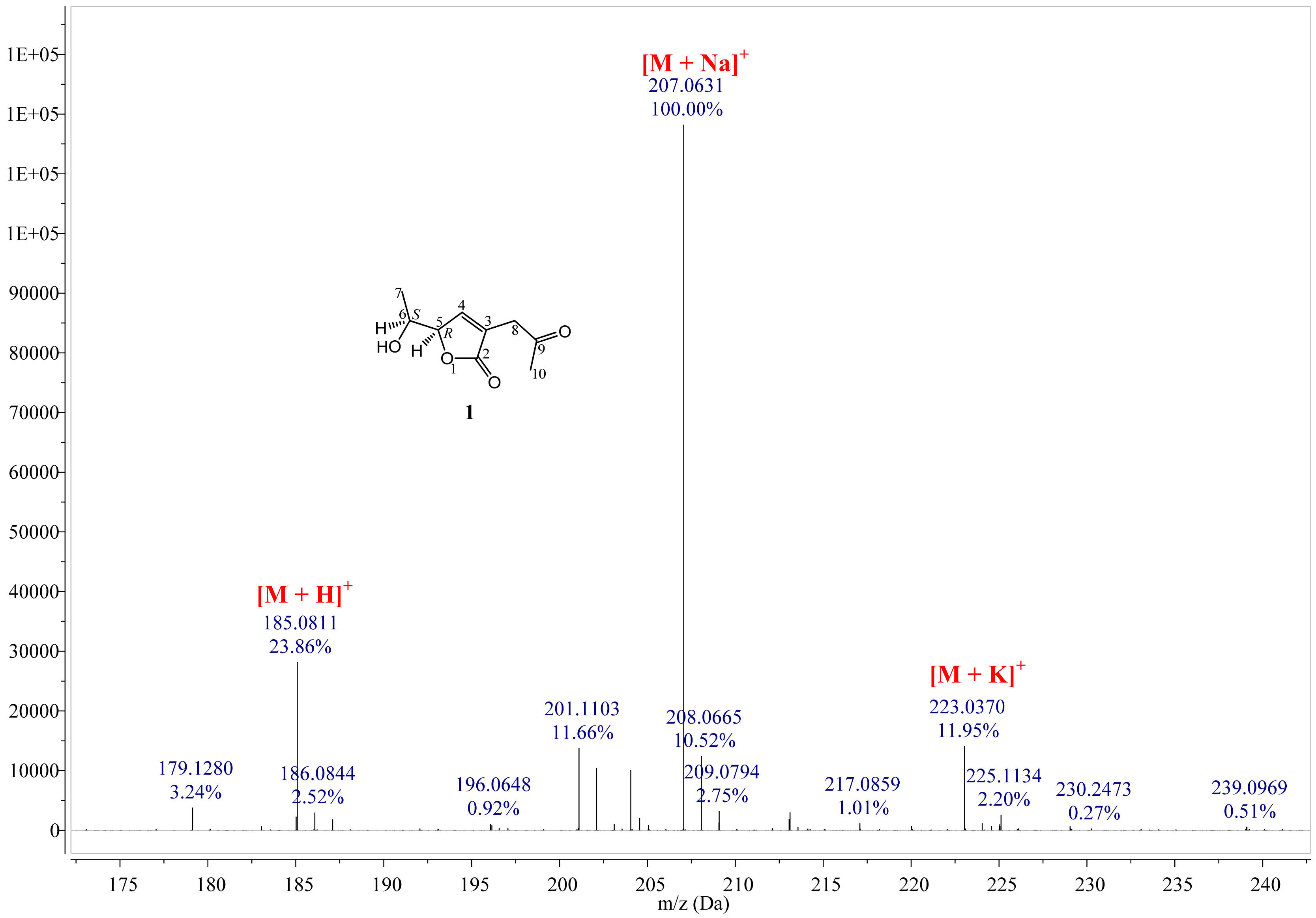

Figure S4. IR spectrum of **1**.

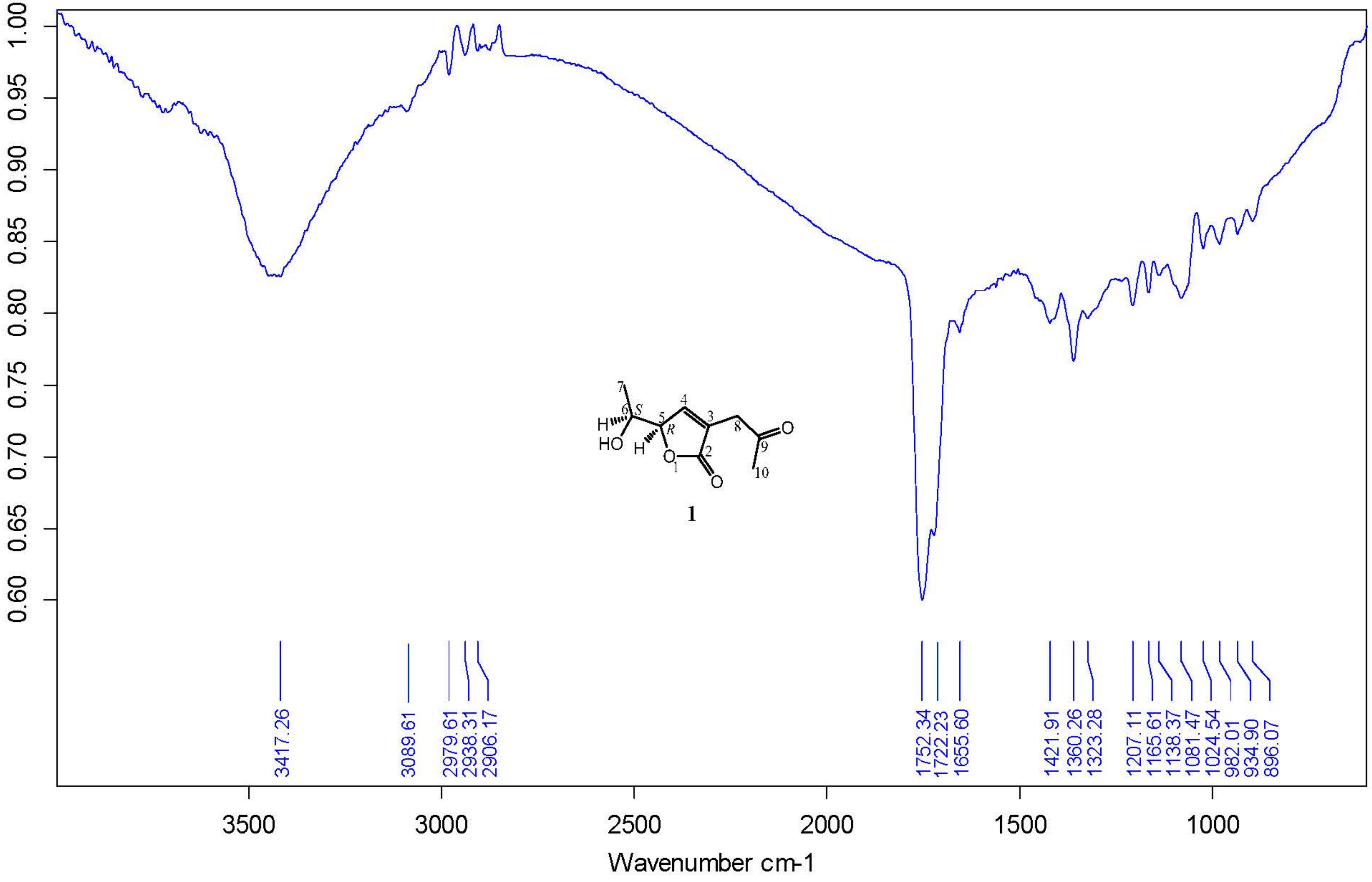

Figure S5. 400 MHz ^{1}H NMR spectrum of **1** in $CDCl_3$.

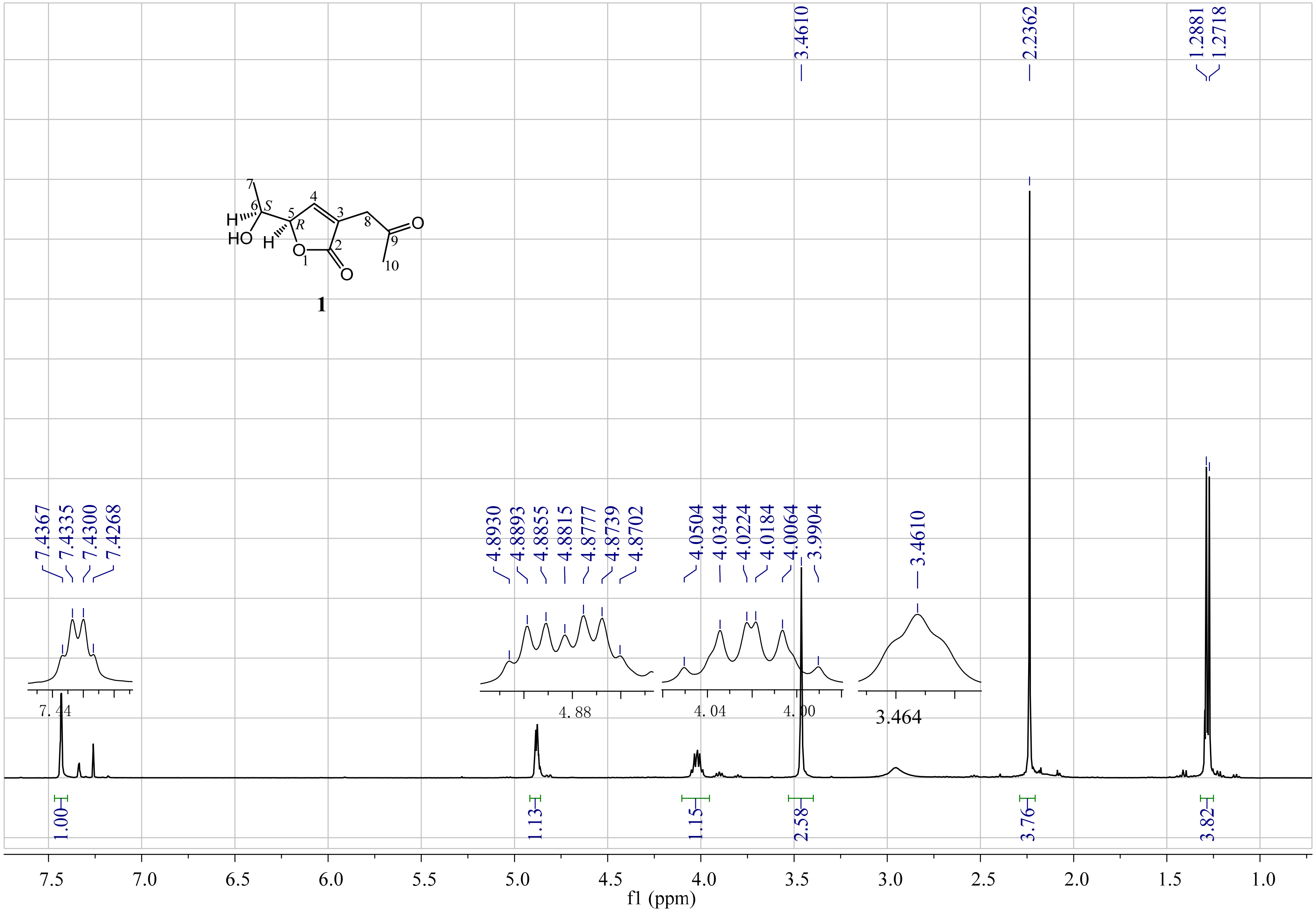

Figure S6. 100 MHz ^{13}C NMR spectrum of **1** in $CDCl_3$.

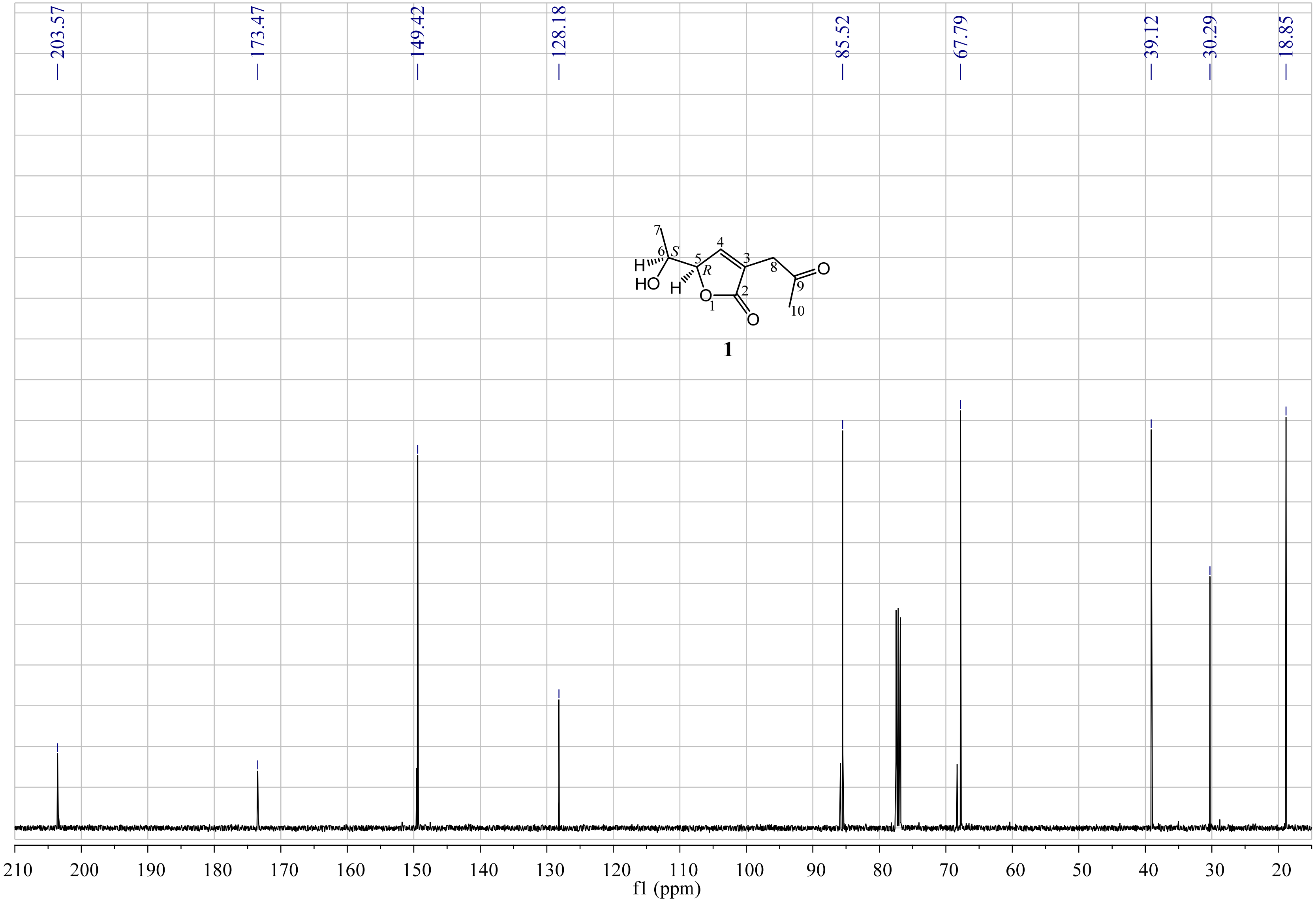

Figure S7. HMQC spectrum of **1** in $CDCl_3$.

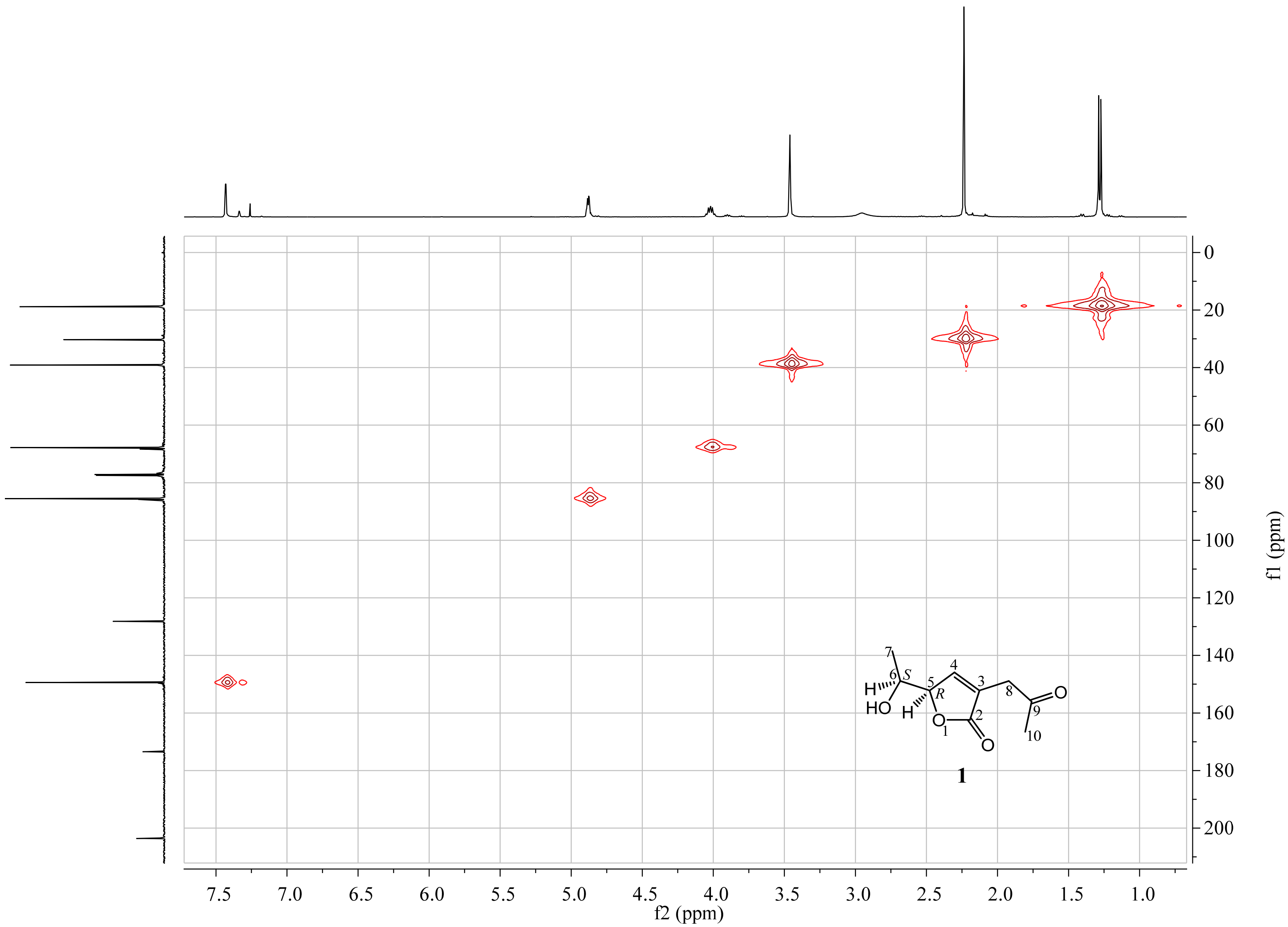

Figure S8. ^{1}H–^{1}H COSY spectrum of **1** in $CDCl_3$.

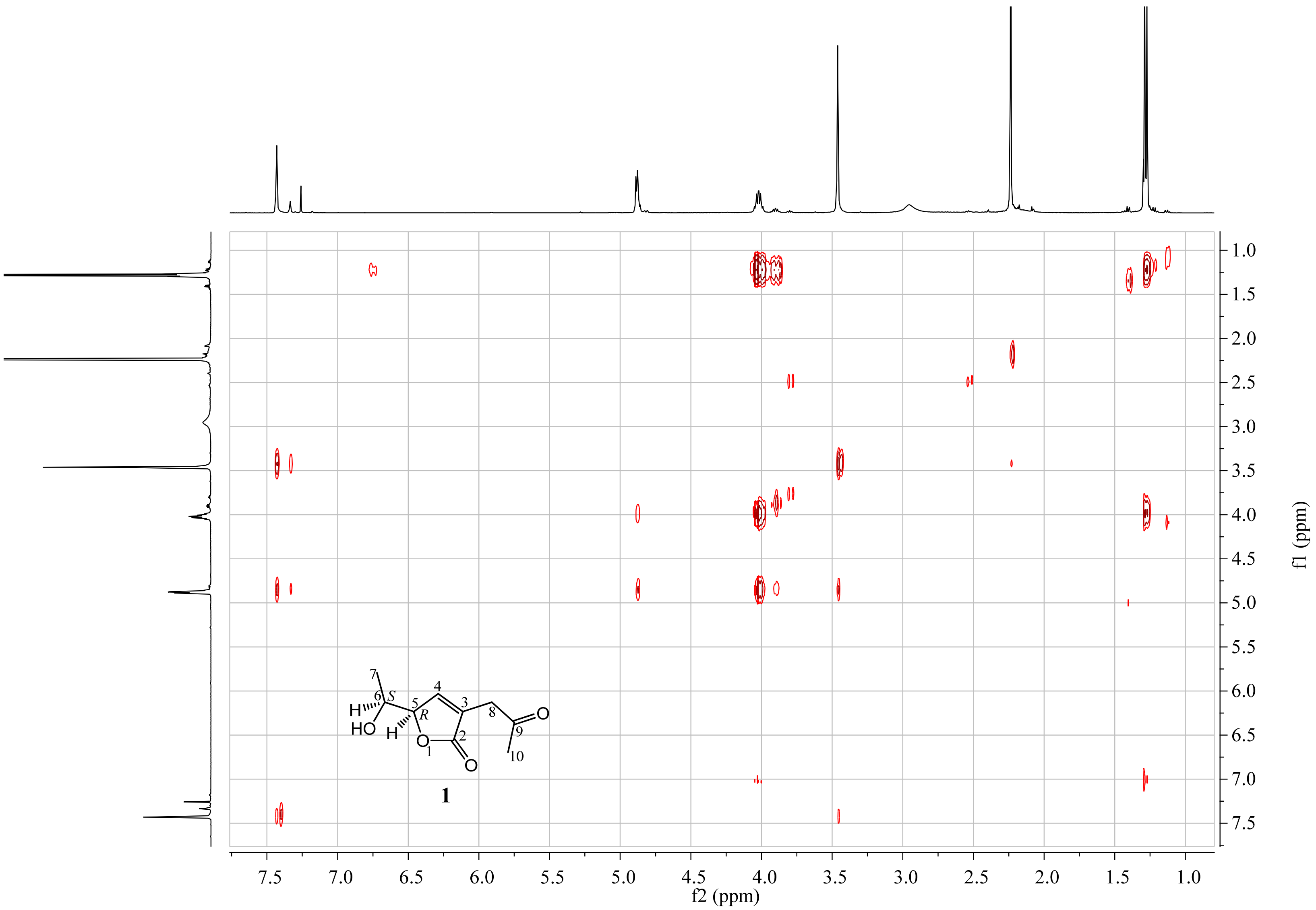

Figure S9. HMBC spectrum of **1** in $CDCl_3$.

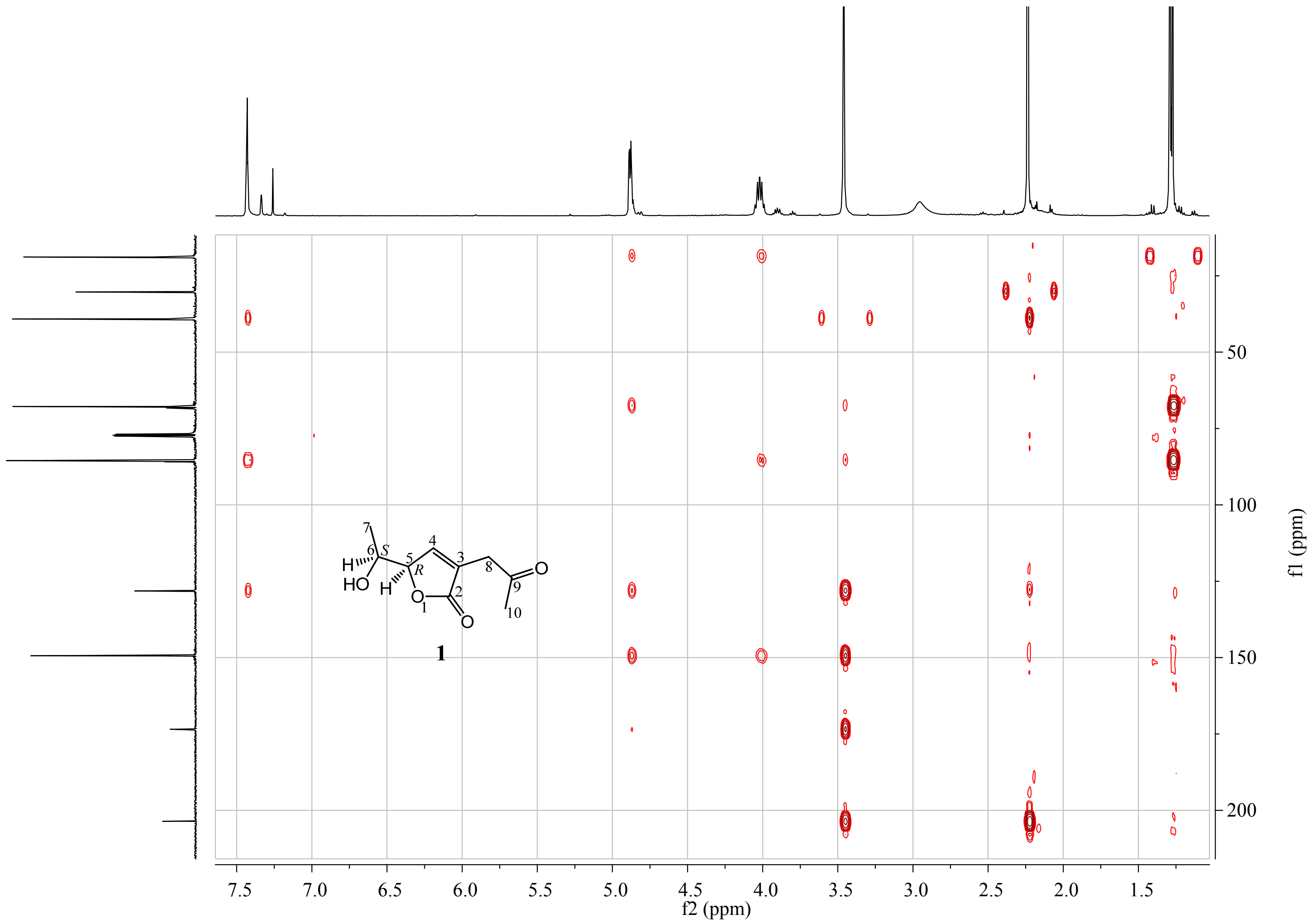

Figure S10. Positive ion ESI-MS spectrum of **2**.

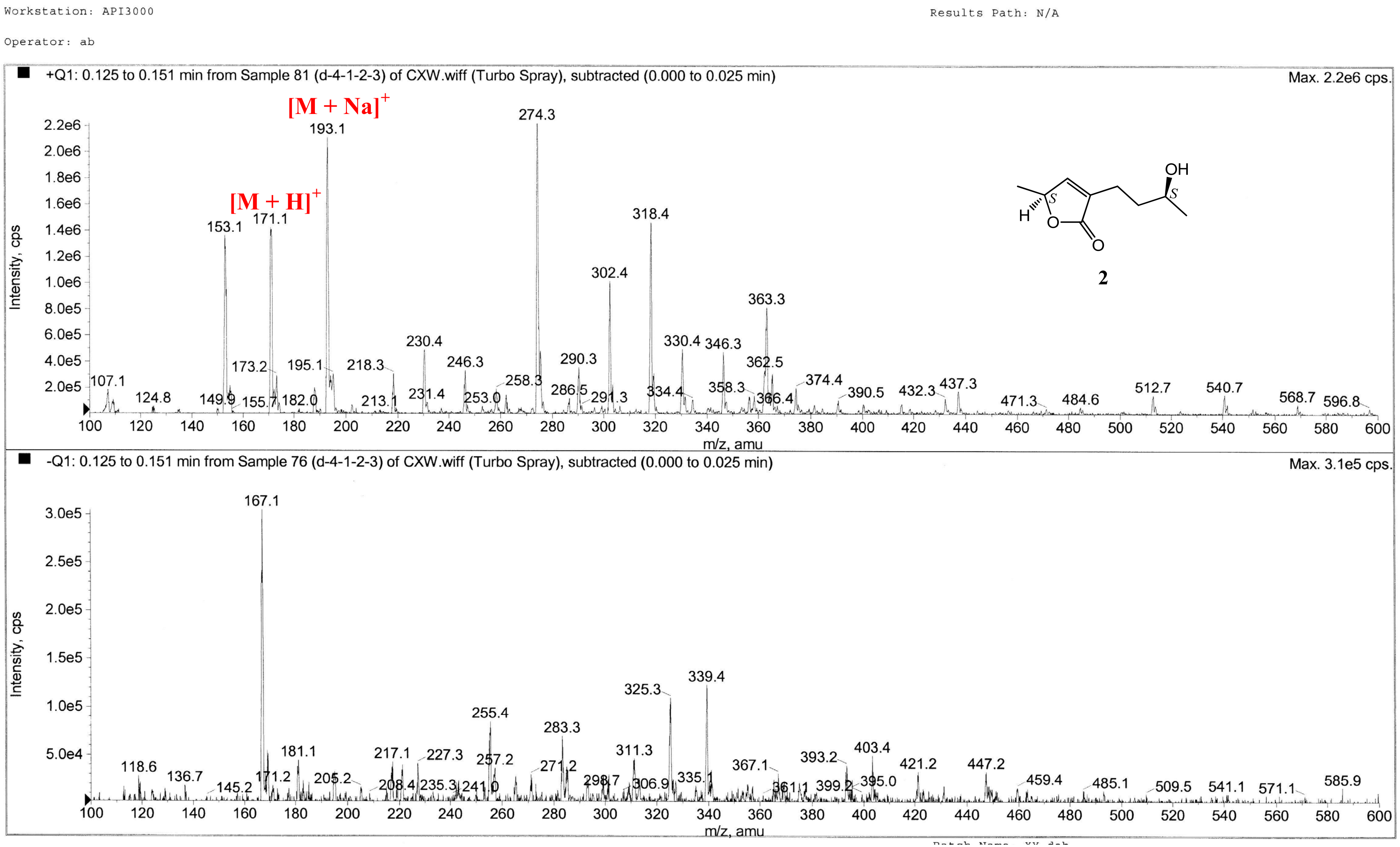

Figure S11. Positive ion HR-ESI-MS spectrum of **2**.

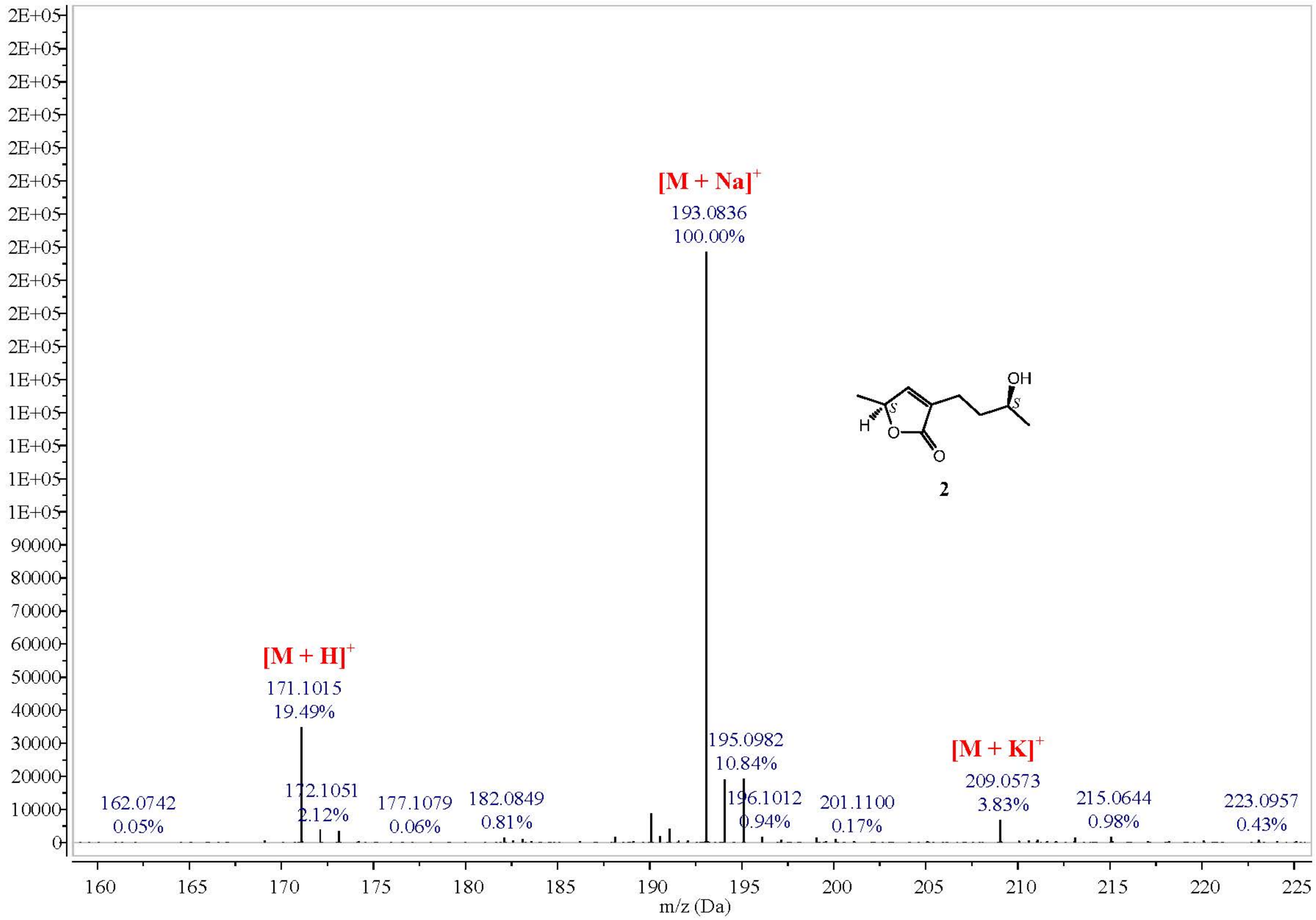

Figure S12. IR spectrum of **2**.

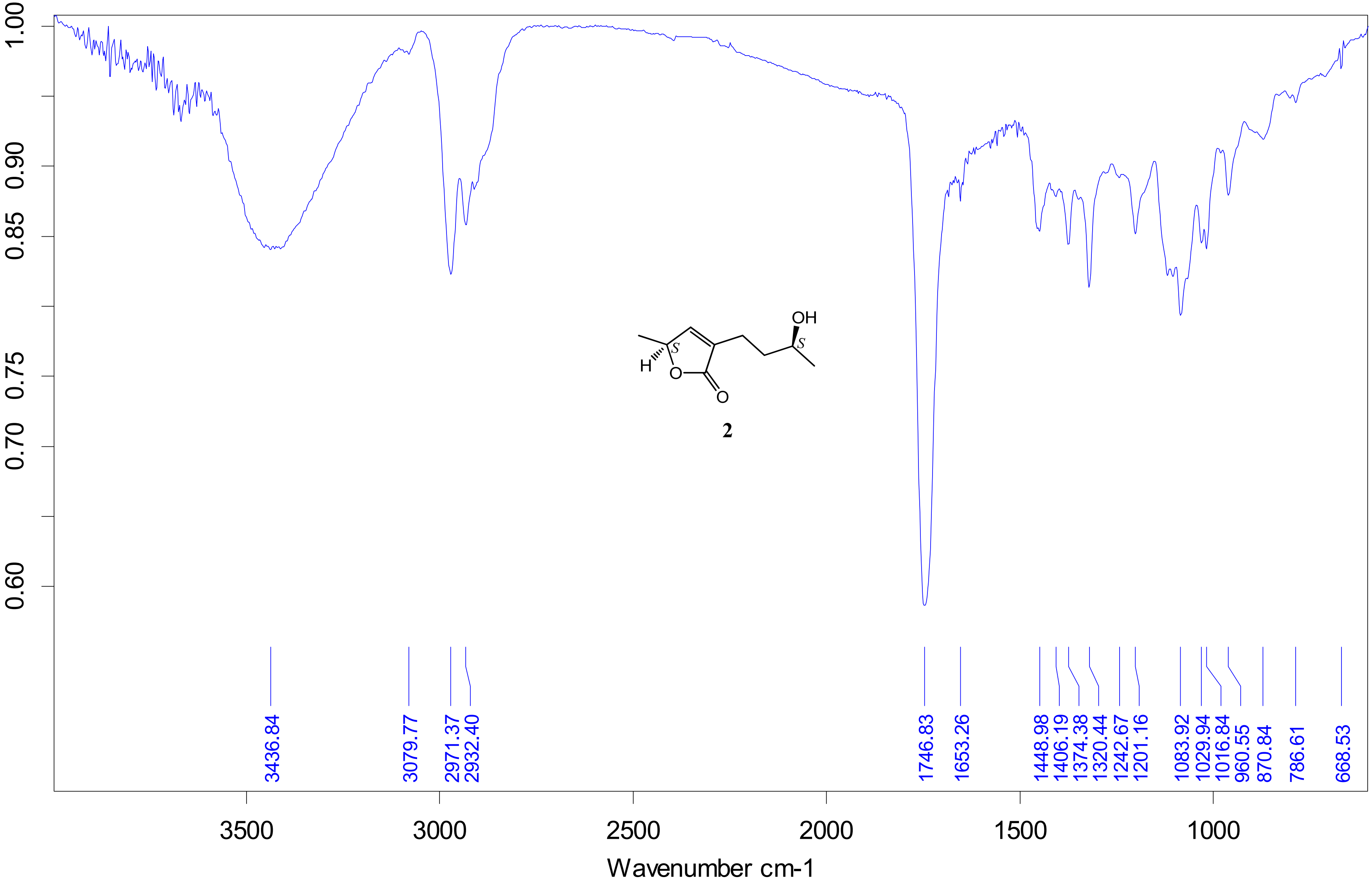

Figure S13. 400 MHz ^{1}H NMR spectrum of **2** in $CDCl_3$.

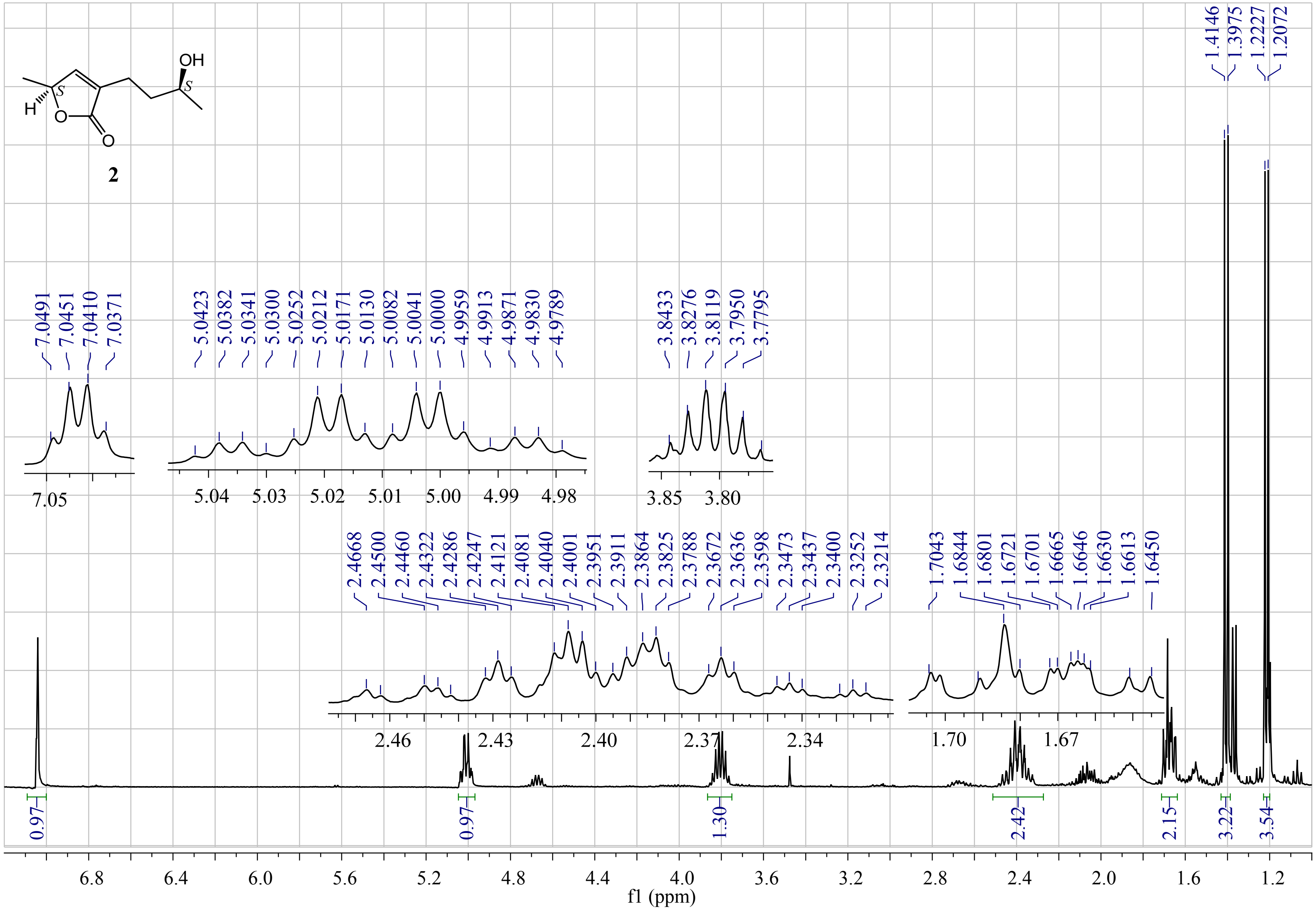

Figure S14. 100 MHz ^{13}C NMR spectrum of **2** in $CDCl_3$.

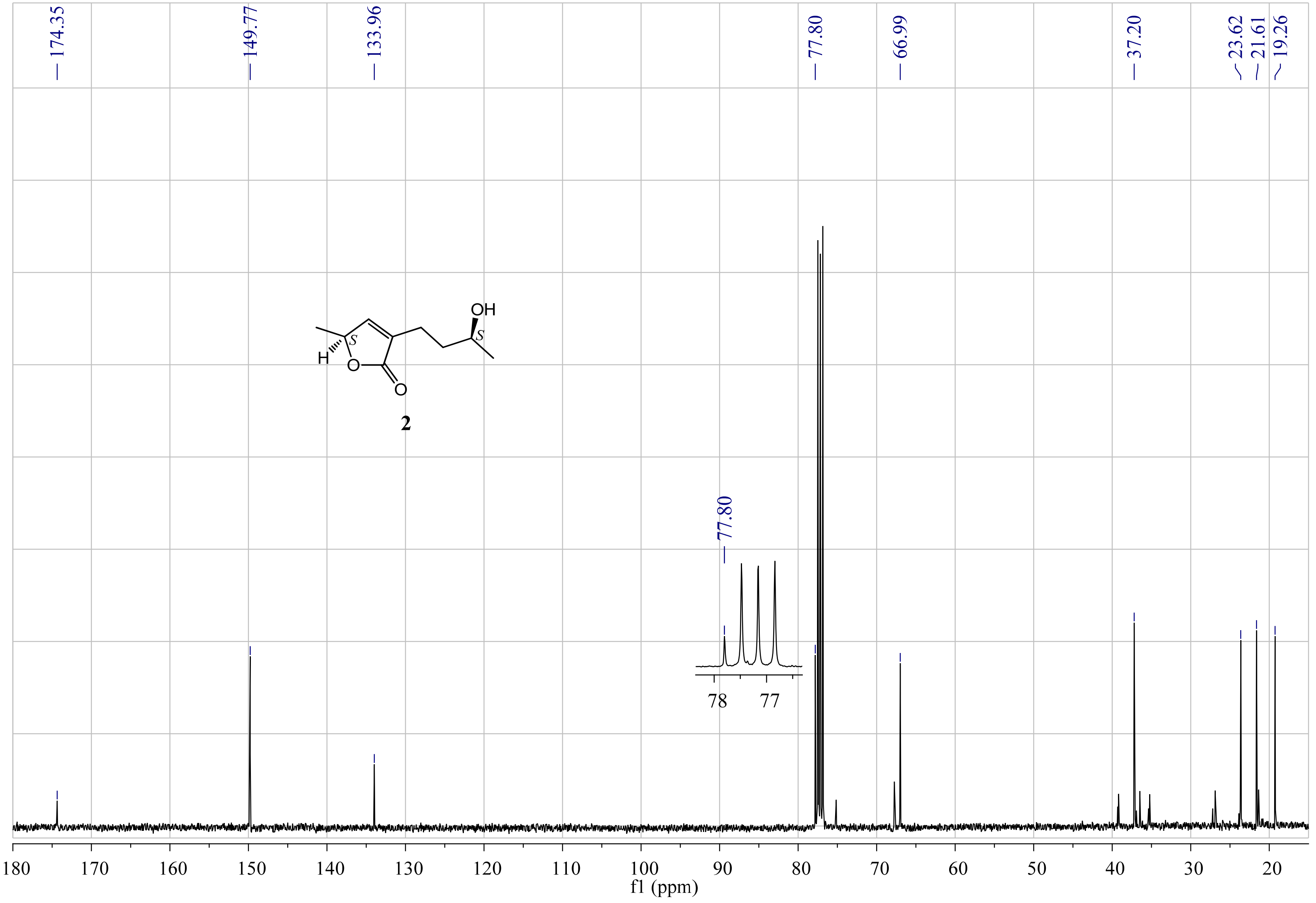

Figure S15. ^{1}H–^{1}H COSY spectrum of **2** in $CDCl_3$.

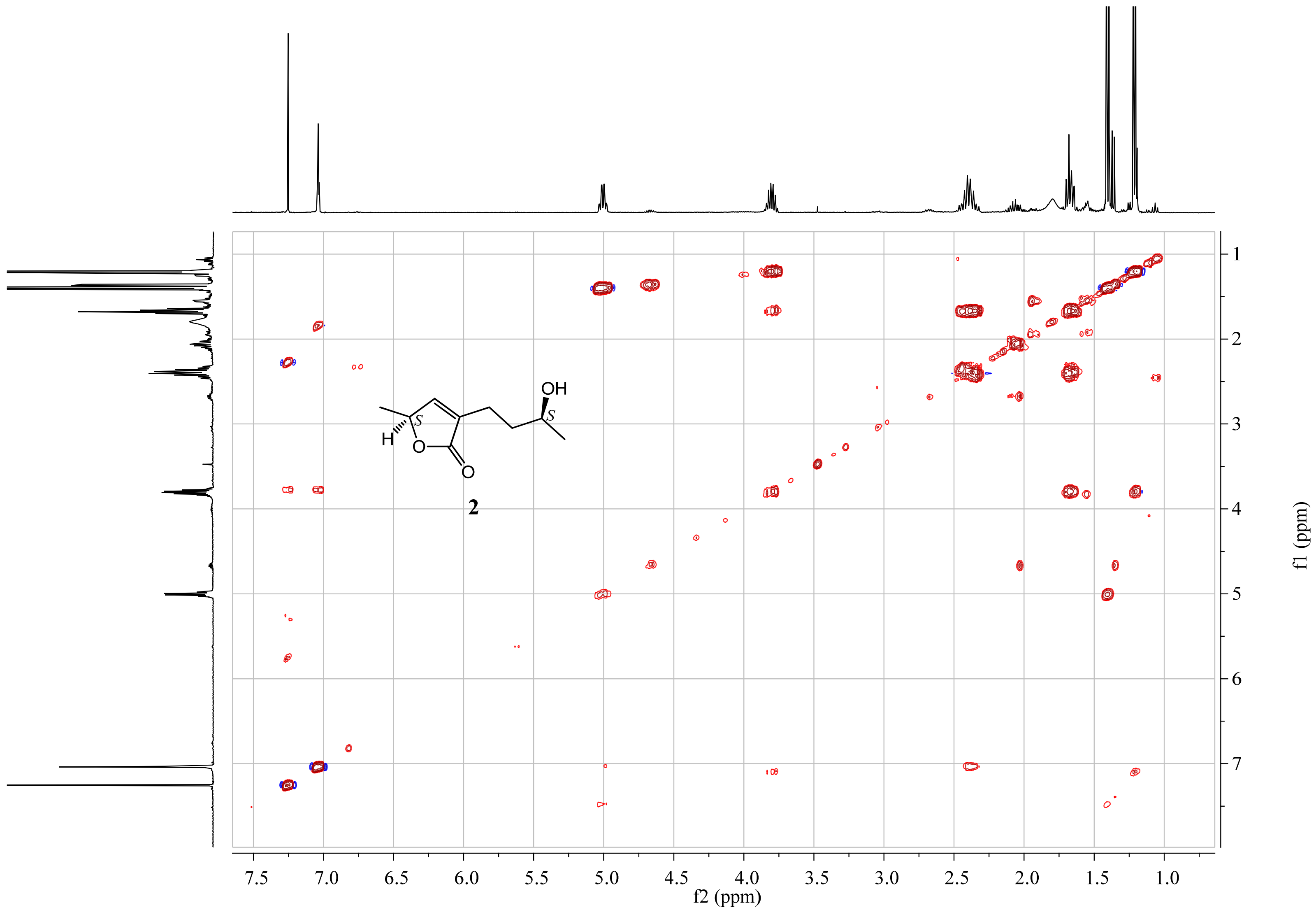

Figure S16. HMQC spectrum of **2** in $CDCl_3$.

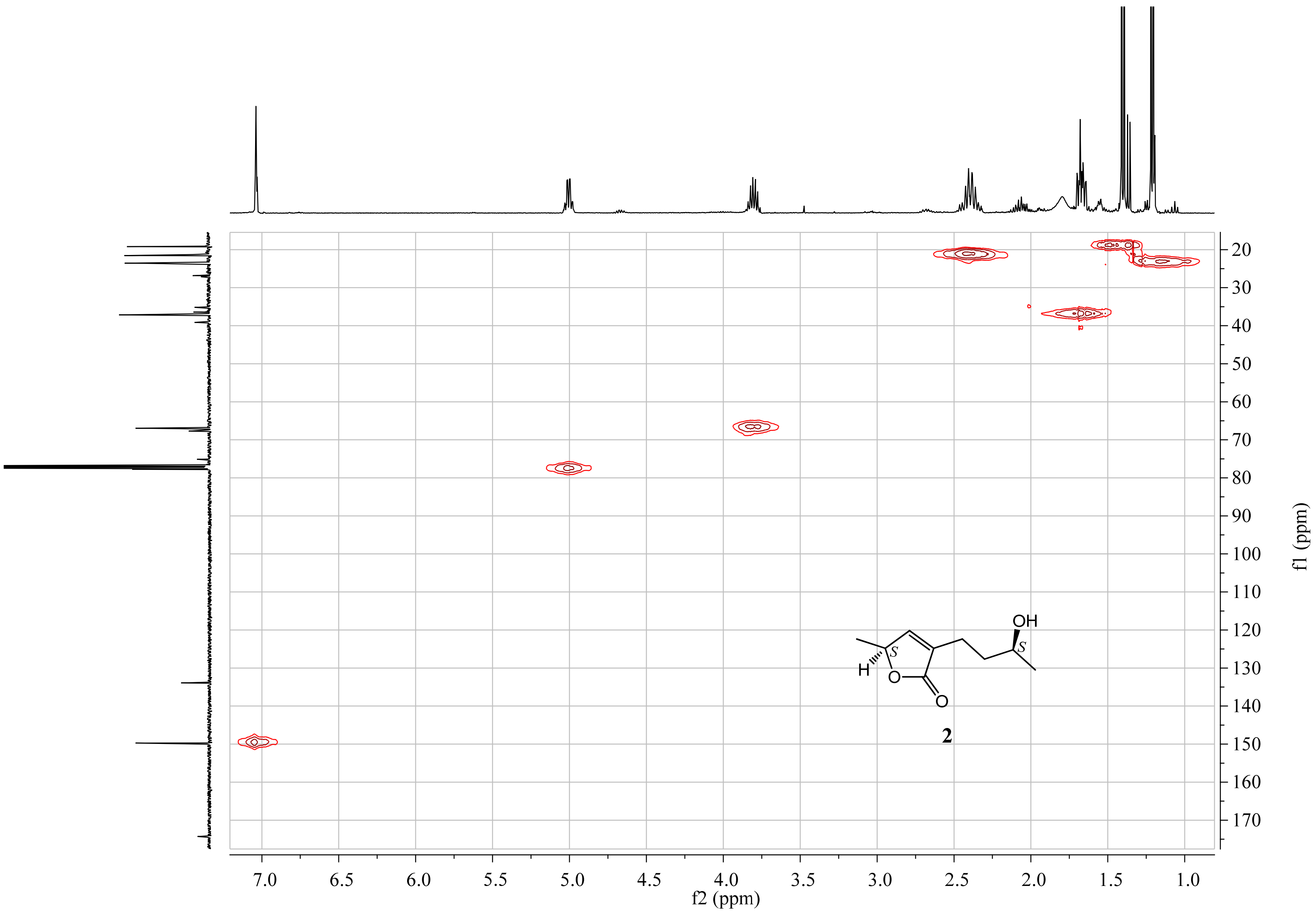

Figure S17. HMBC spectrum of **2** in $CDCl_3$.

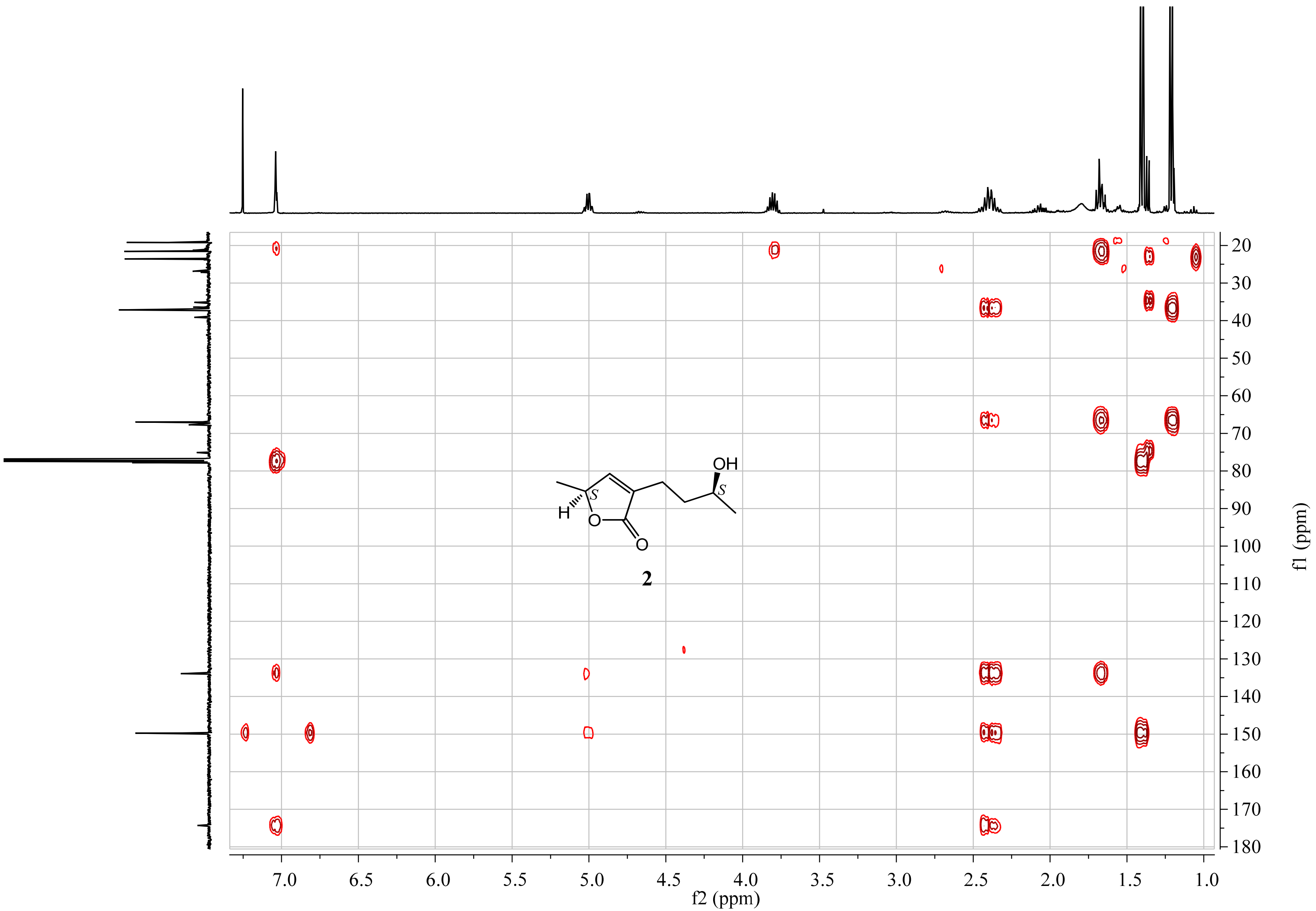

Figure S18. 400 MHz ^{1}H NMR of the (*S*)- and (*R*)-MTPA esters of **2** in pyridine-d_5.

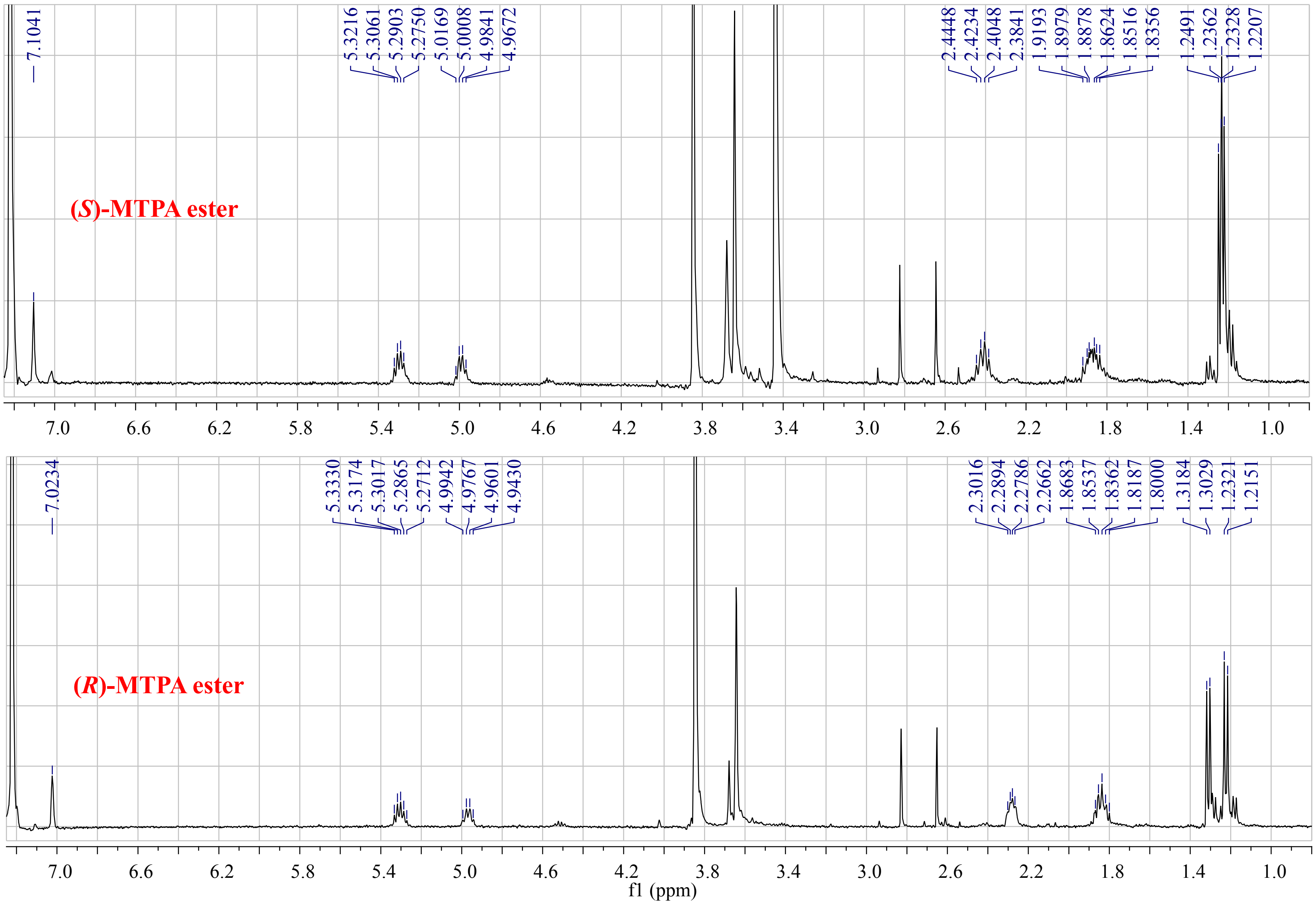

Figure S19. Positive ion ESI-MS spectrum of **3**.

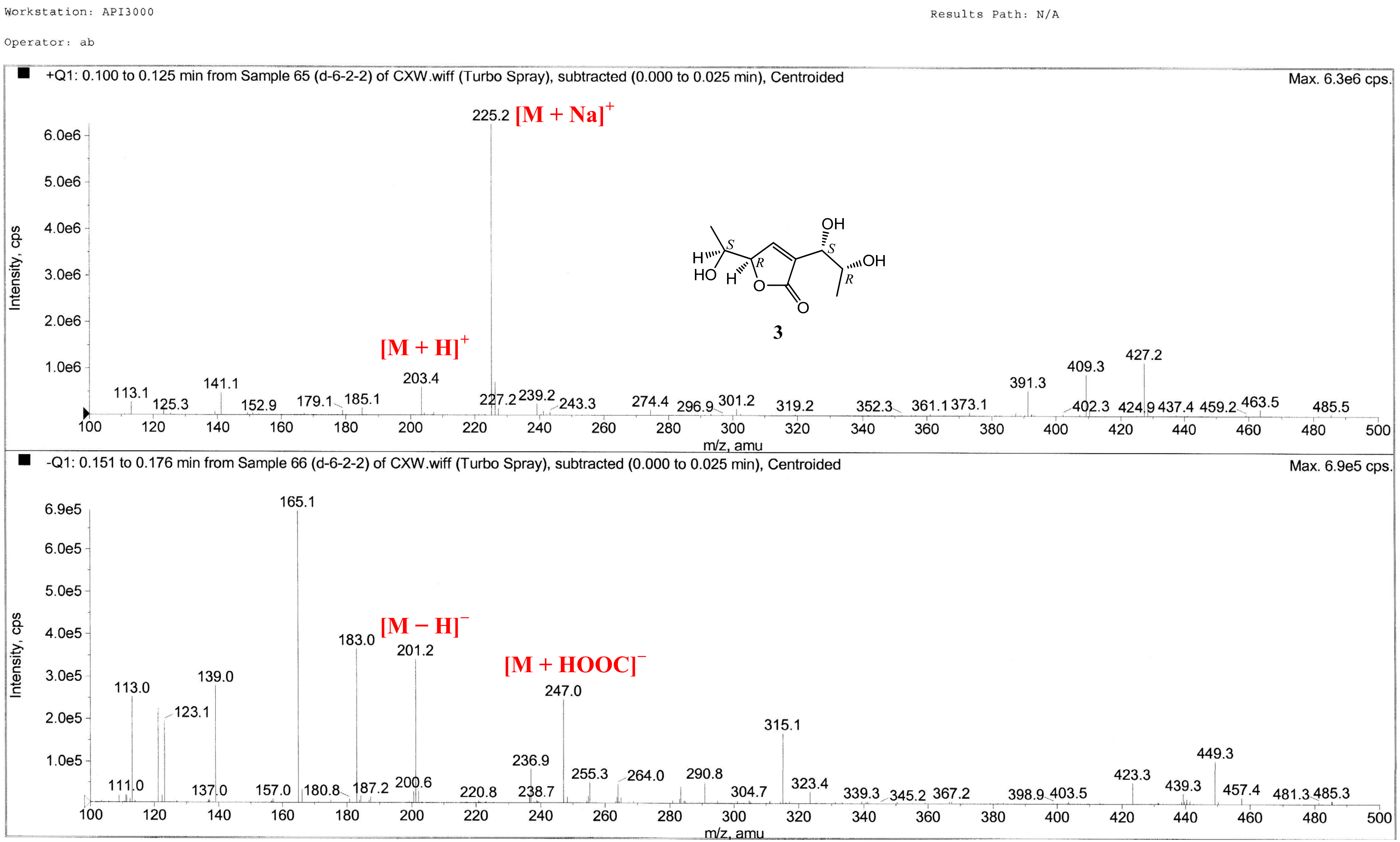

Figure S20. Positive ion HR-ESI-MS spectrum of **3**.

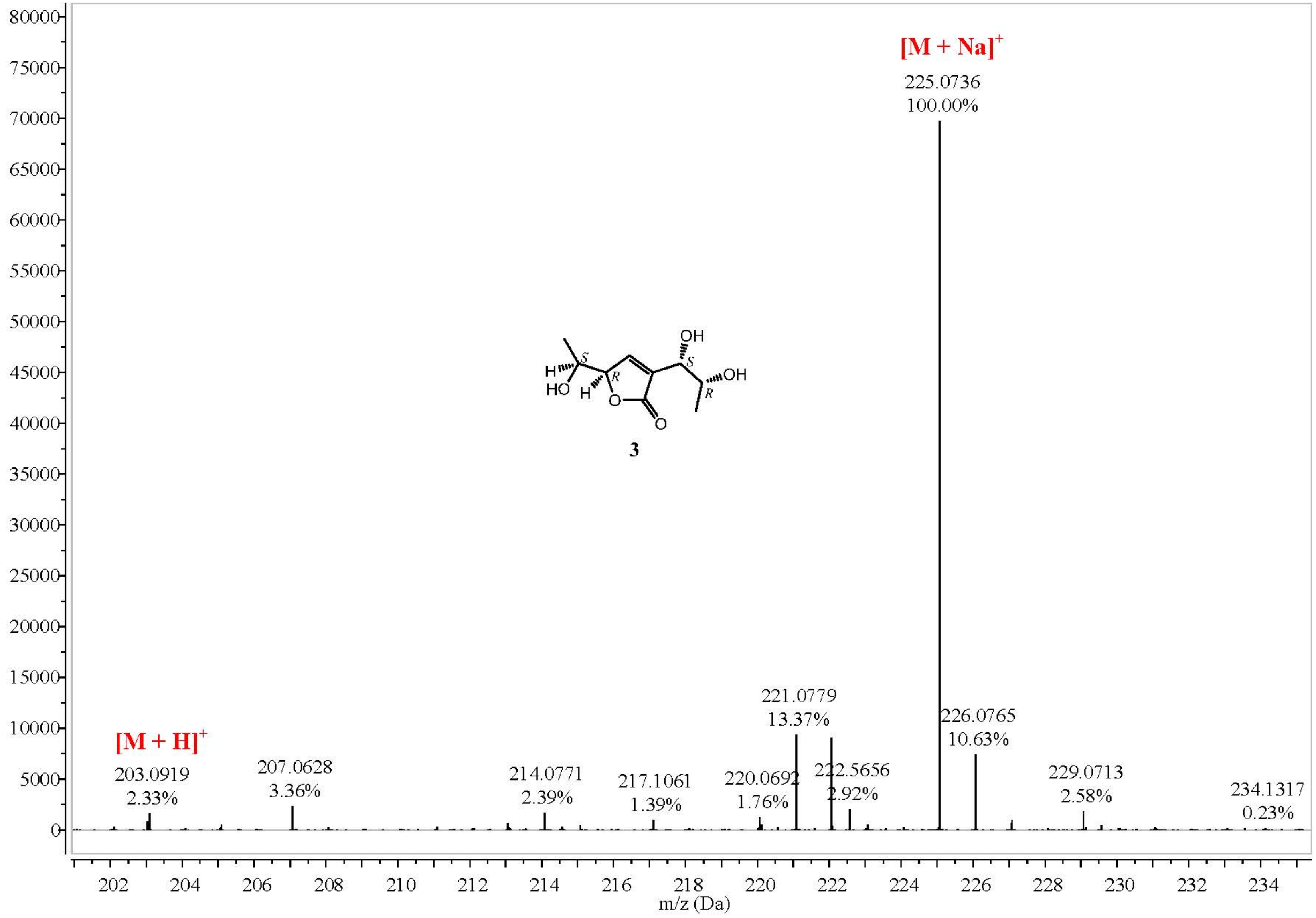

Figure S21. IR spectrum of **3**.

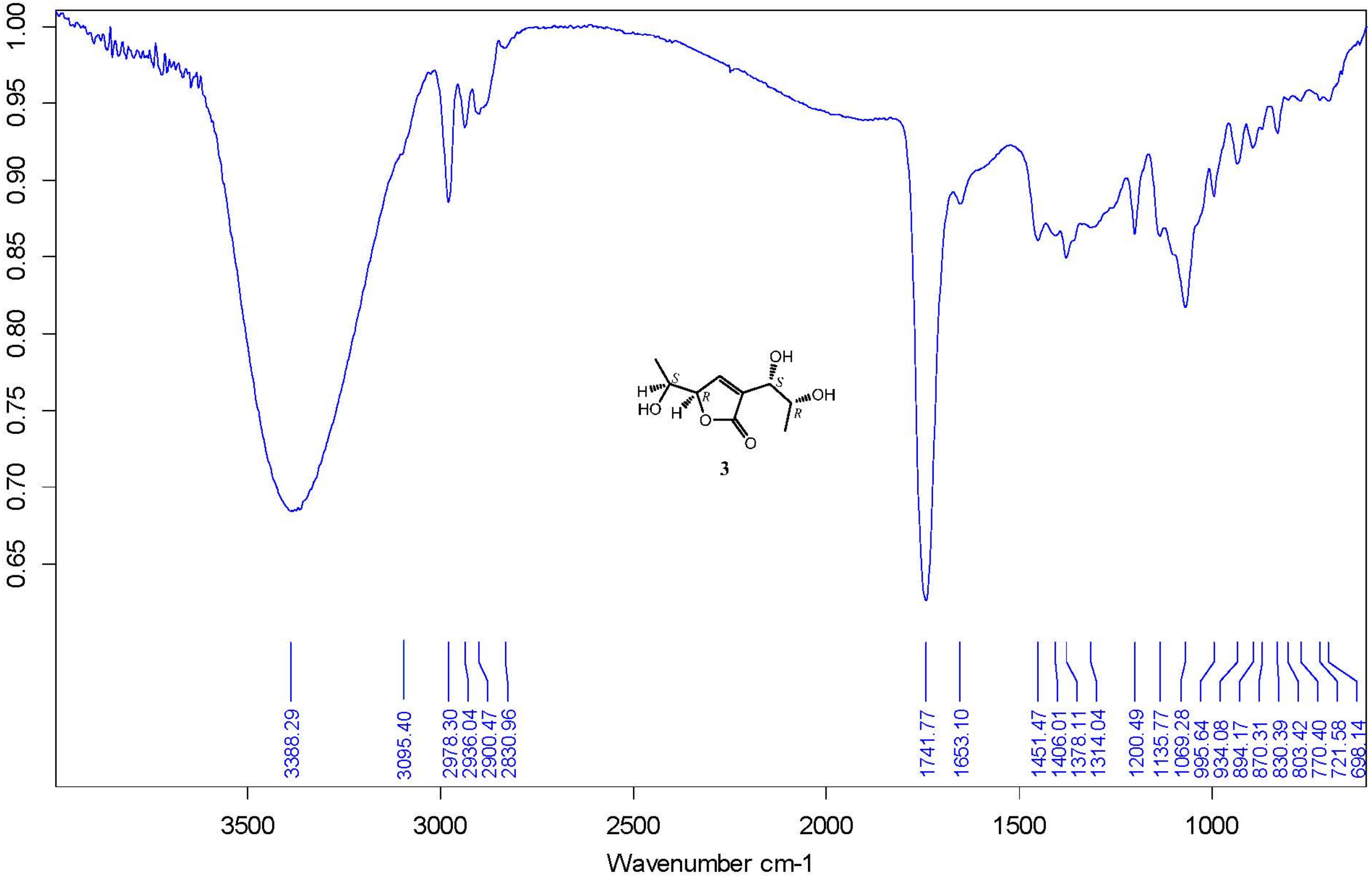

Figure S22. 400 MHz ^{1}H NMR spectrum of **3** in CD_3OD.

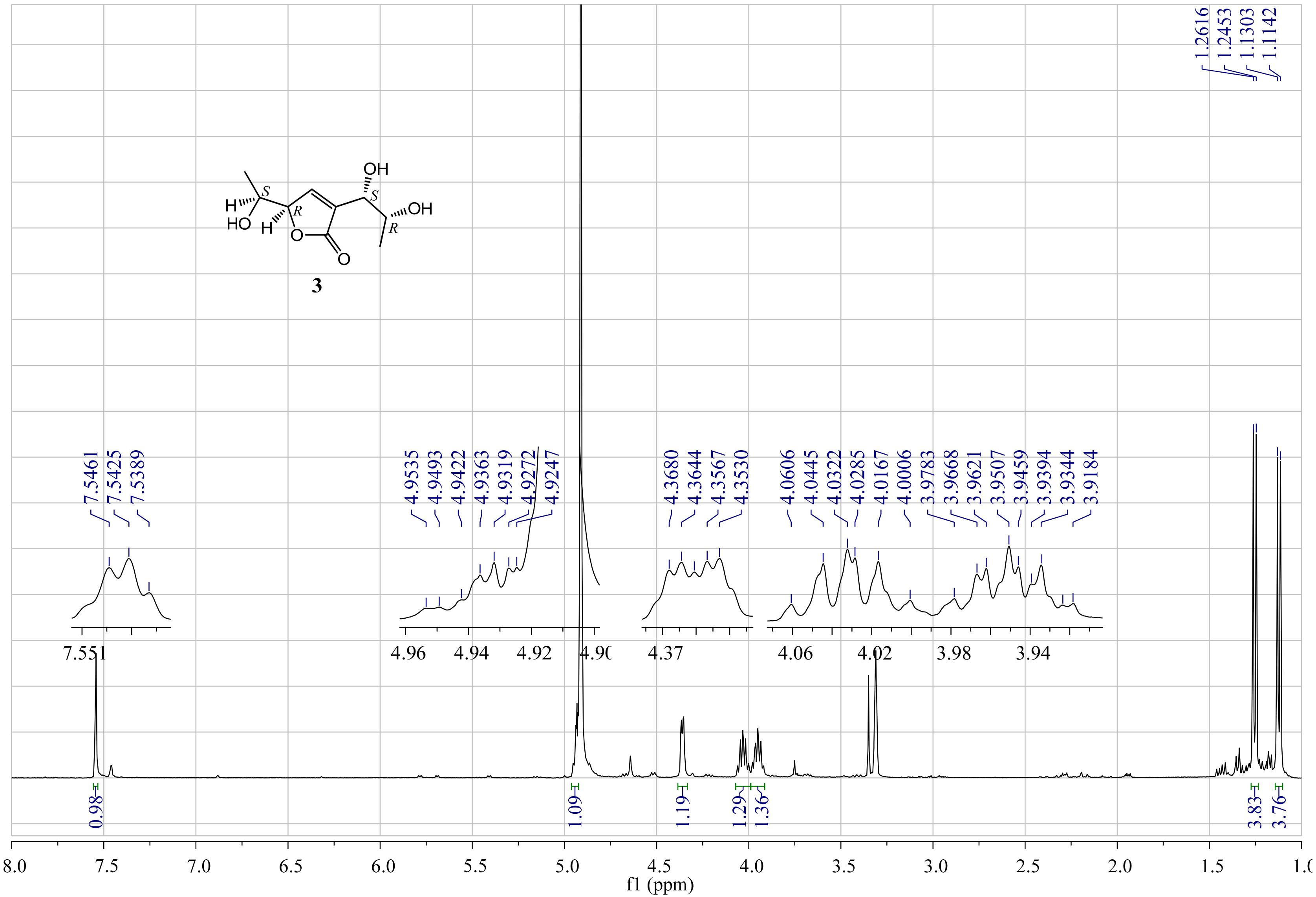

Figure S23. 100 MHz ^{13}C NMR spectrum of **3** in CD_3OD.

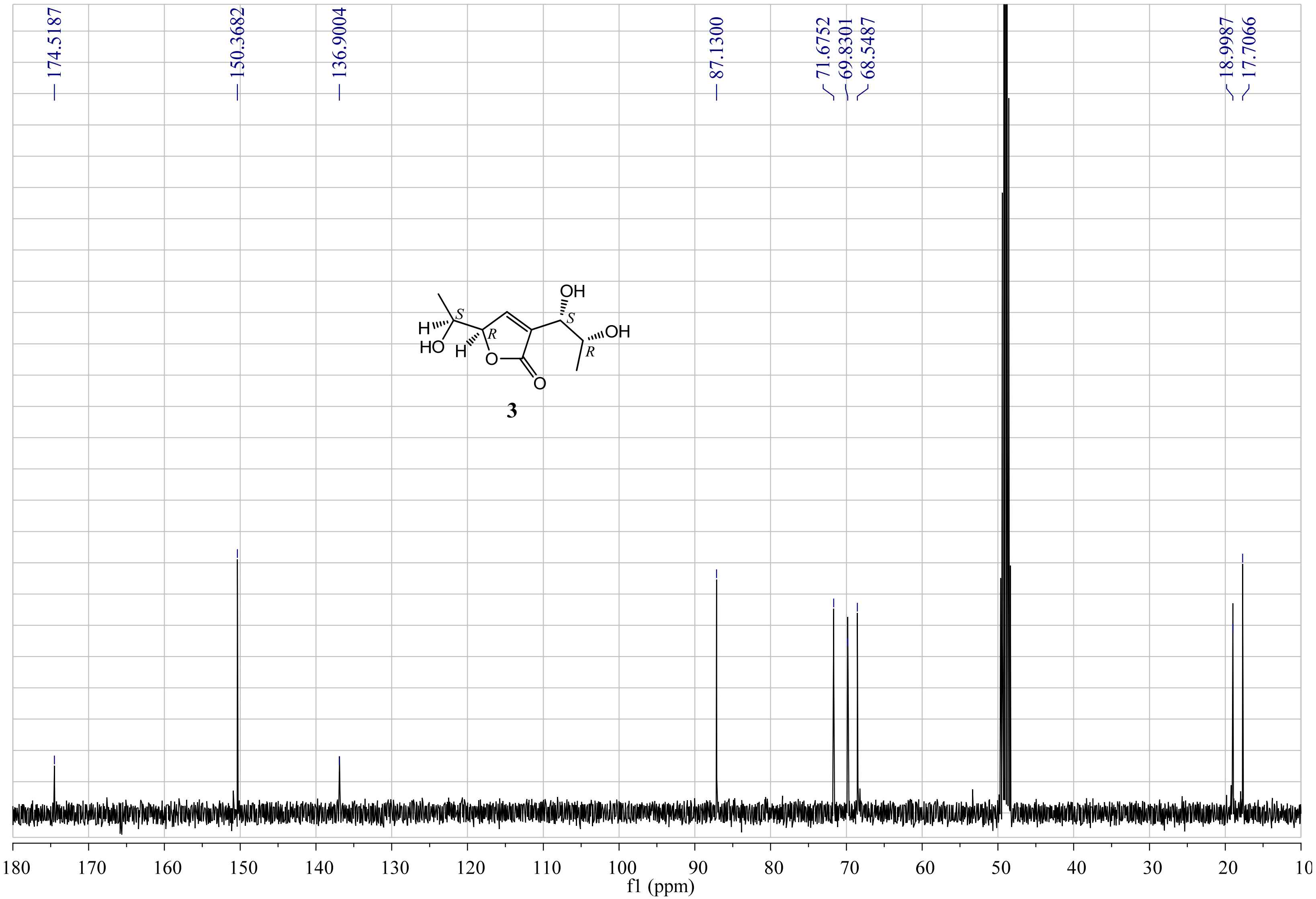

Figure S24. HMQC spectrum of **3** in CD_3OD.

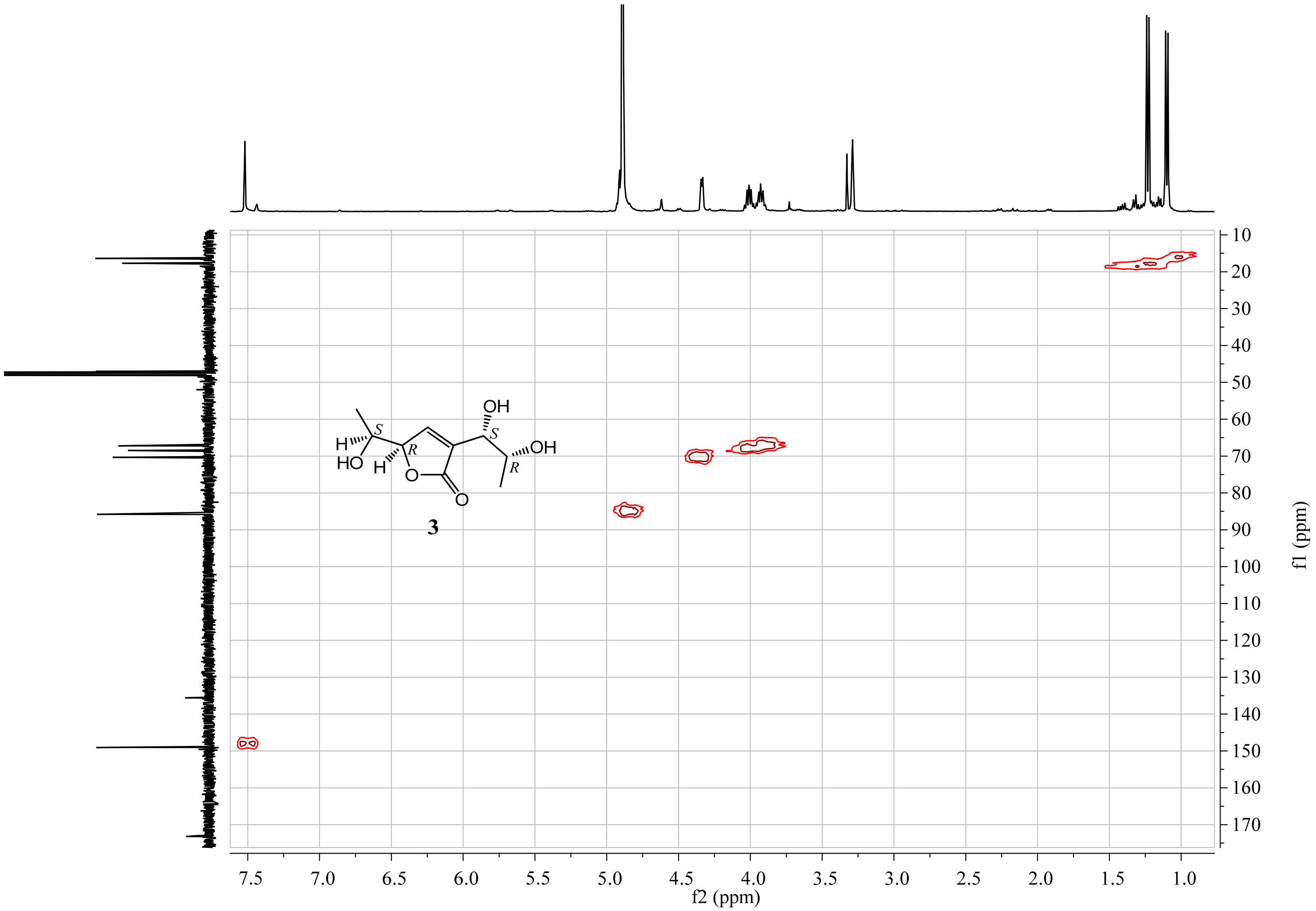

Figure S25. ^{1}H–^{1}H COSY spectrum of **3** in CD_3OD.

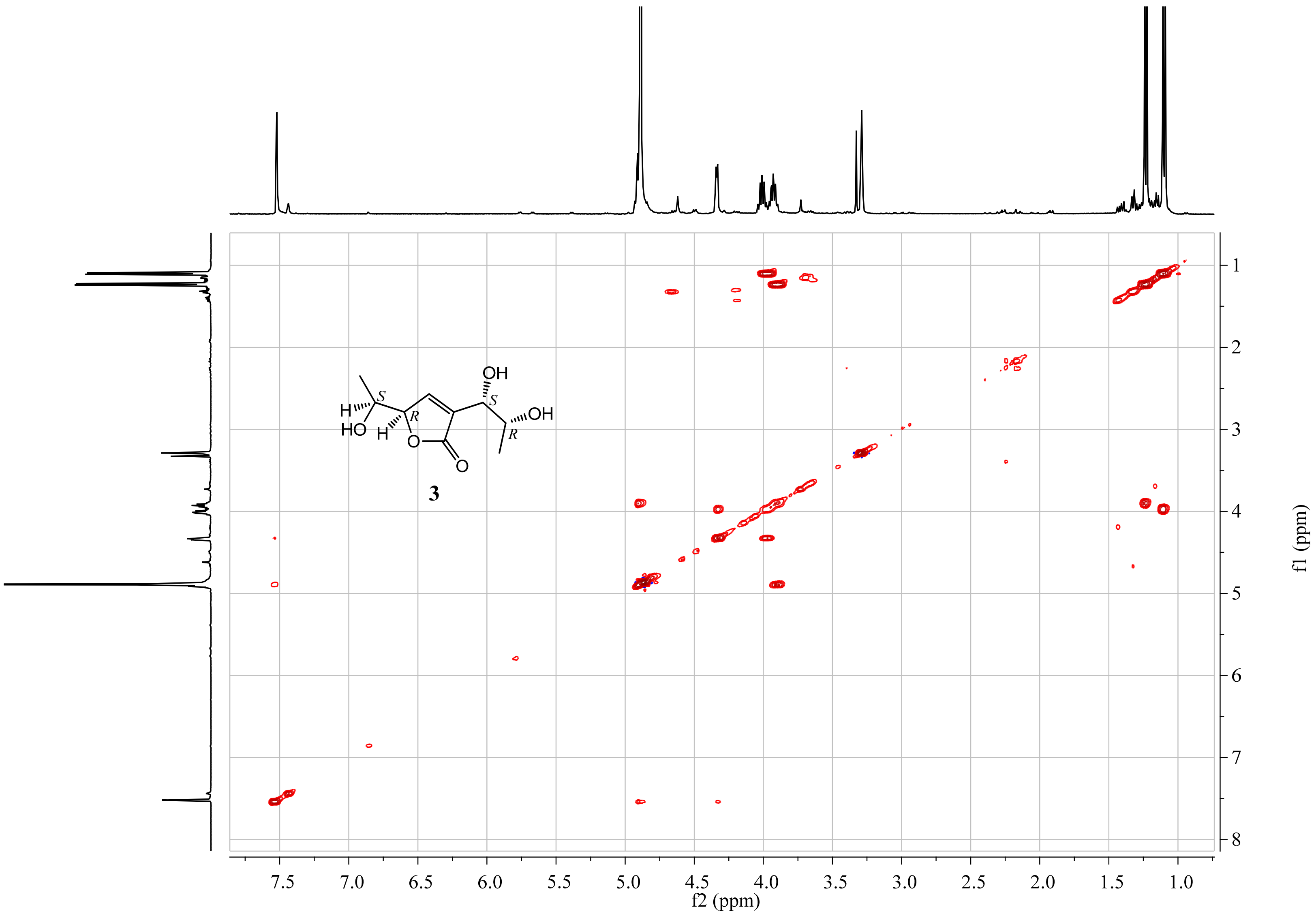

Figure S26. HMBC spectrum of **3** in CD_3OD.

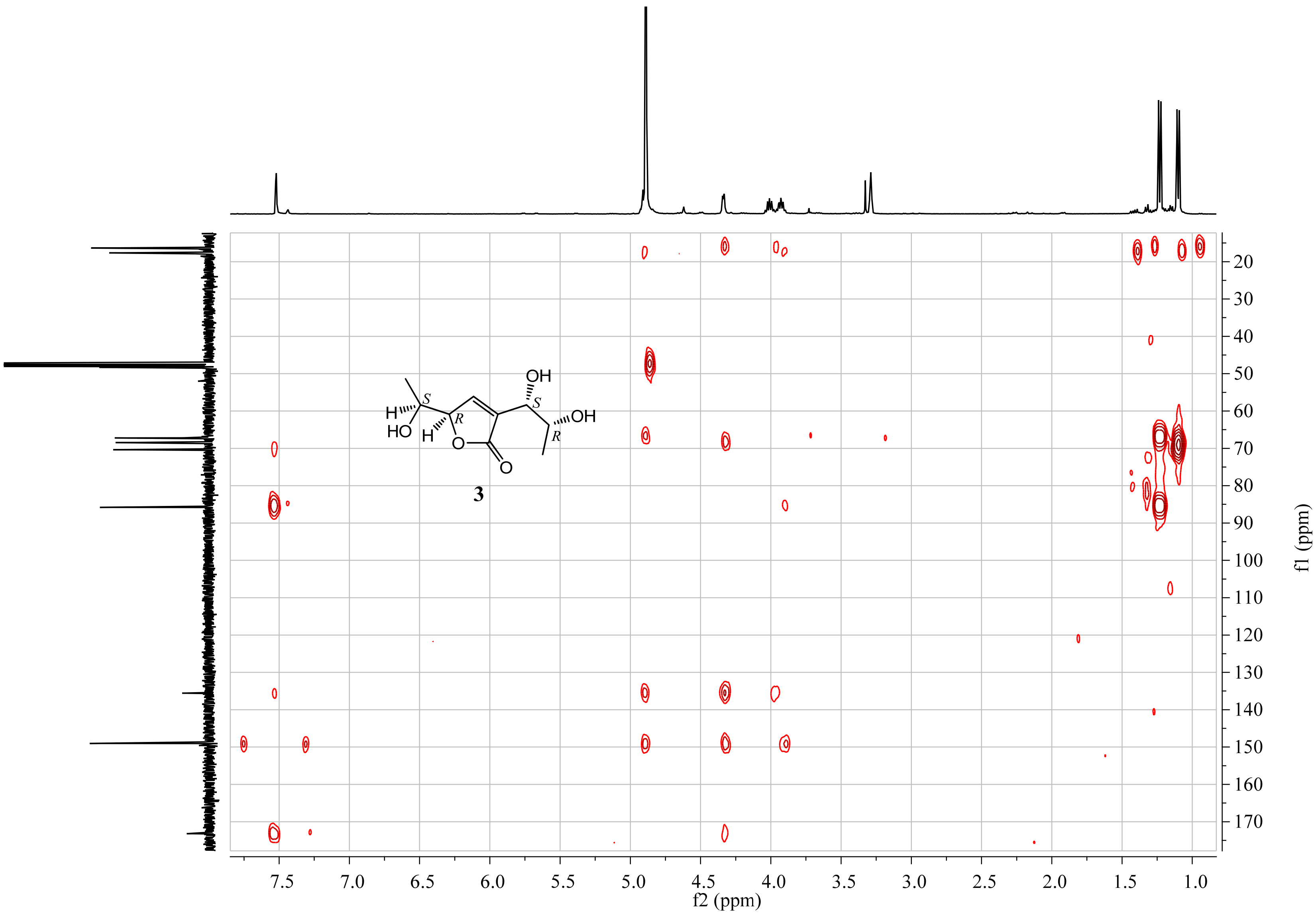

Figure S27. Positive ion ESI-MS spectrum of **4**.

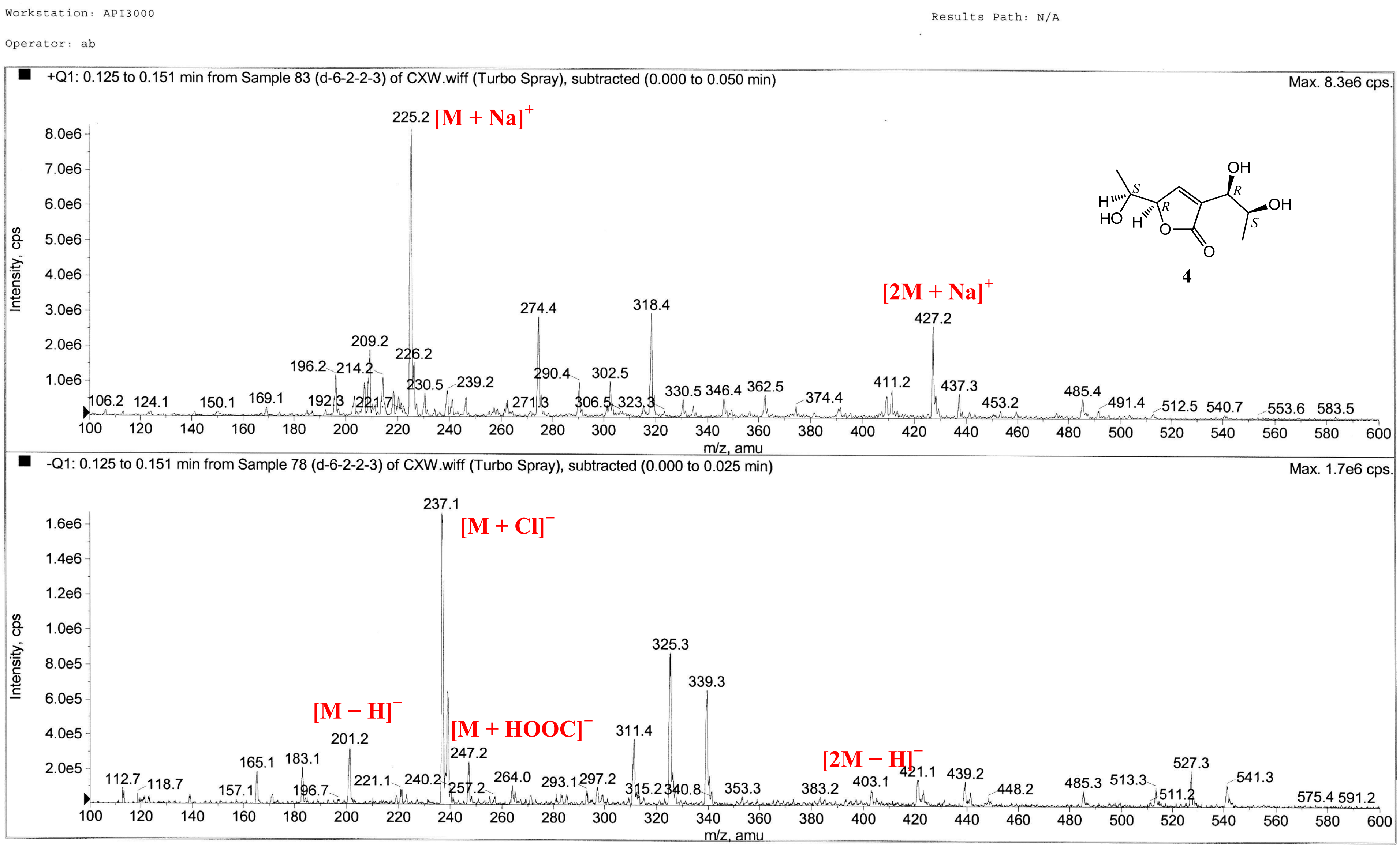

Figure S28. Positive ion HR-ESI-MS spectrum of **4**.

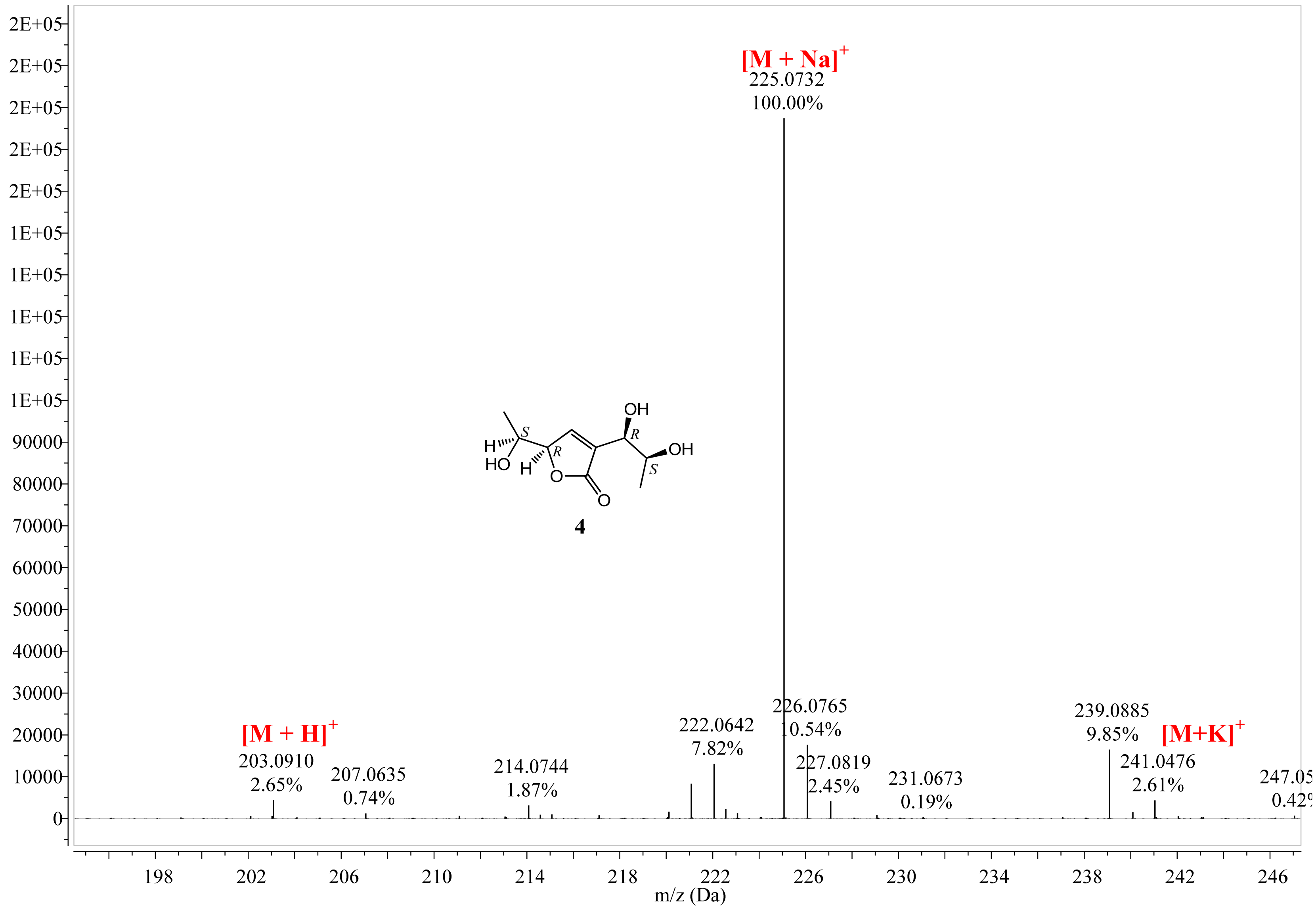

Figure S29. IR spectrum of **4**.

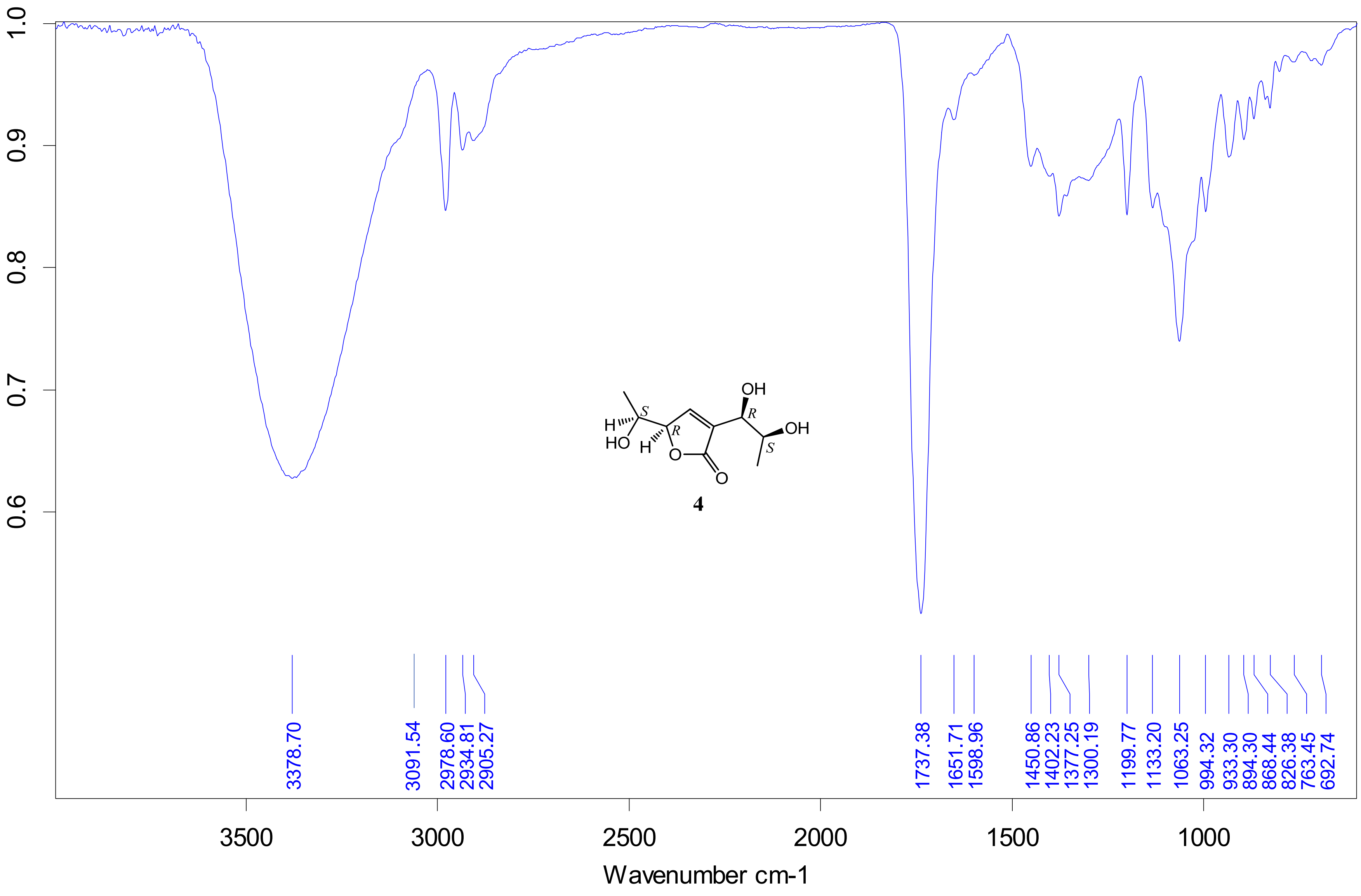

Figure S30. 400 MHz ^{1}H NMR spectrum of **4** in CD_3OD.

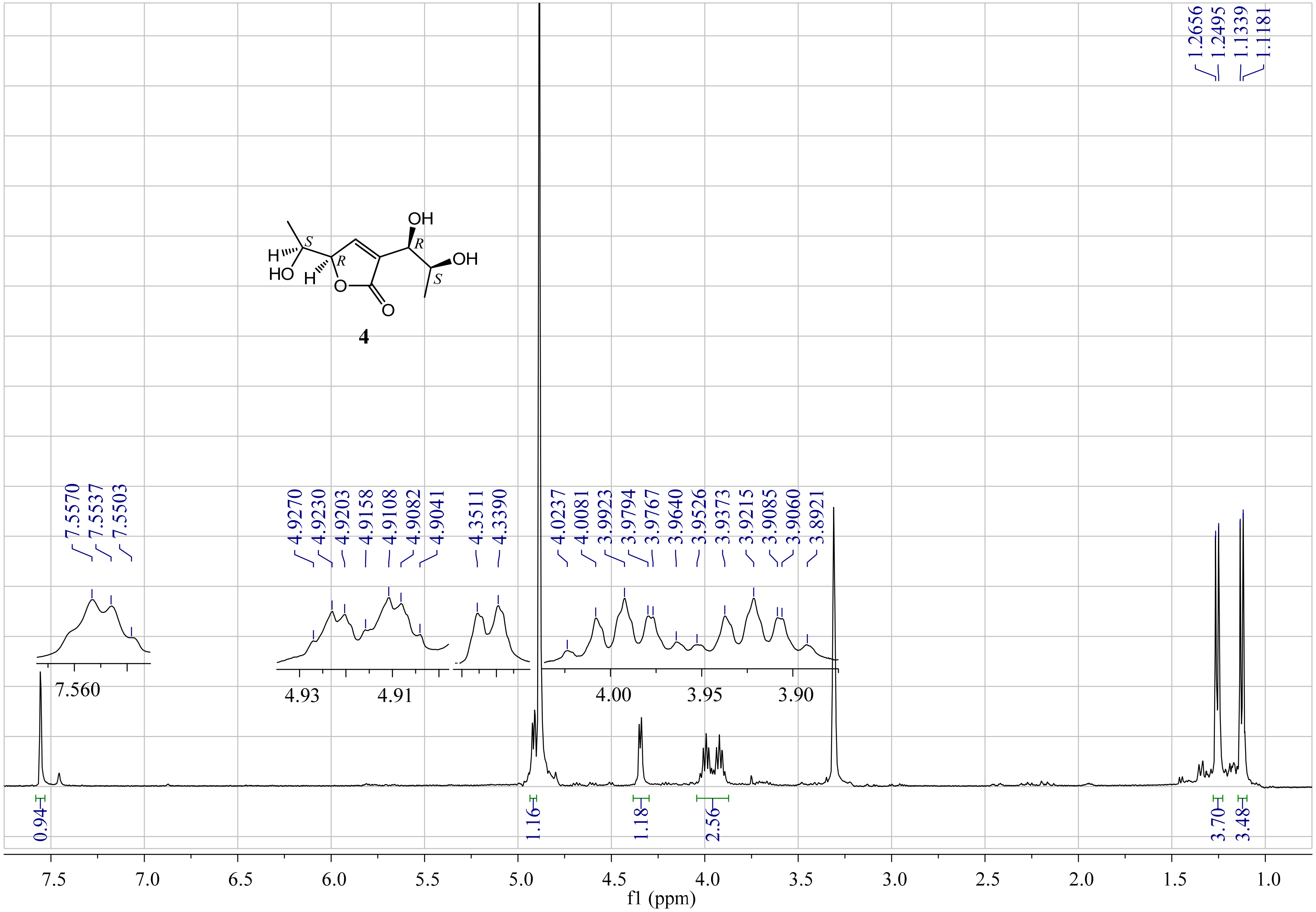

Figure S31. 100 MHz ^{13}C NMR spectrum of **4** in CD_3OD.

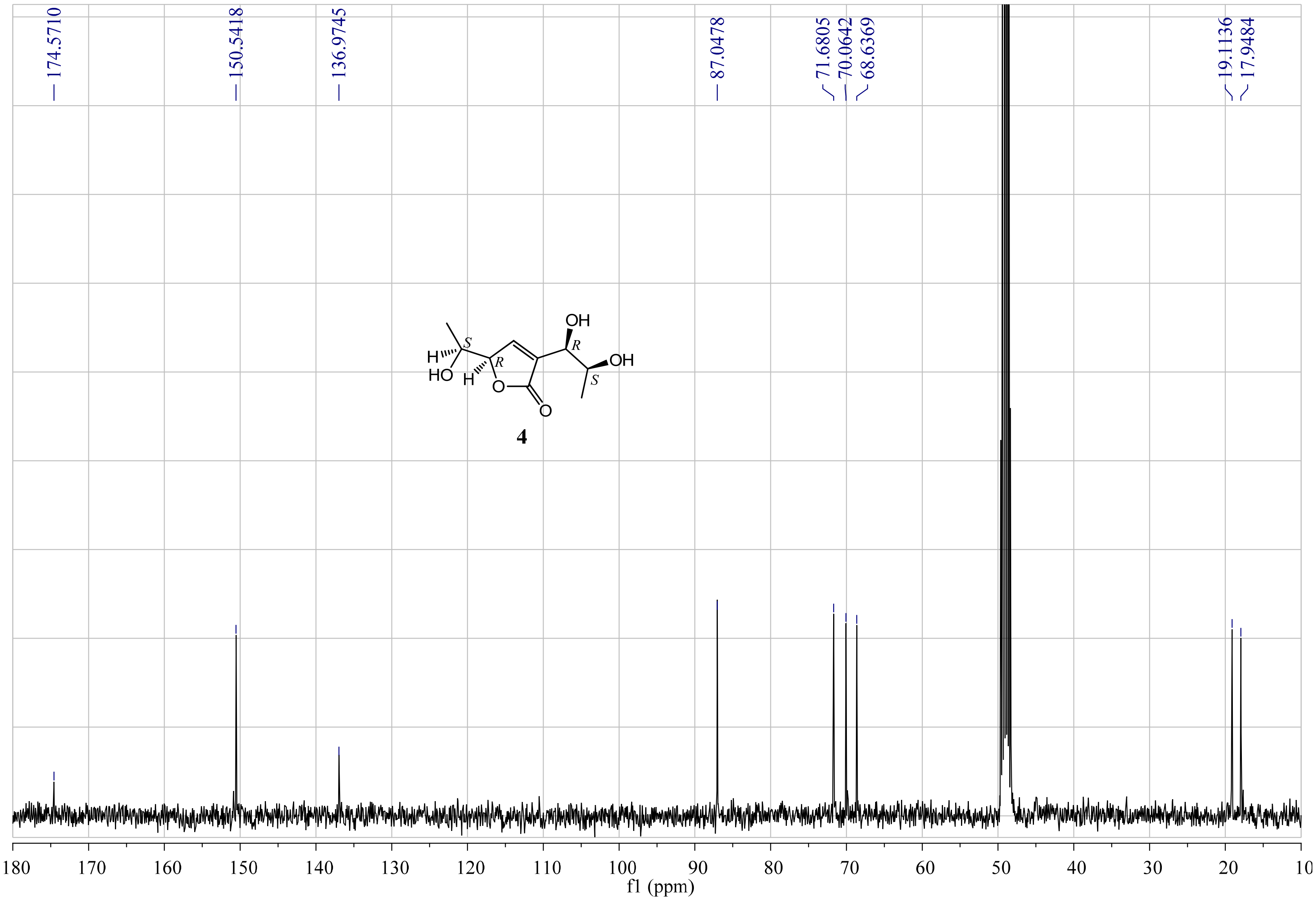

Figure S32. HMQC spectrum of **4** in CD_3OD.

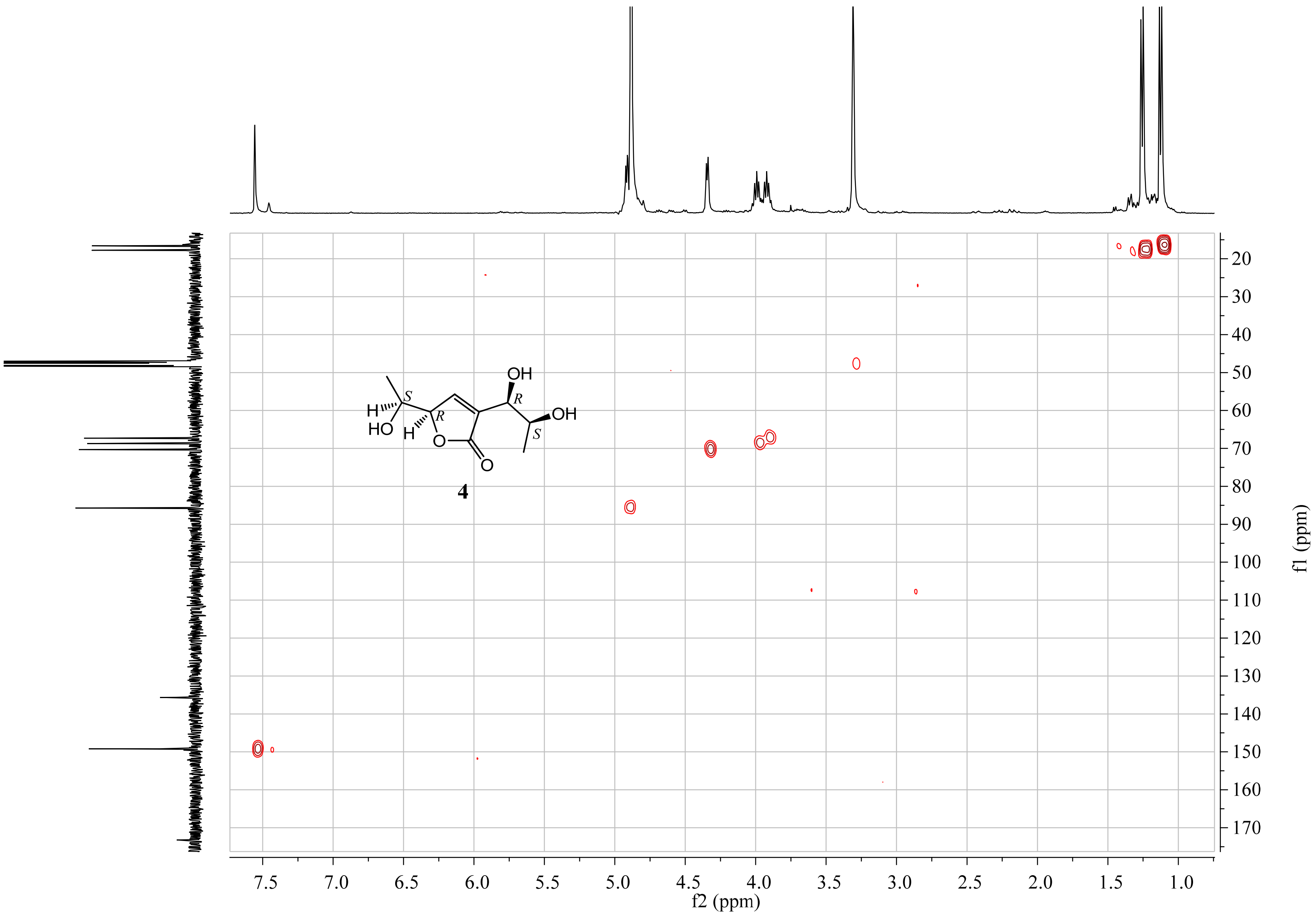

Figure S33. ^{1}H–^{1}H COSY spectrum of **4** in CD_3OD.

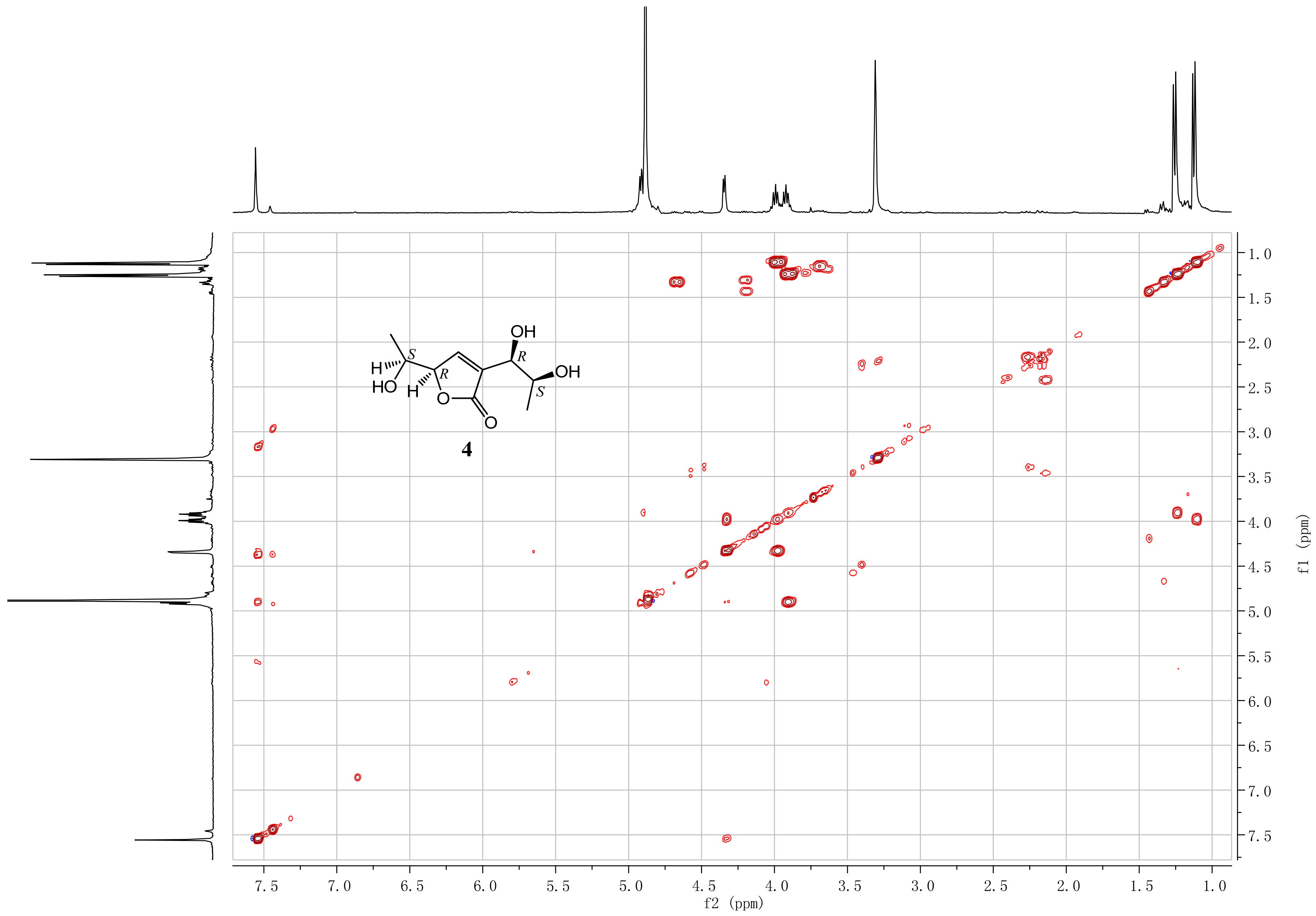

Figure S34. HMBC spectrum of **4** in CD_3OD.

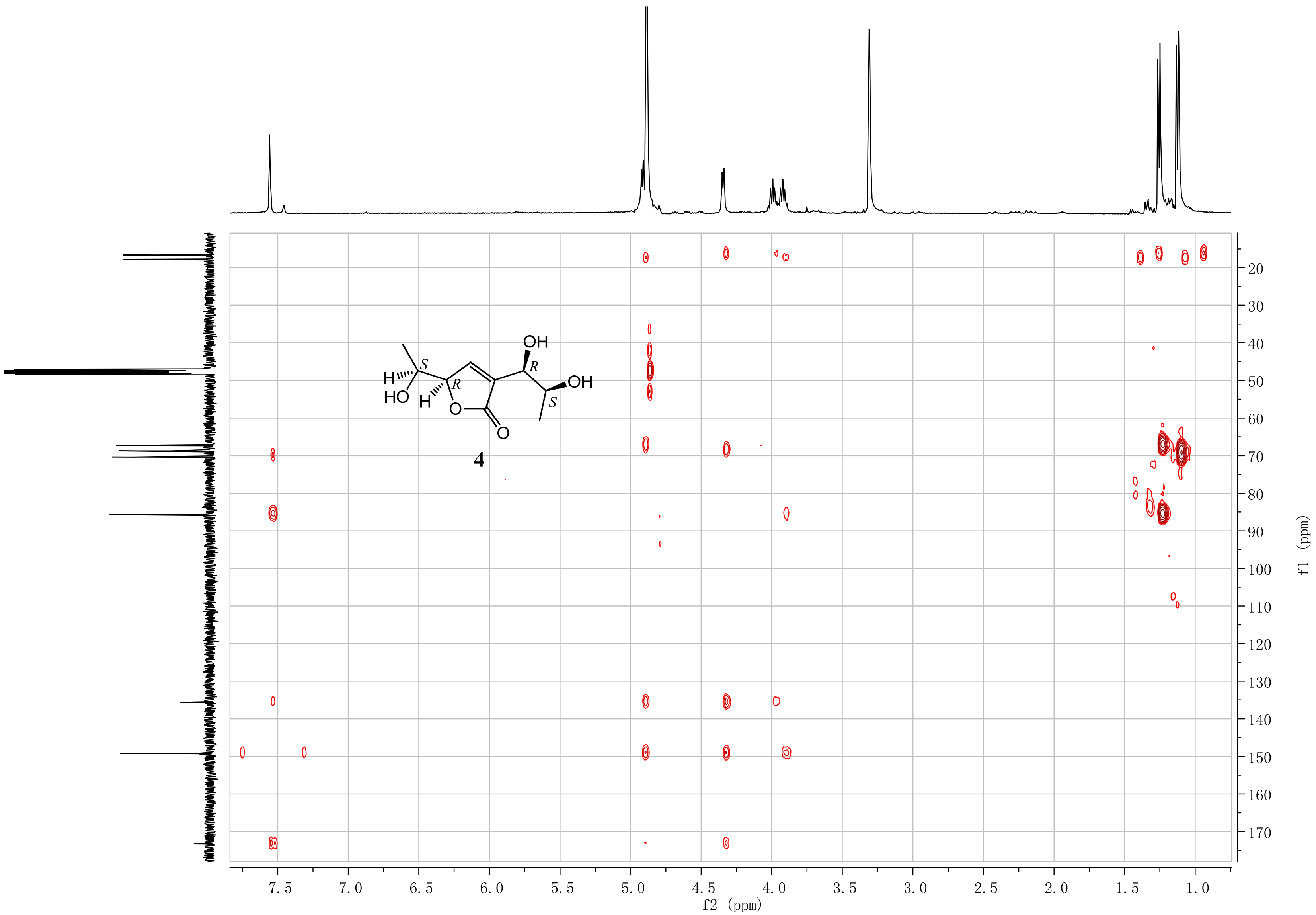

Figure S35. Positive ion ESI-MS spectrum of **5**.

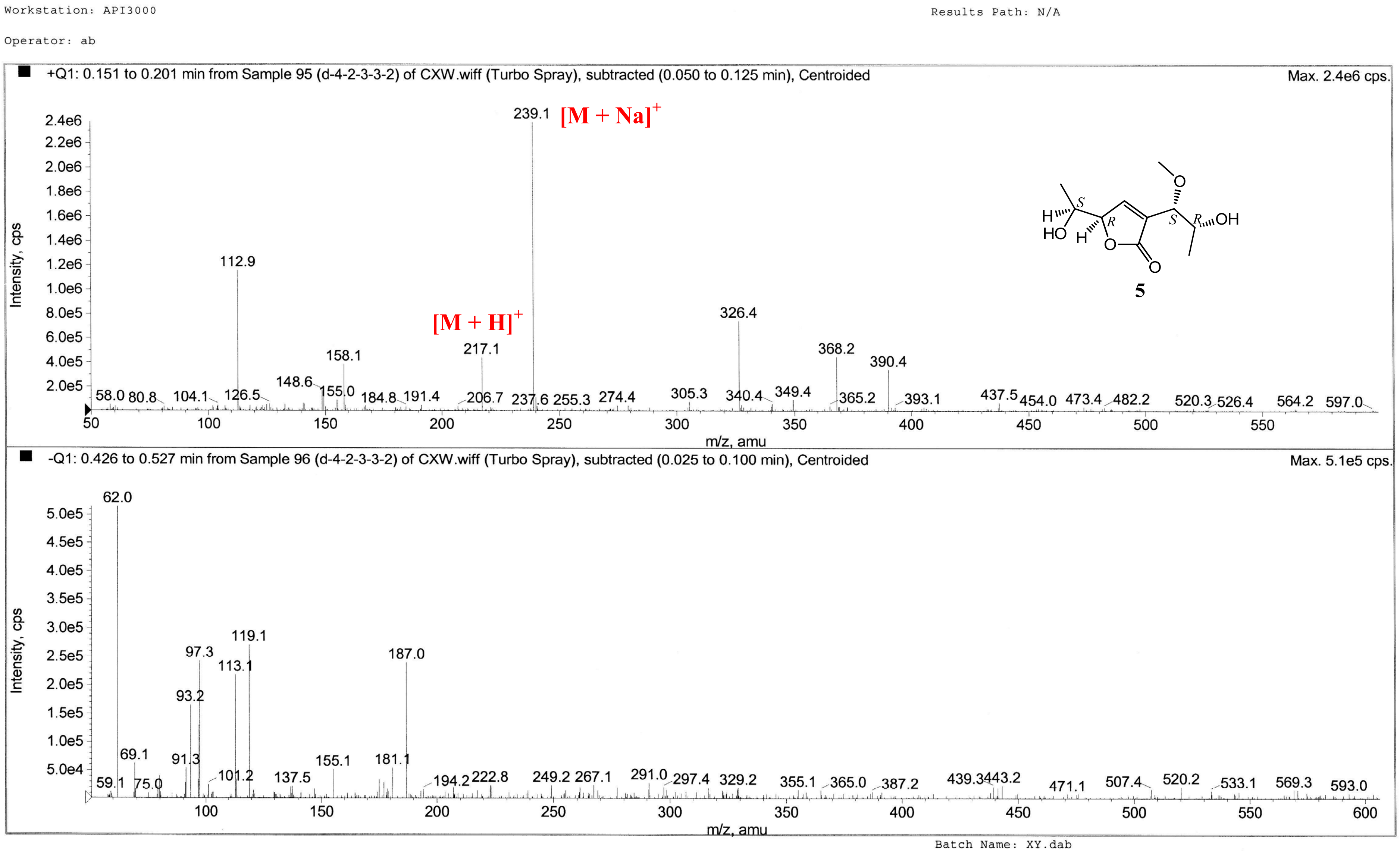

Figure S36. Positive ion HR-ESI-MS spectrum of **5**.

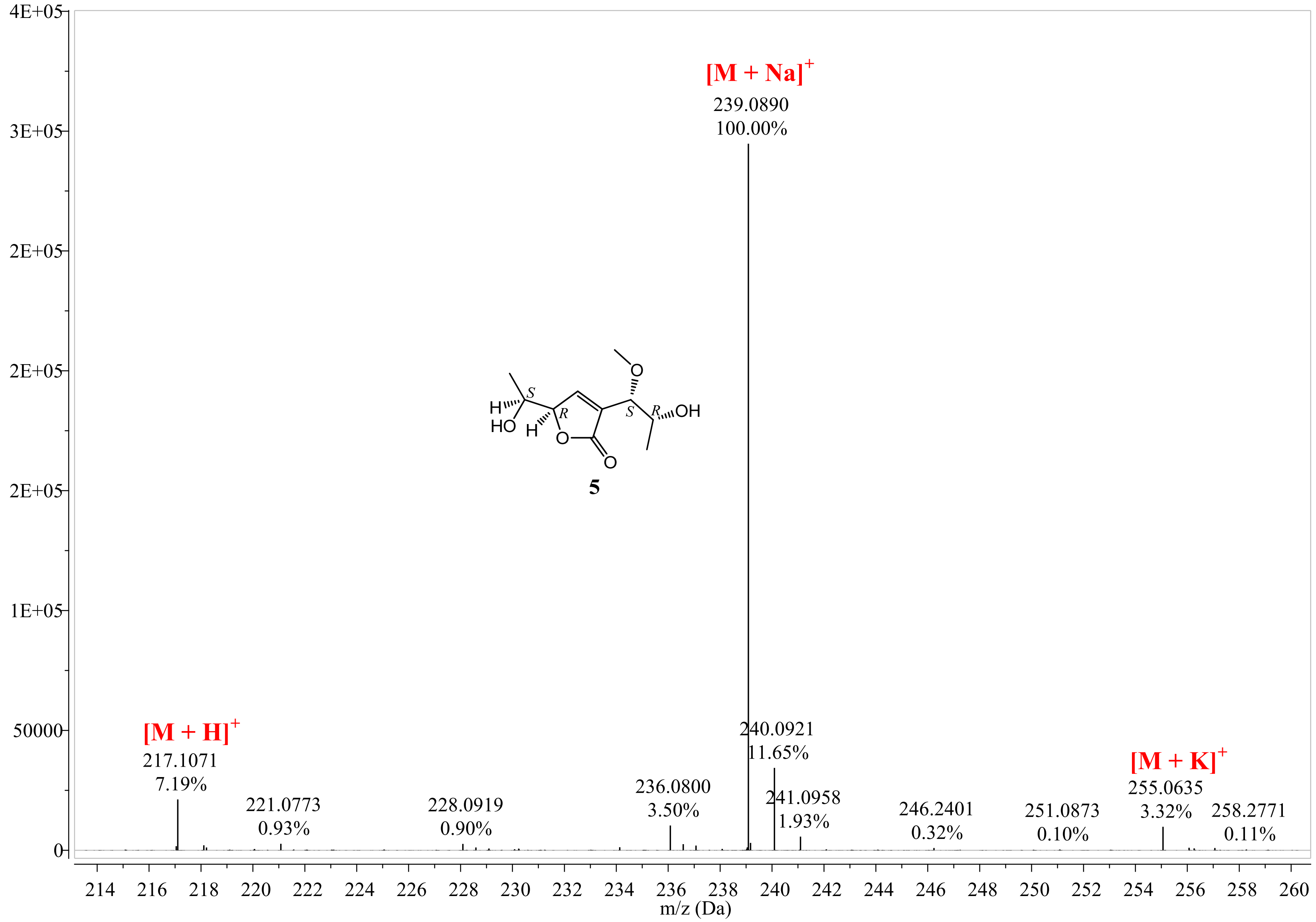

Figure S37. IR spectrum of **5**.

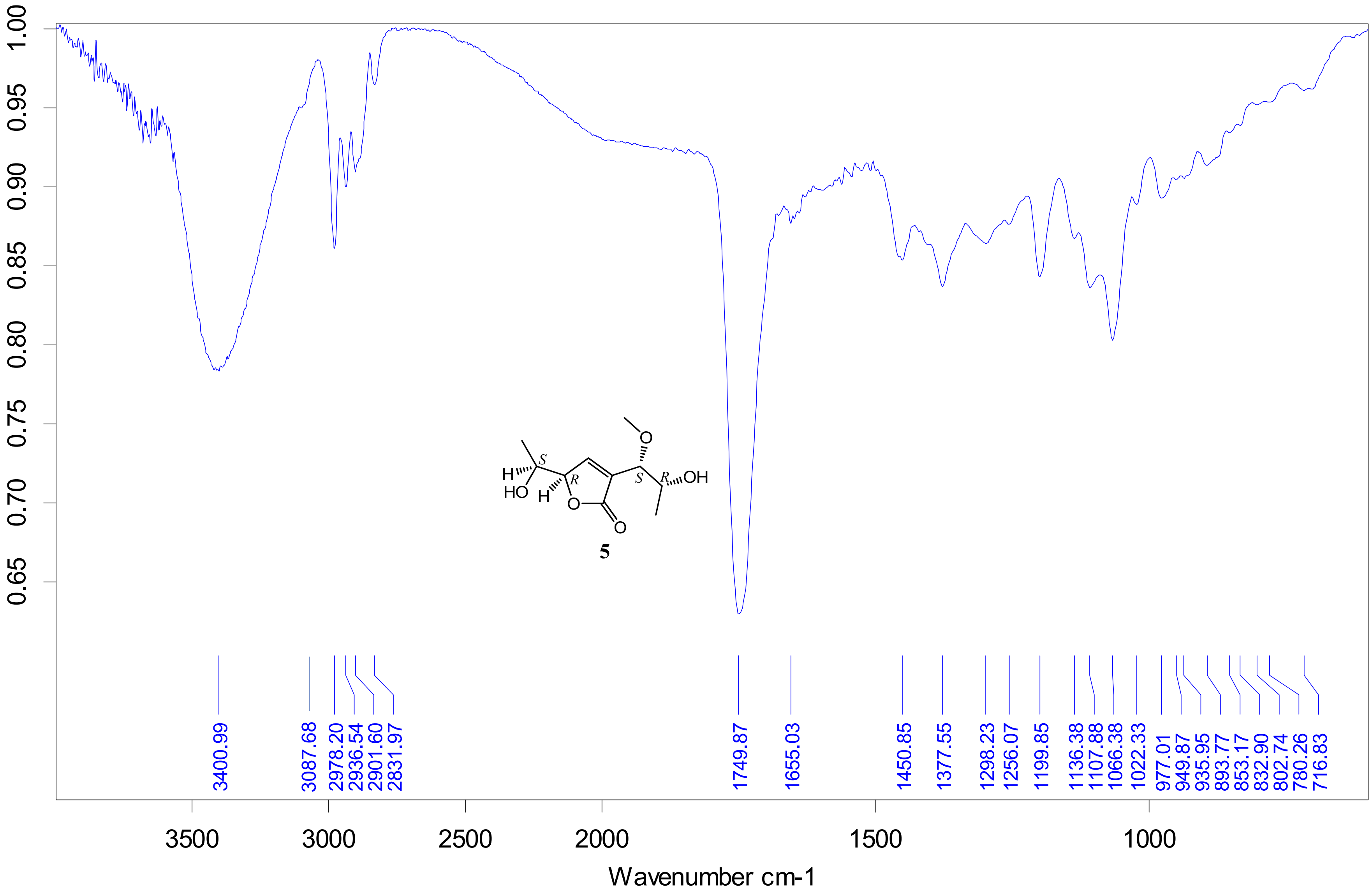

Figure S38. 400 MHz ^{1}H NMR spectrum of **5** in CD_3OD.

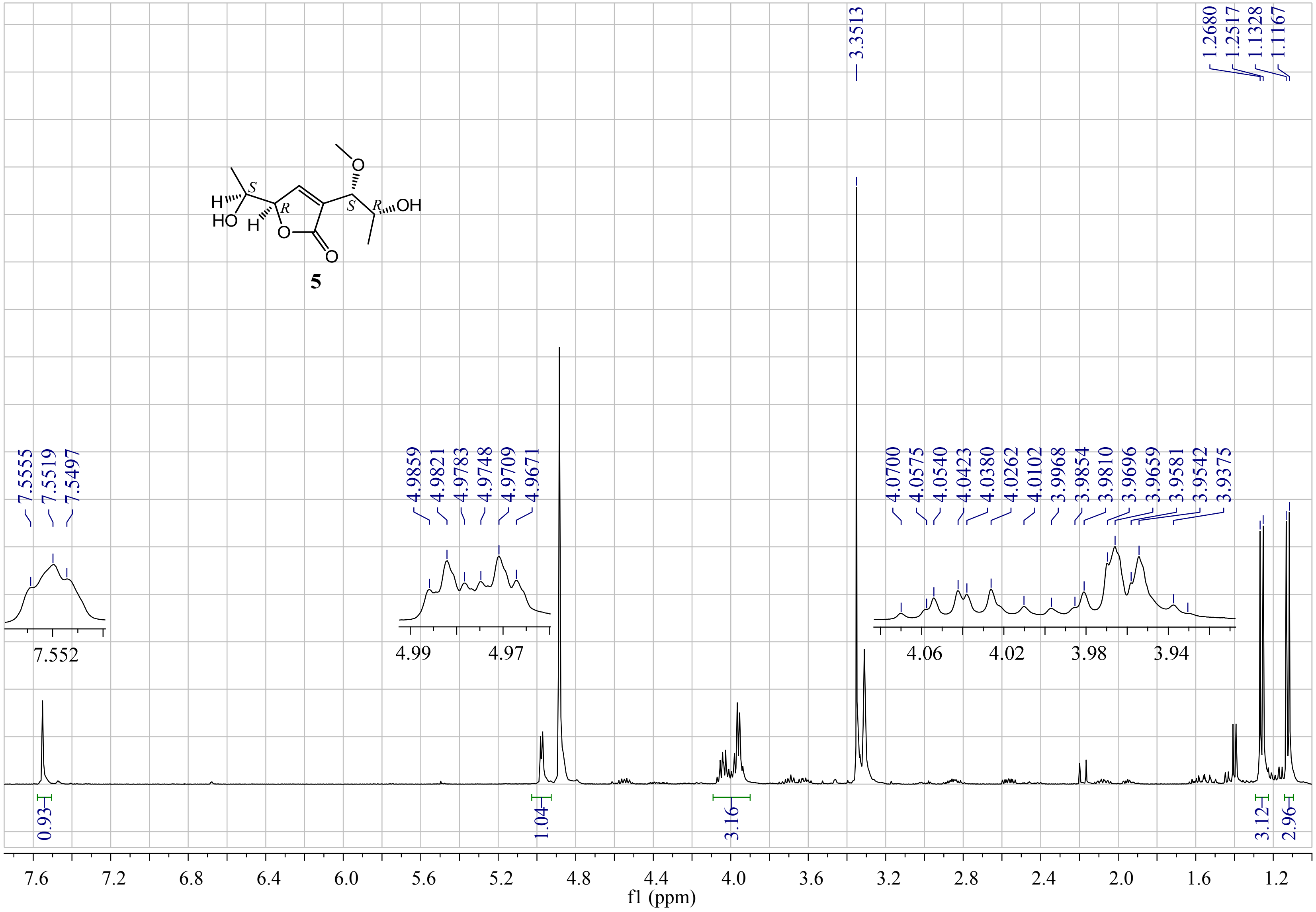

Figure S39. 100 MHz ^{13}C NMR spectrum of **5** in CD_3OD.

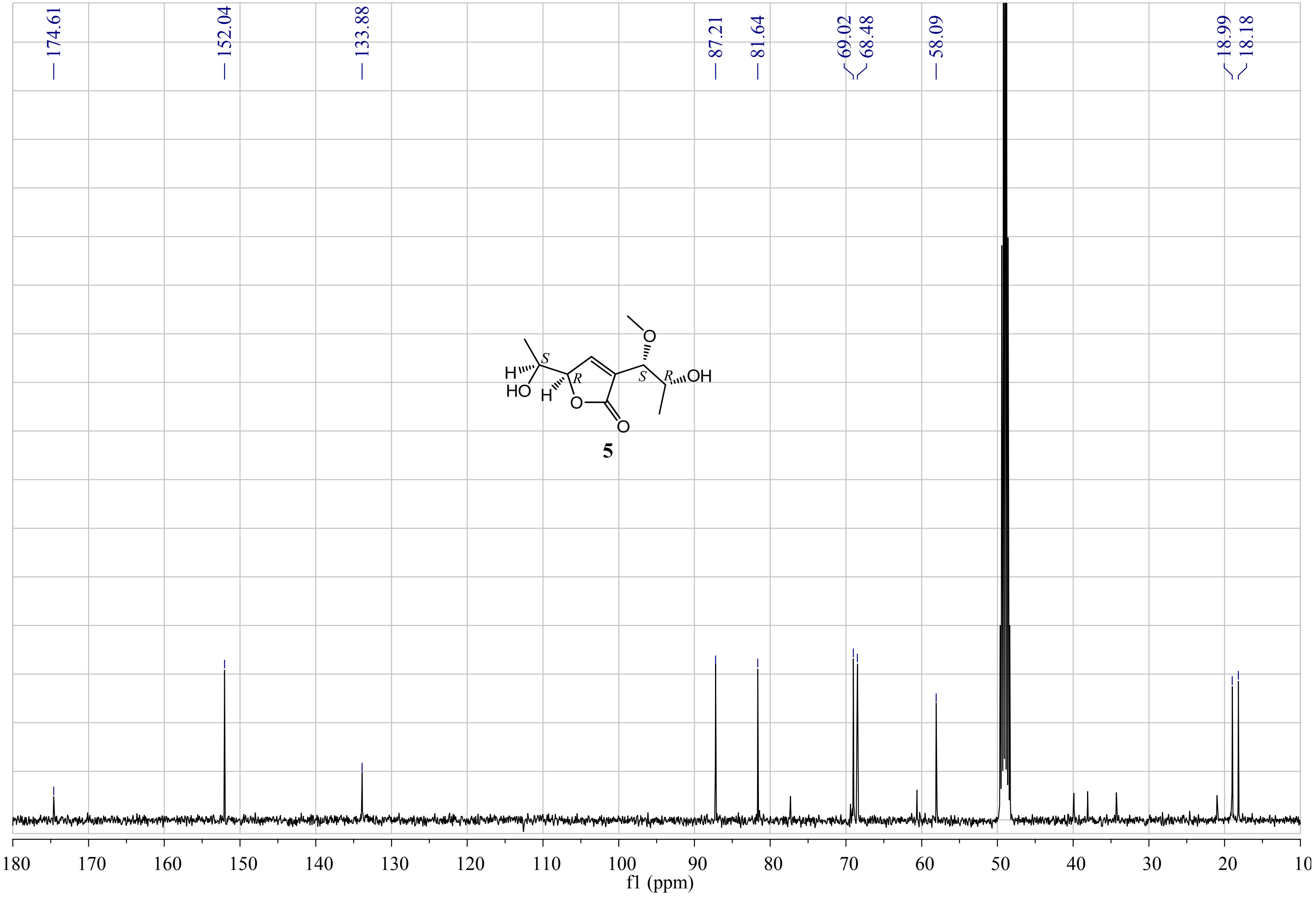

Figure S40. HMQC spectrum of **5** in CD_3OD.

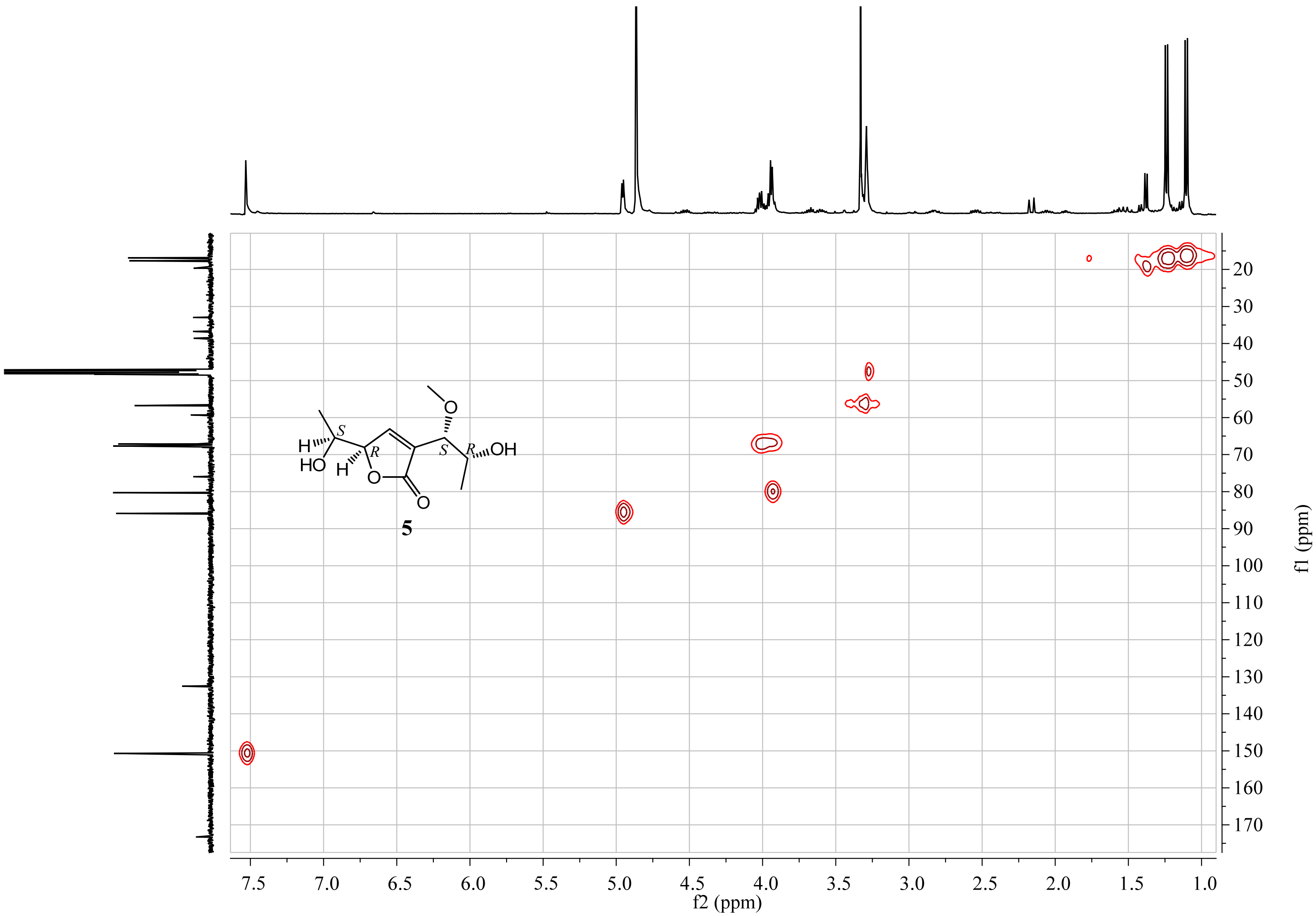

Figure S41. ^{1}H–^{1}H COSY spectrum of **5** in CD_3OD.

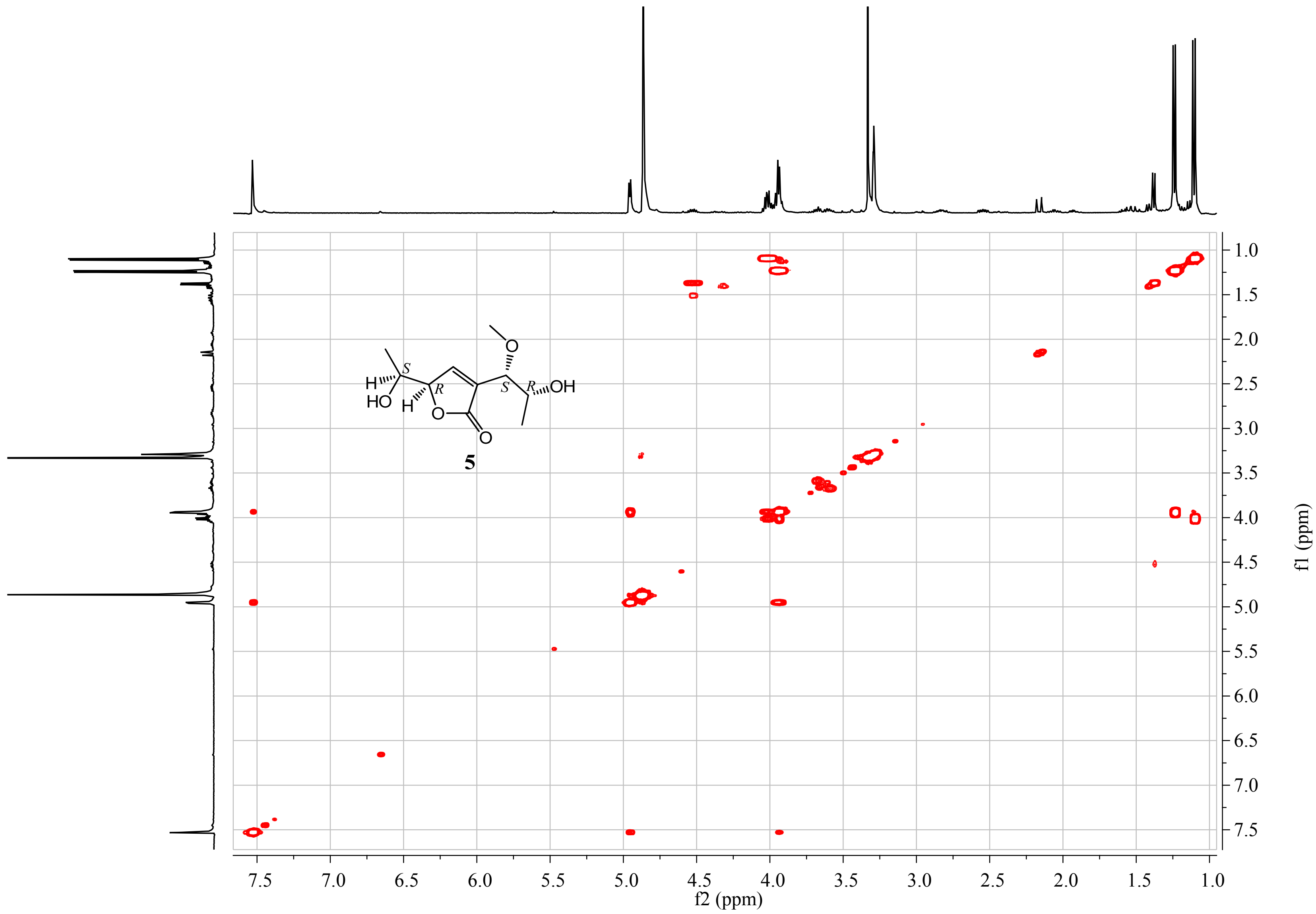

Figure S42. HMBC spectrum of **5** in CD_3OD.

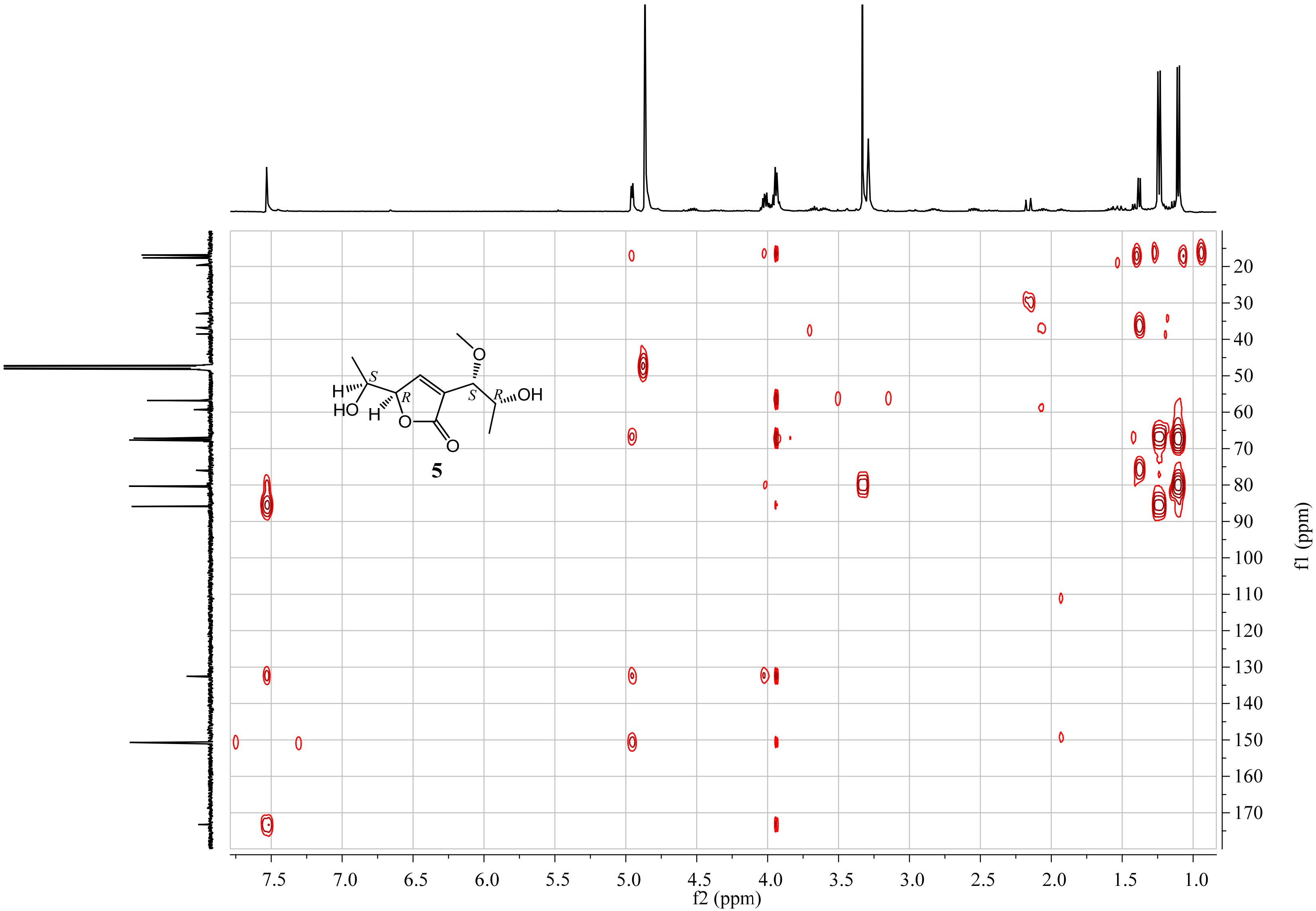

Figure S43. 400 MHz ^{1}H NMR spectrum of the (*S*)- and (*R*)-MTPA esters of **5** in pyridine-d_5.

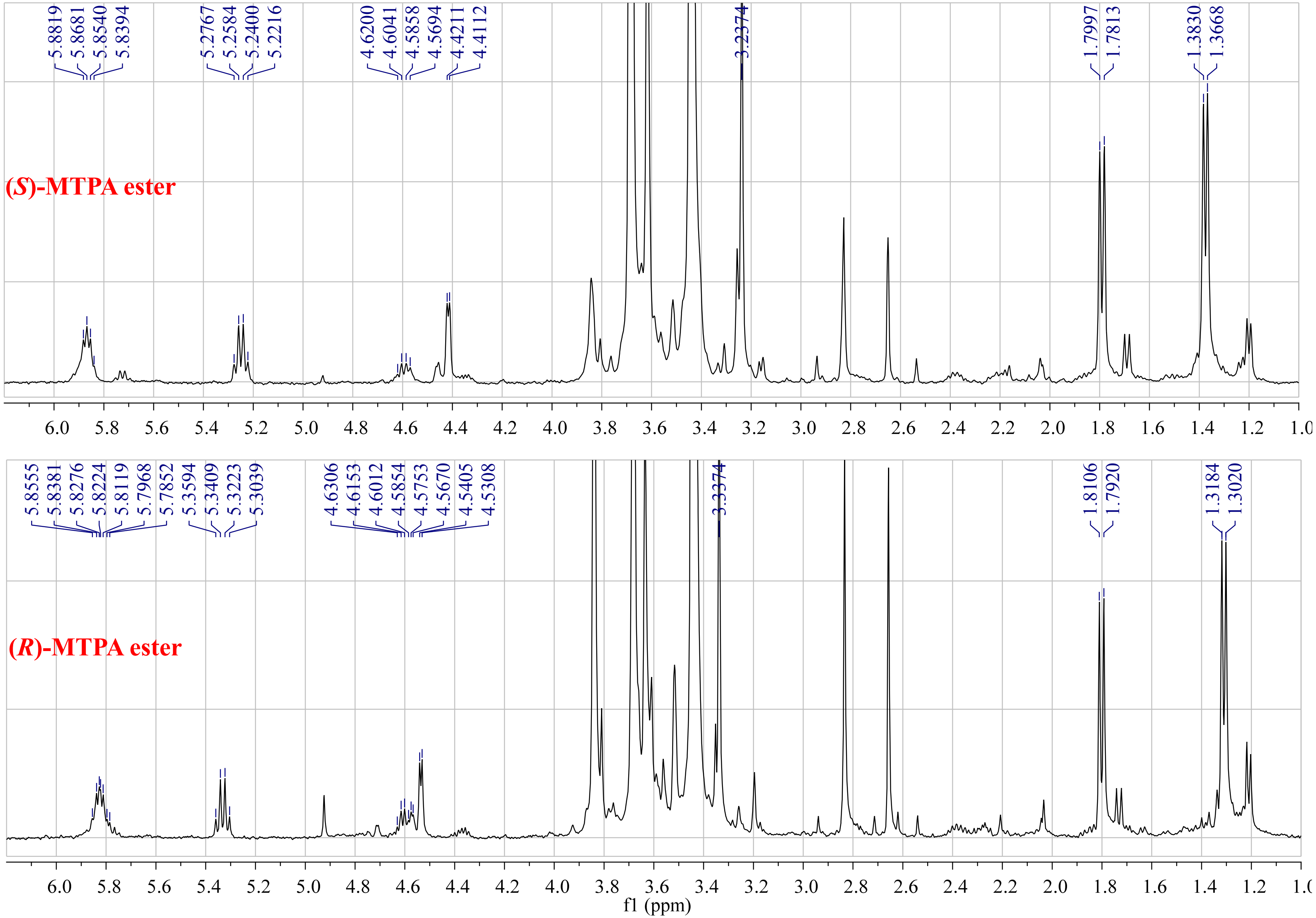

Figure S44. Positive ion ESI-MS spectrum of **6**/**7**.

Workstation: API3000 Results Path: N/A

Operator: ab

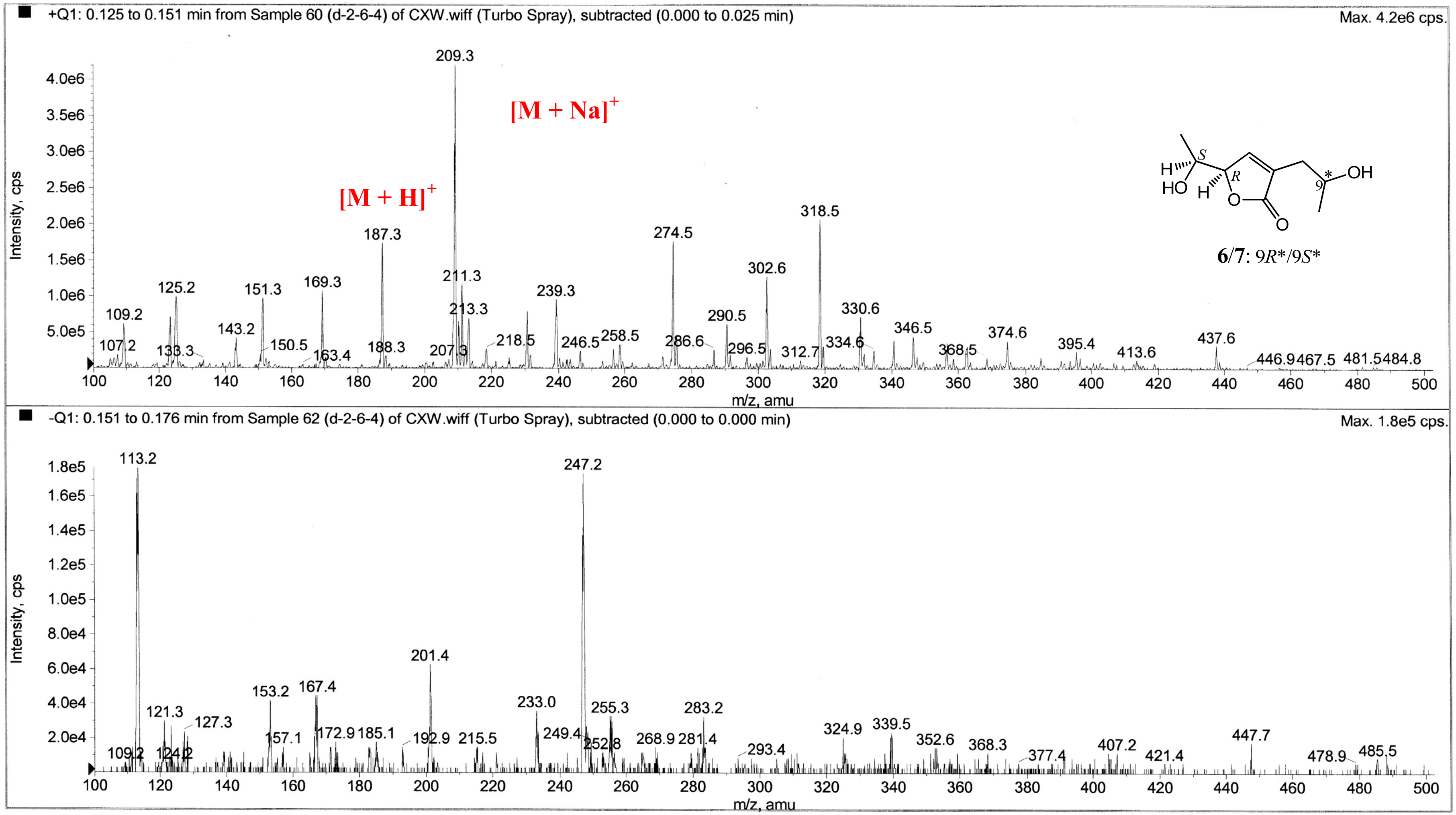

Batch Name: XY.dab

Figure S45. Positive ion HR-ESI-MS spectrum of **6**/**7**.

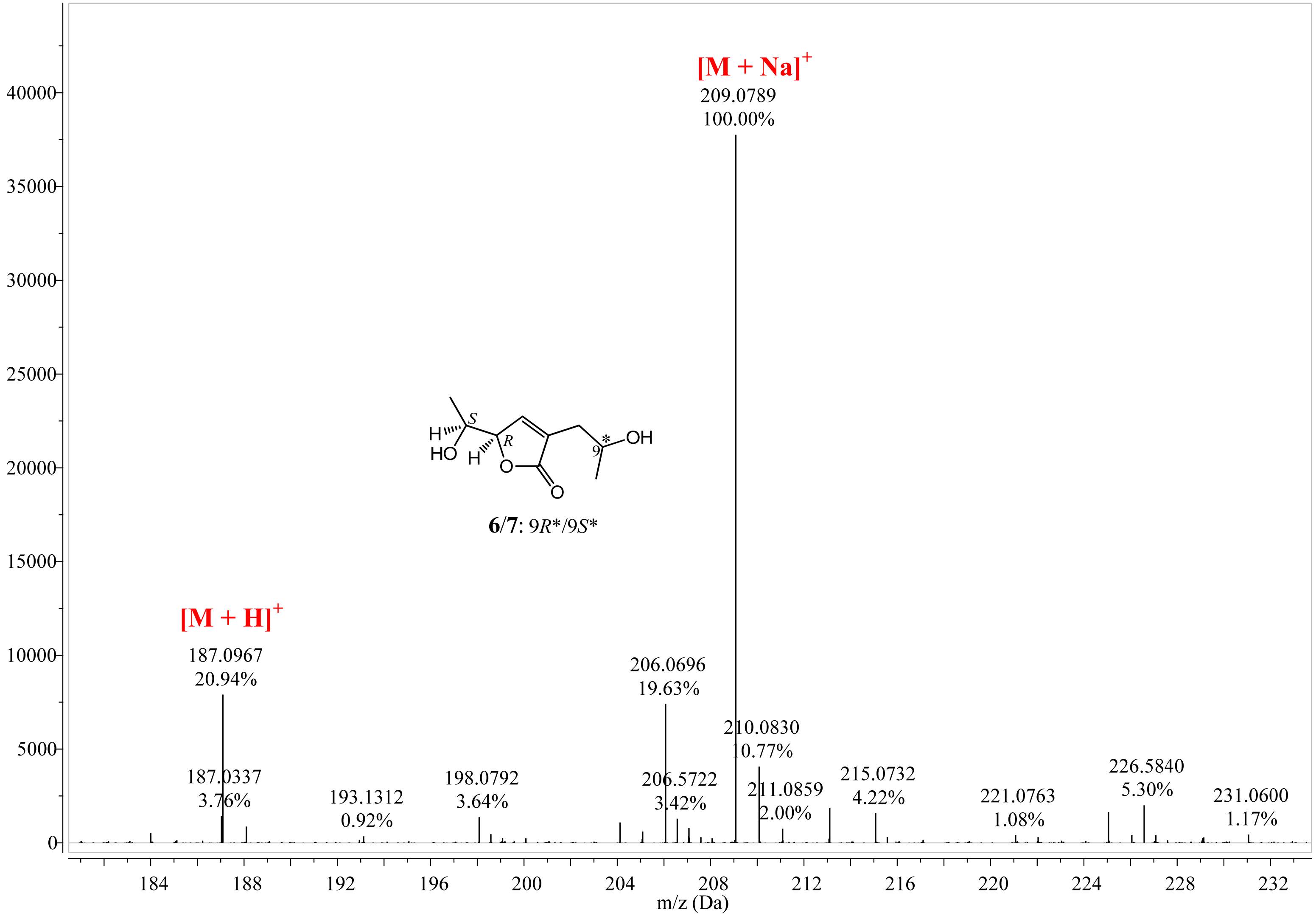

Figure S46. IR spectrum of **6**/**7**.

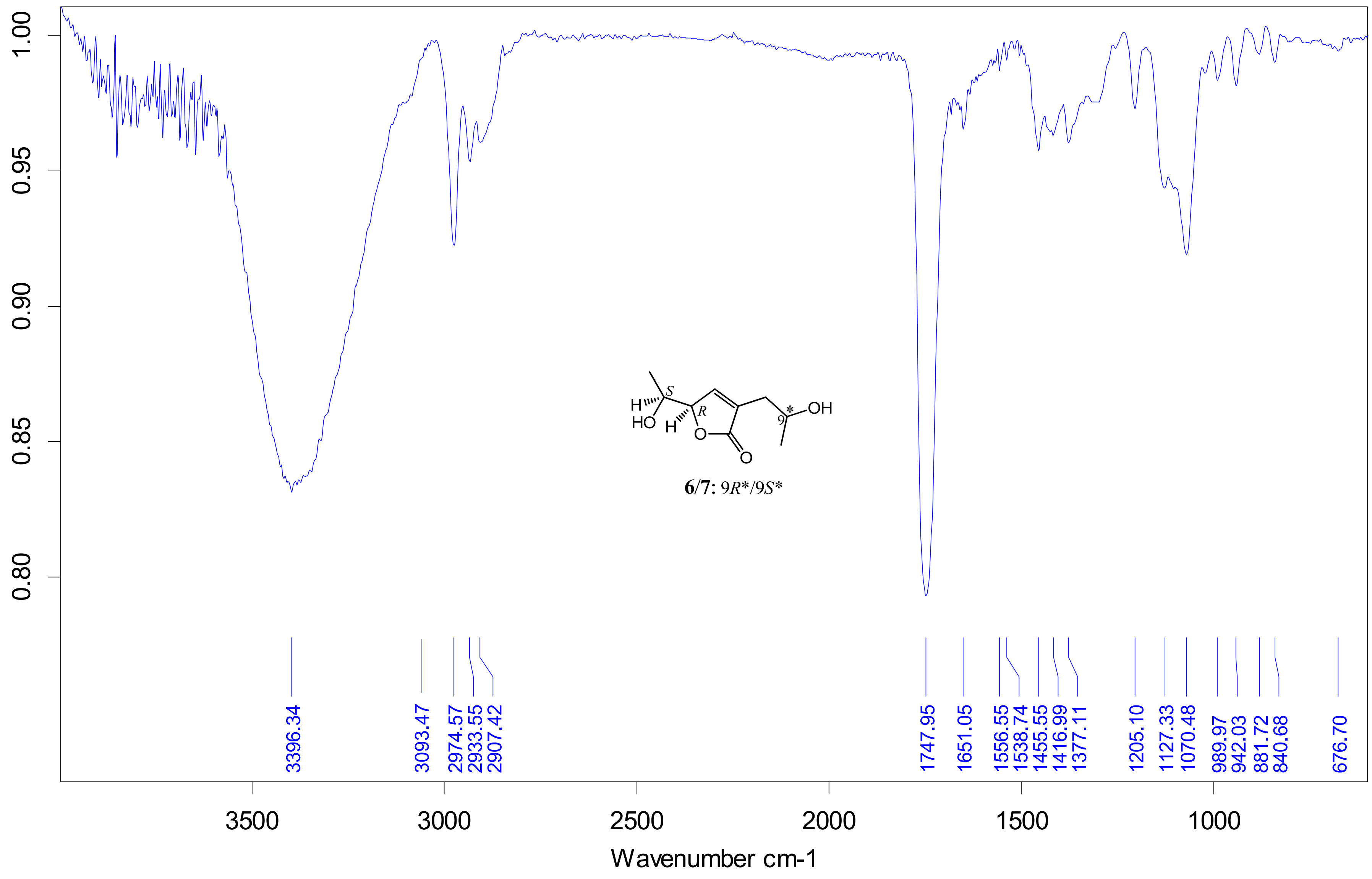

Figure S47. 400 MHz ^{1}H NMR spectrum of **6**/**7** in CD_3OD.

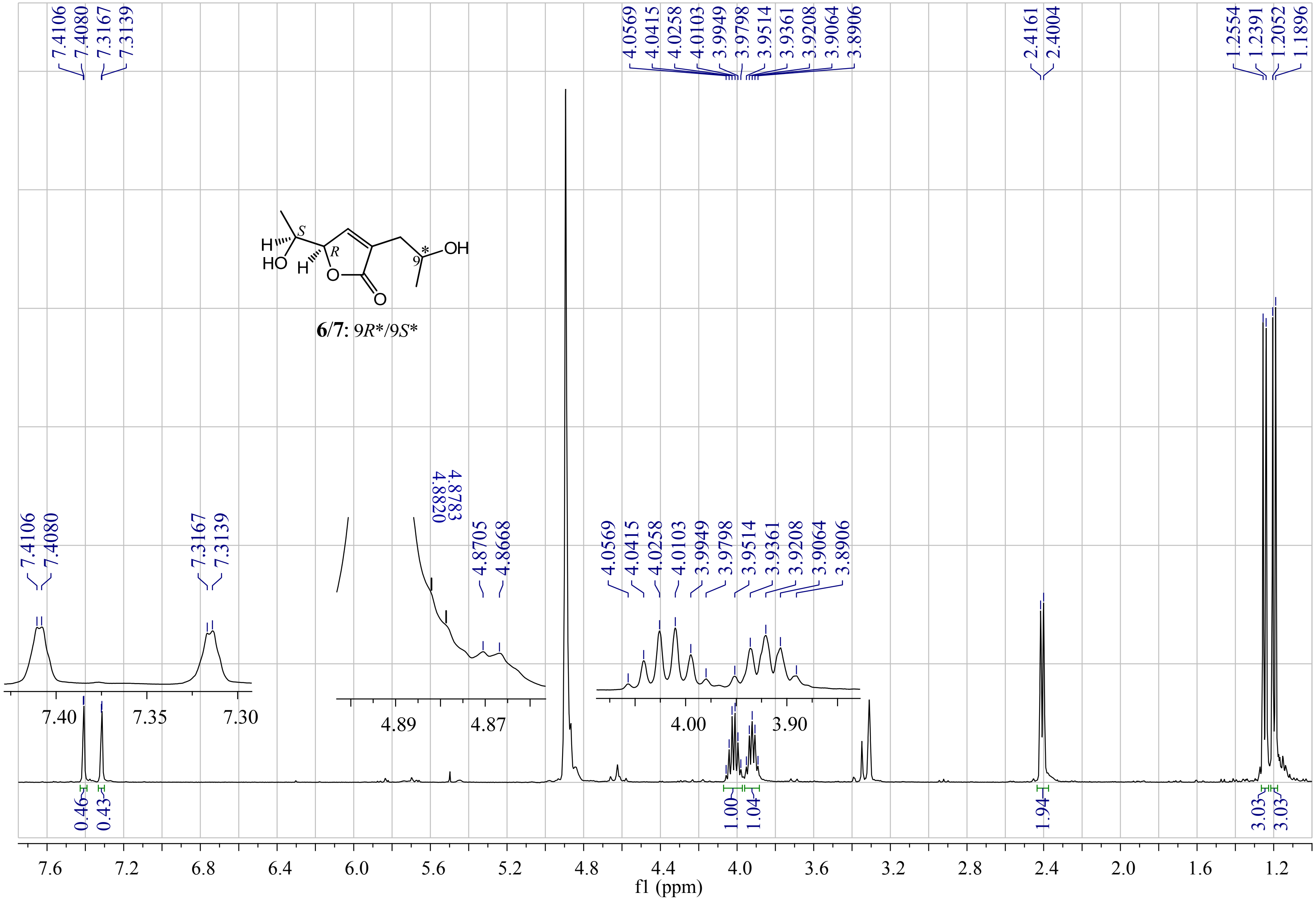

Figure S48. 100 MHz ^{13}C NMR spectrum of **6**/**7** in CD_3OD.

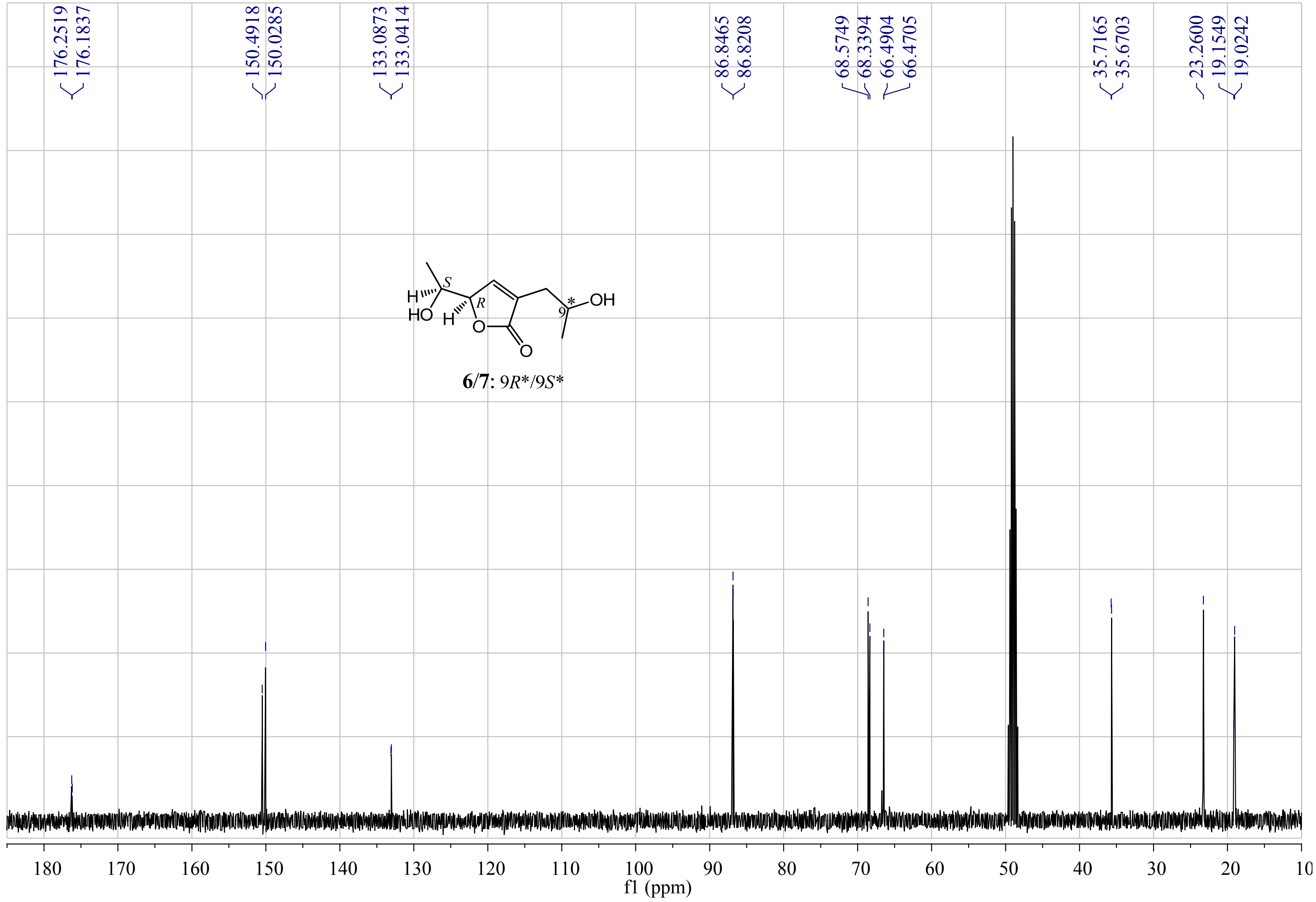

Figure S49. HMQC spectrum of **6**/**7** in CD_3OD.

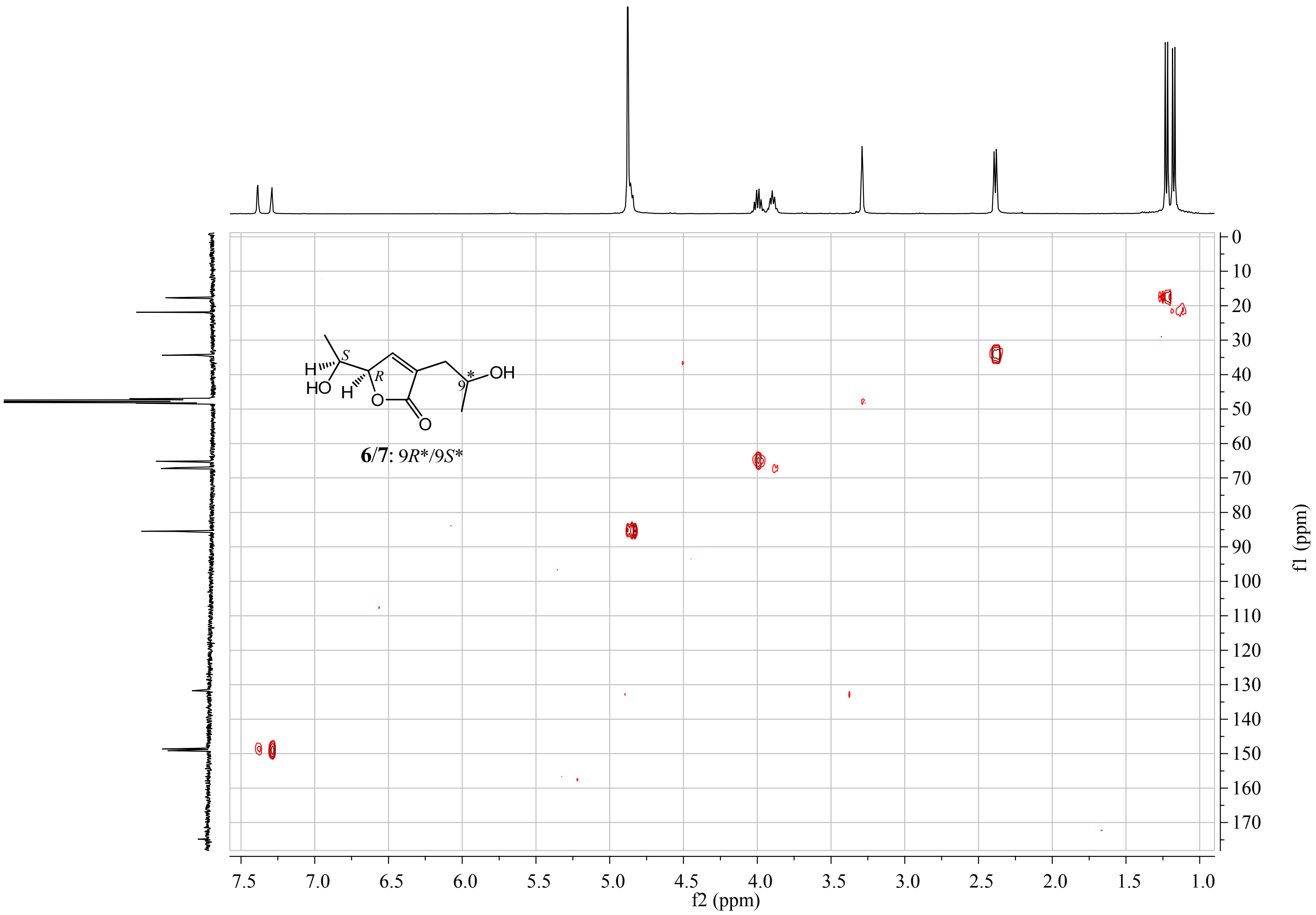

Figure S50. ^{1}H–^{1}H COSY spectrum of **6**/**7** in CD_3OD.

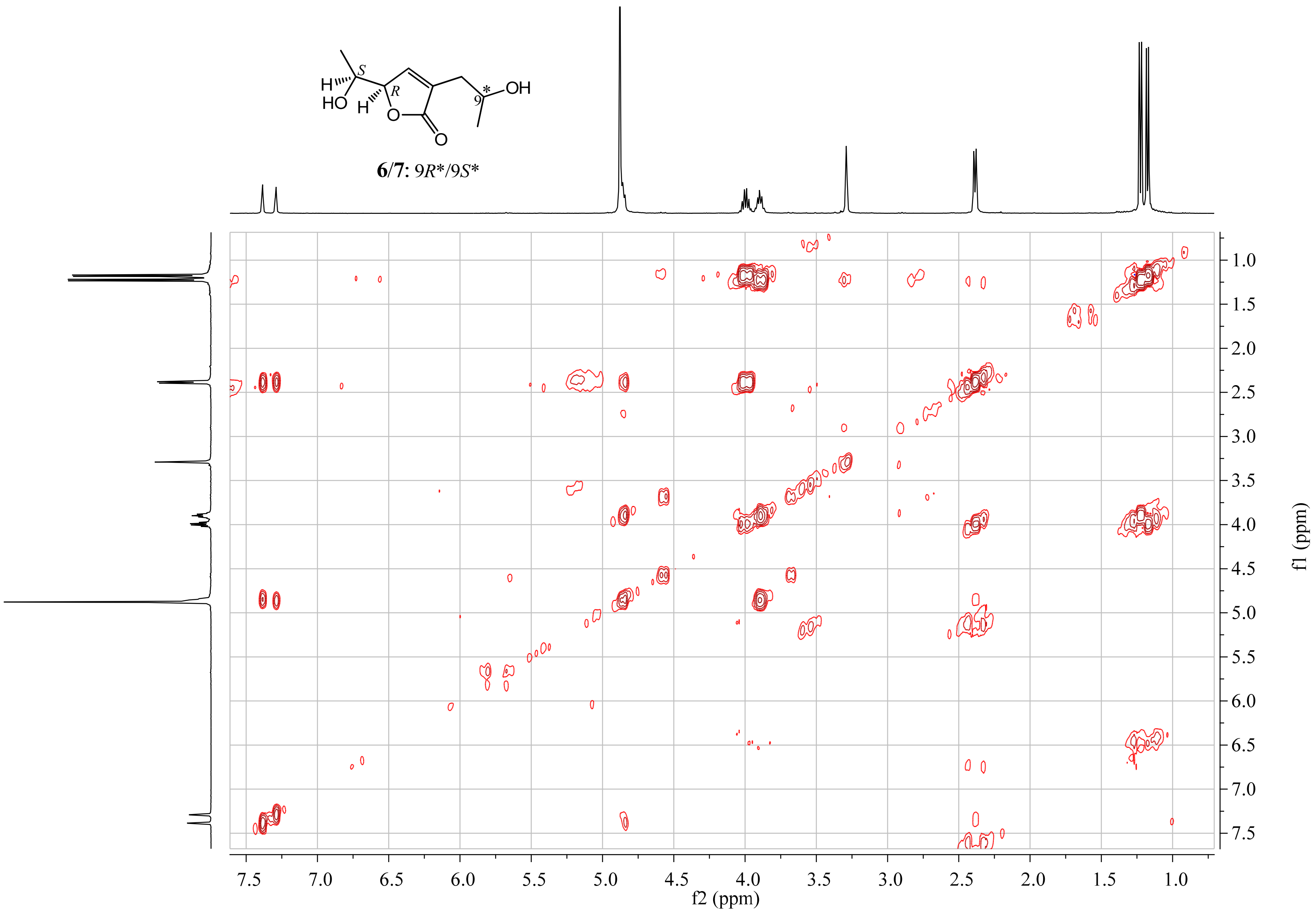

Figure S51. HMBC spectrum of **6**/**7** in CD_3OD.

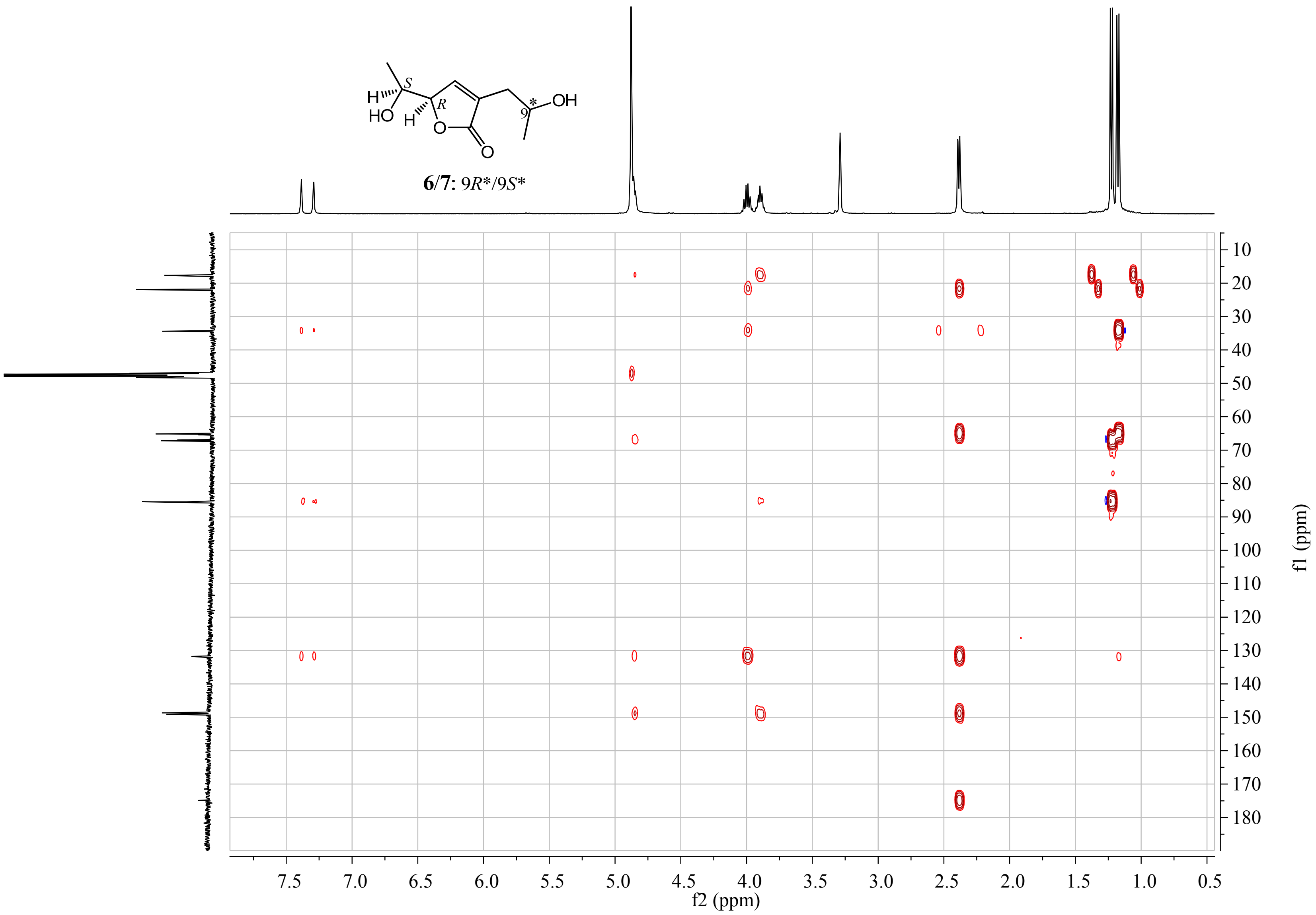

Figure S52. Positive ion ESI-MS spectrum of **9**.

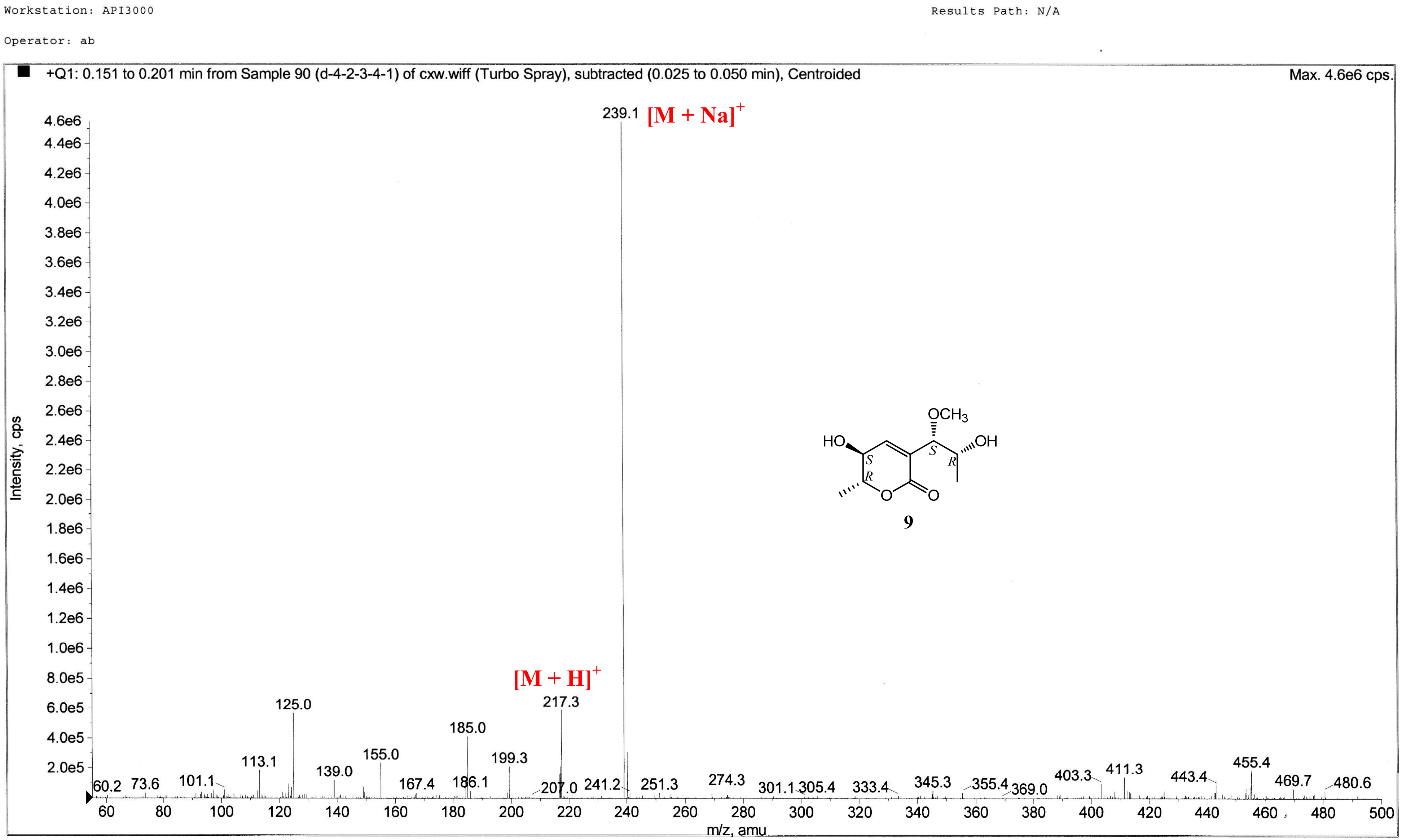

Figure S53. Positive ion HR-ESI-MS spectrum of **9**.

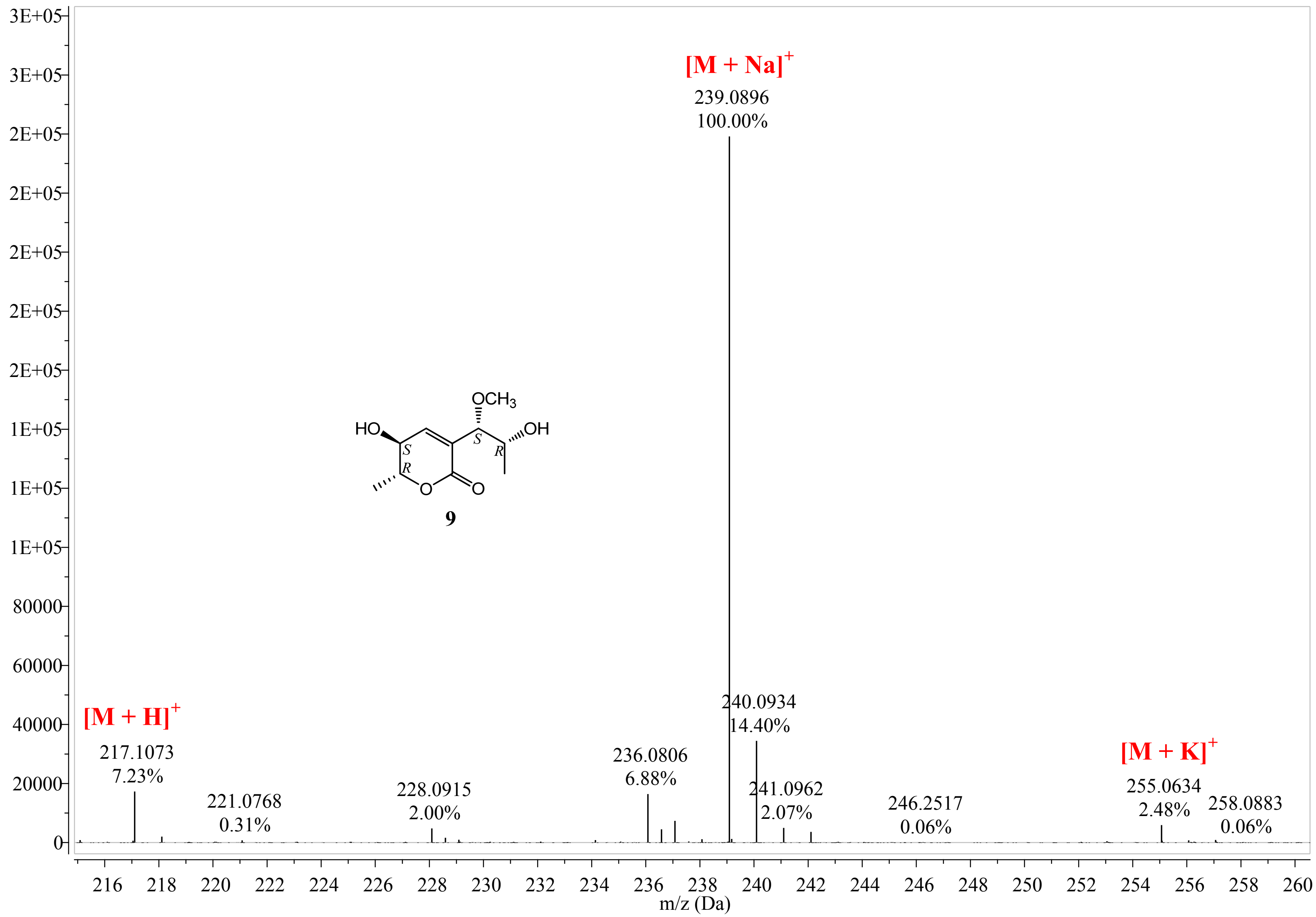

Figure S54. IR spectrum of **9**.

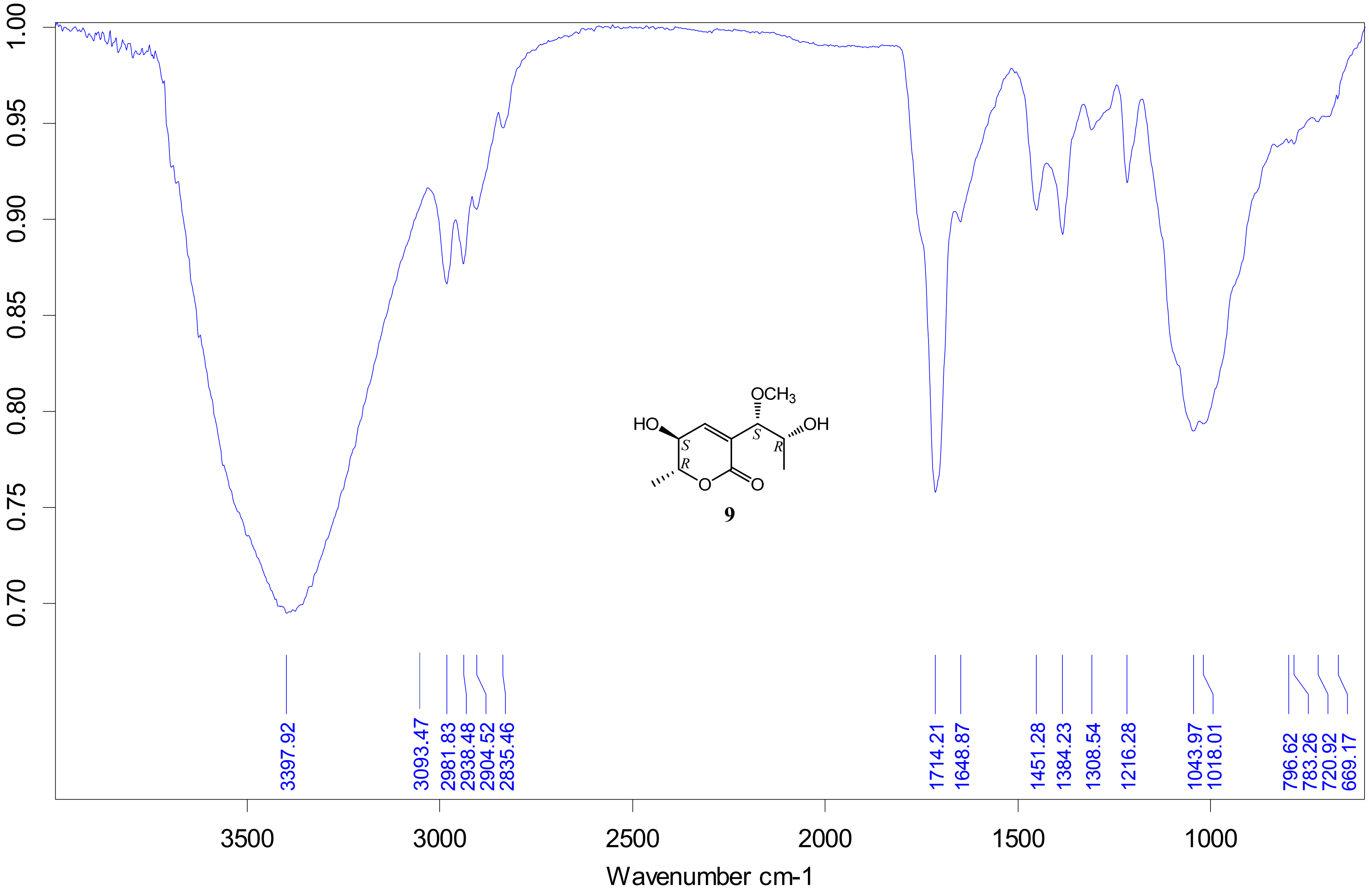

Figure S55. 400 MHz ^{1}H NMR spectrum of **9** in CD_3OD.

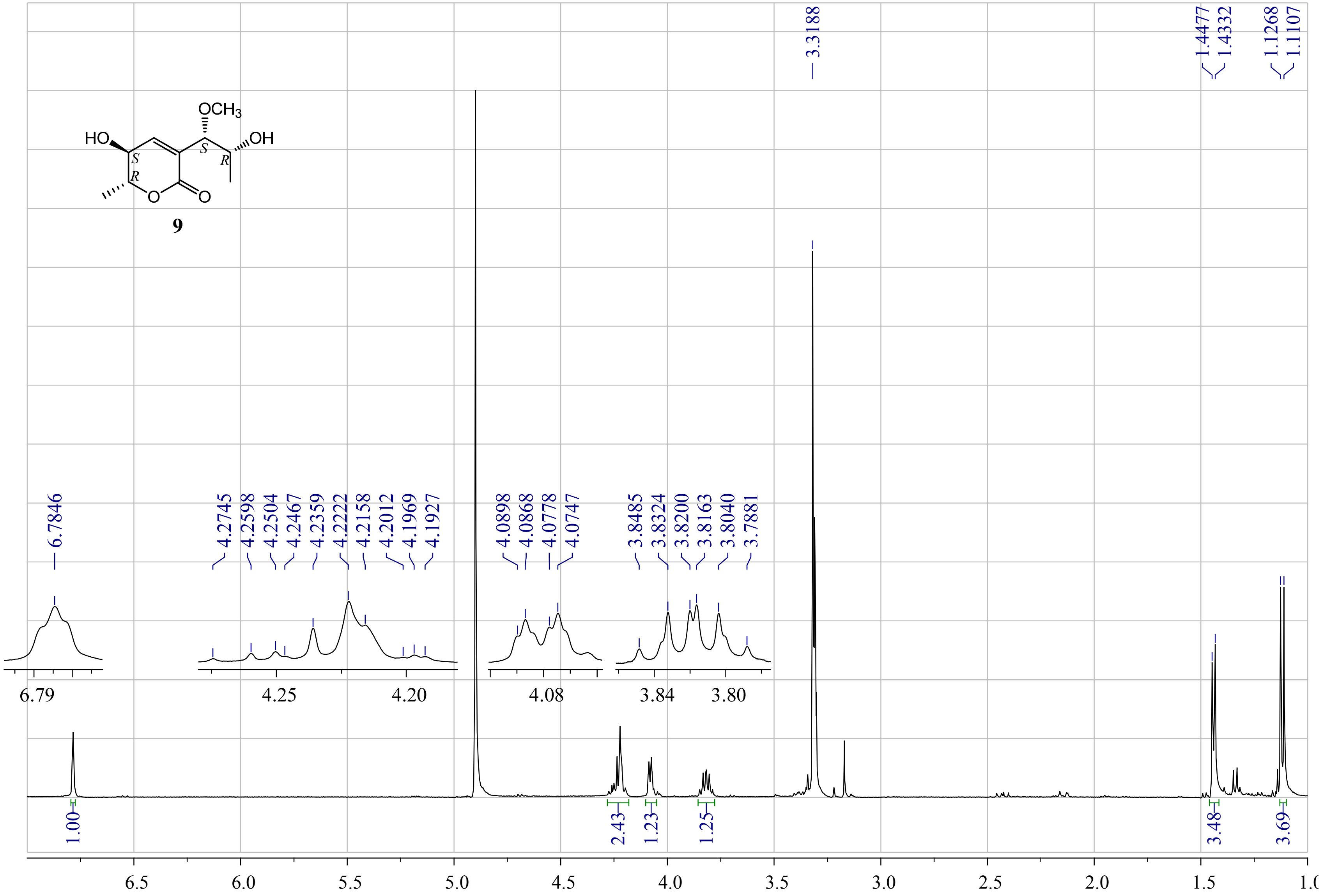

Figure S56. 100 MHz ^{13}C NMR spectrum of **9** in CD_3OD.

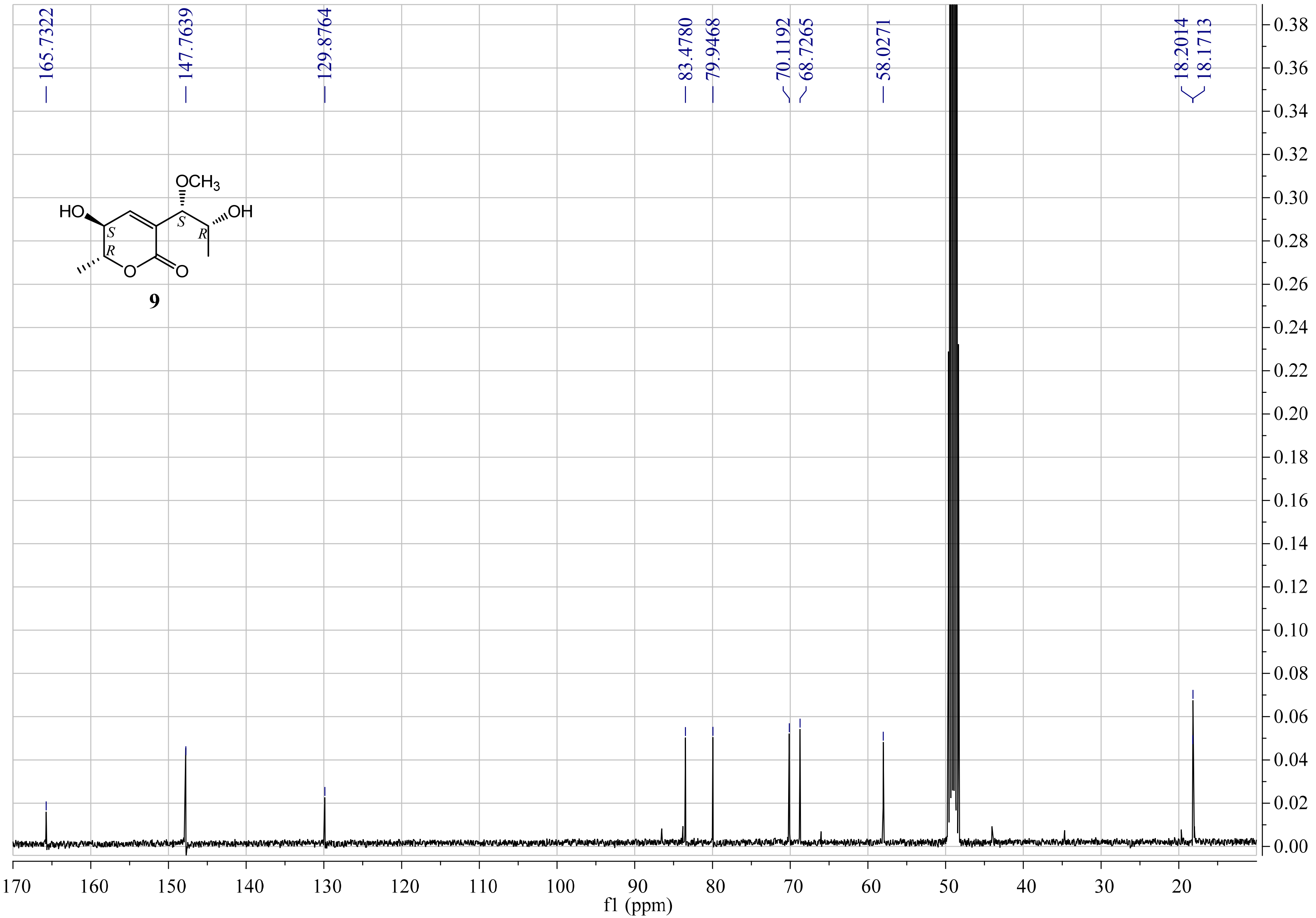

Figure S57. HMQC spectrum of **9** in CD_3OD.

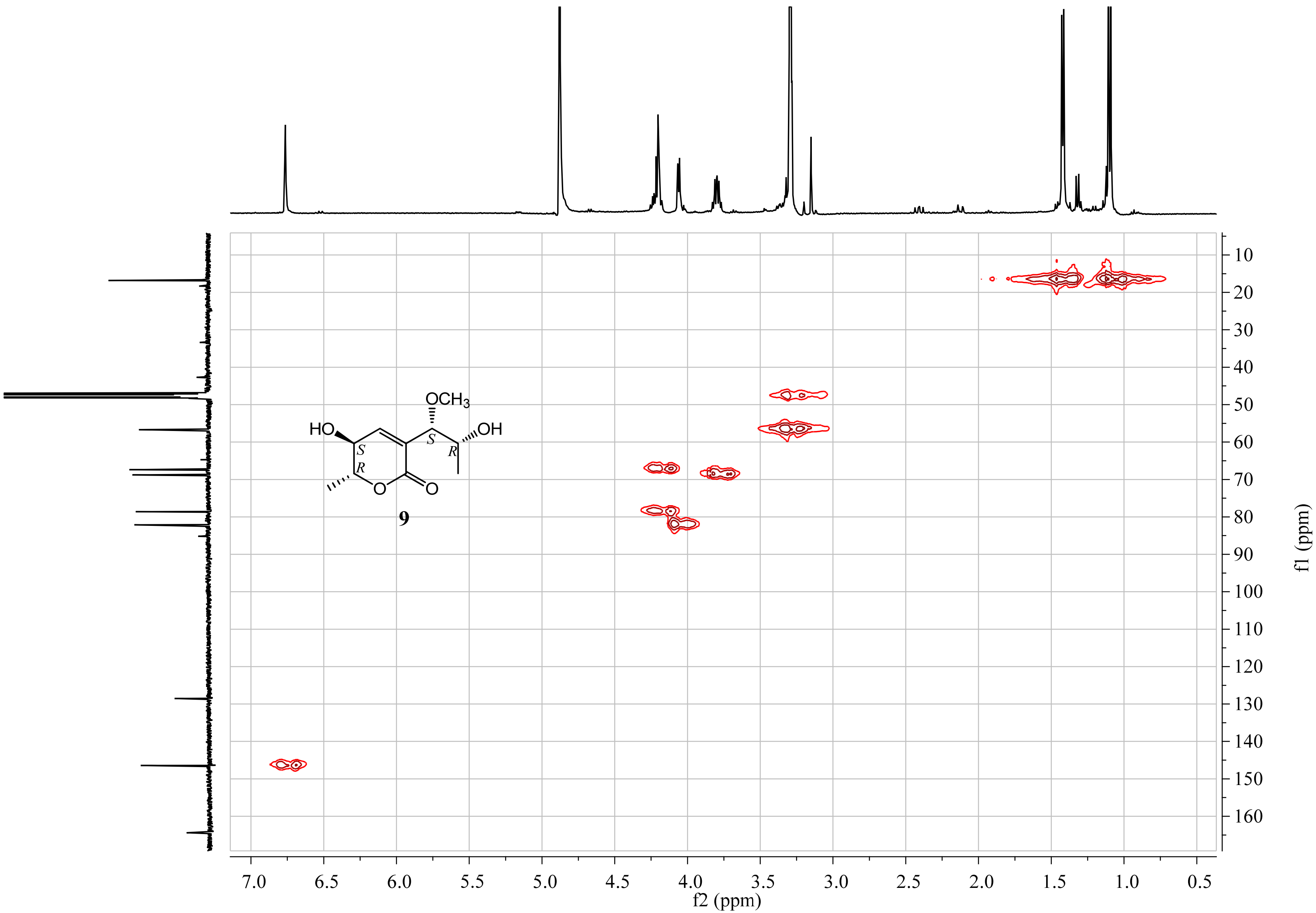

Figure S58. ^{1}H–^{1}H COSY spectrum of **9** in CD_3OD.

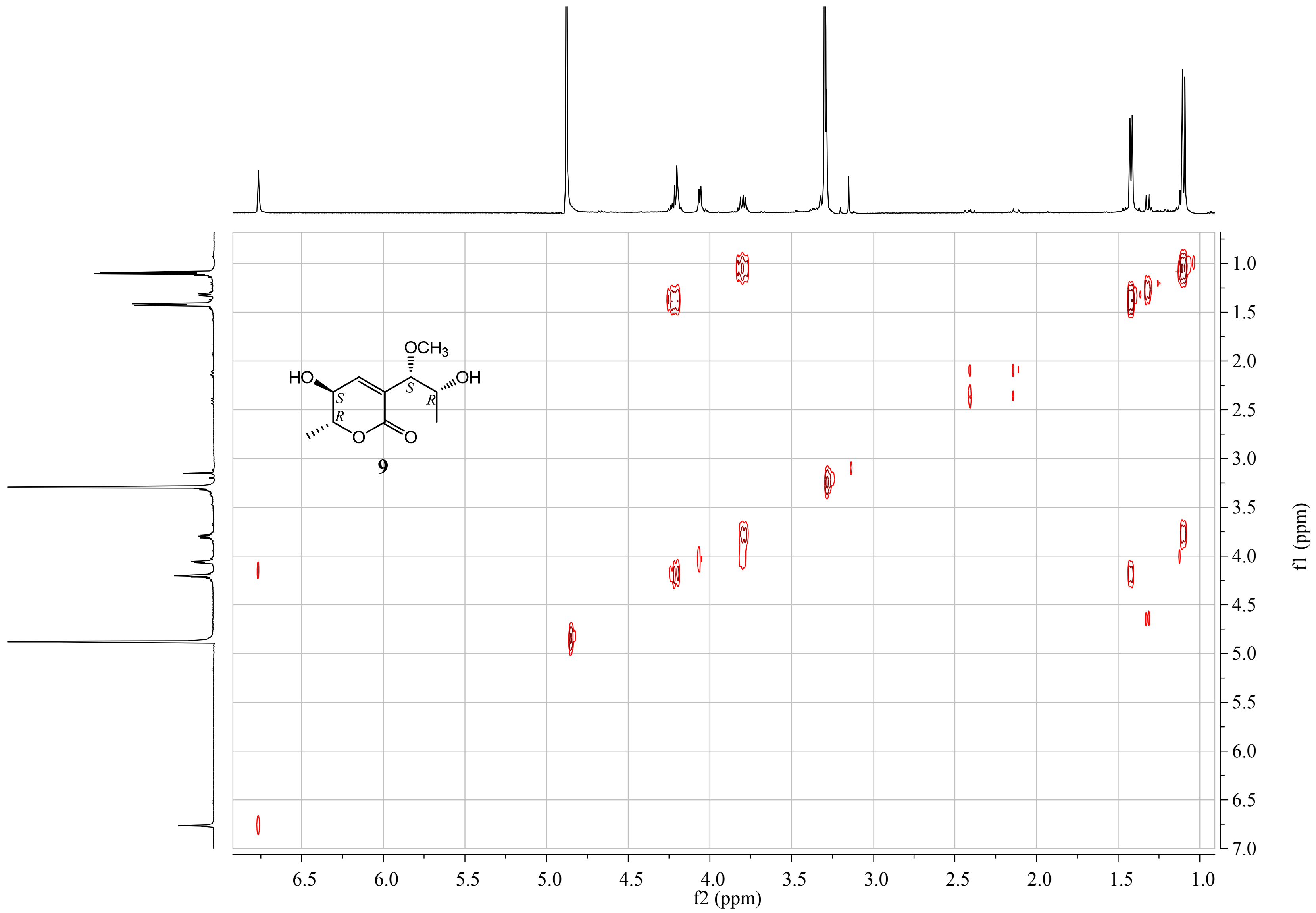

Figure S59. HMBC spectrum of **9** in CD_3OD.

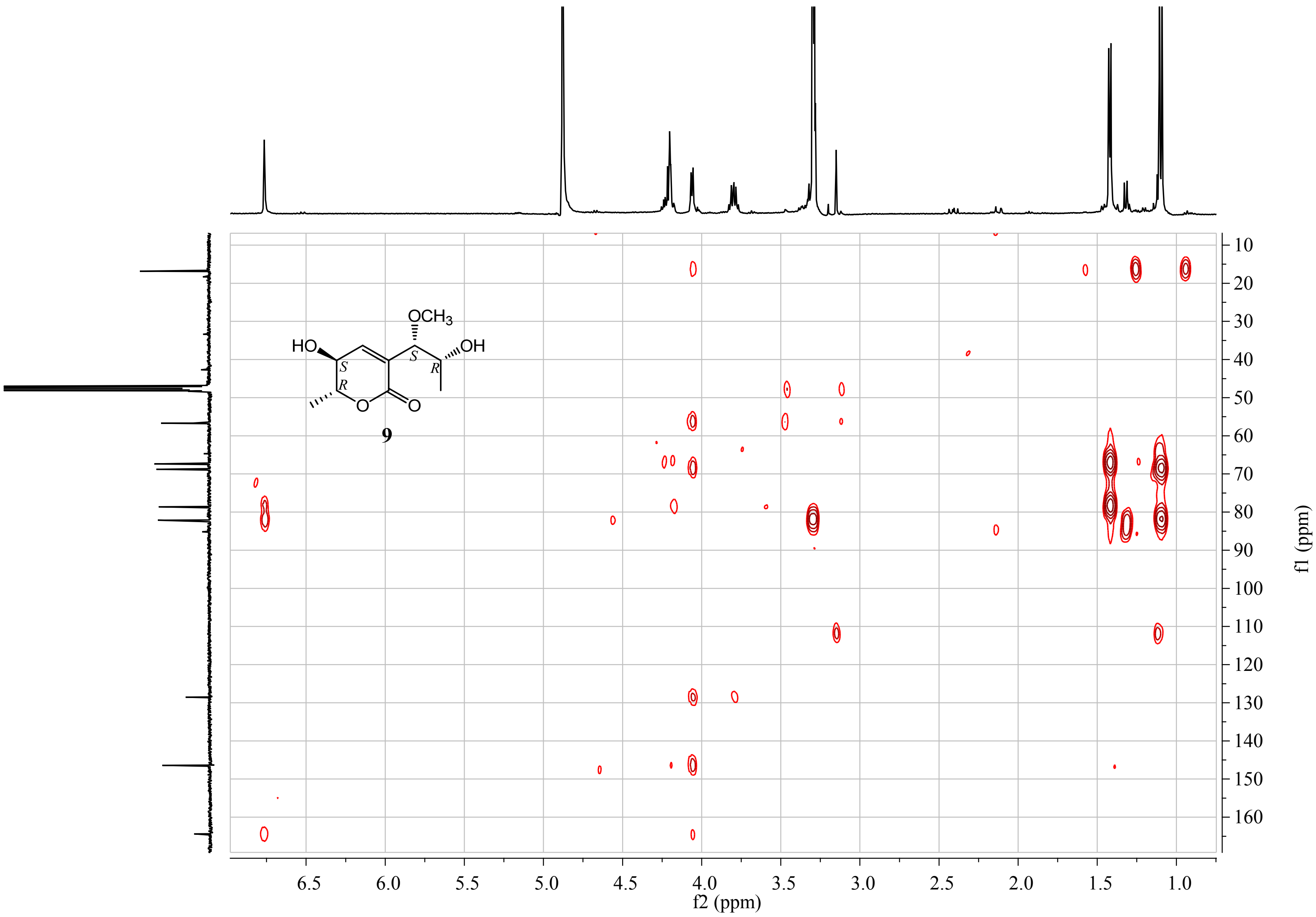

Figure S60. 400 Hz ^{1}H NMR spectrum of **9** in $CDCl_3$.

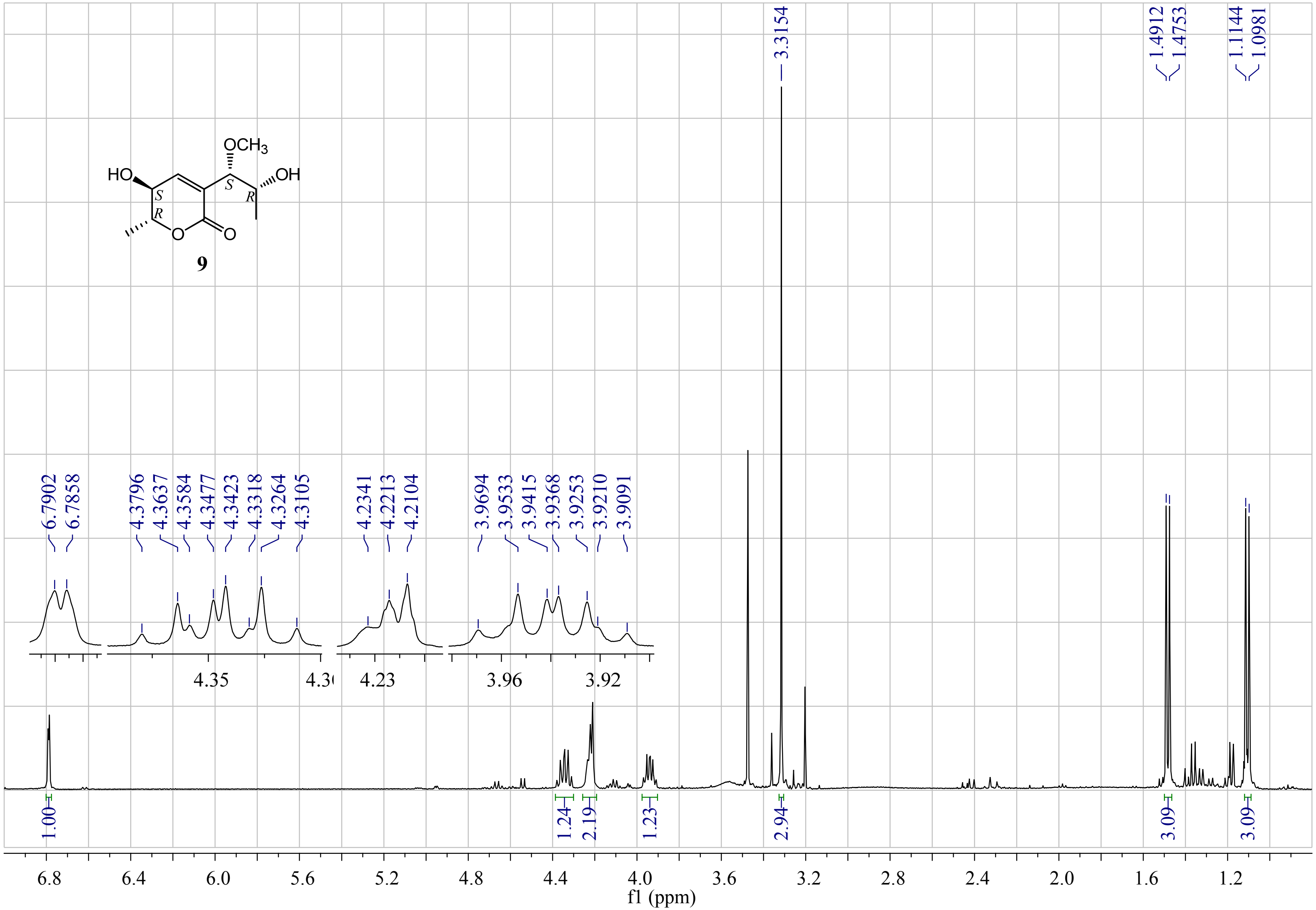

Figure S61. ^{1}H–^{1}H COSY spectrum of **9** in $CDCl_3$.

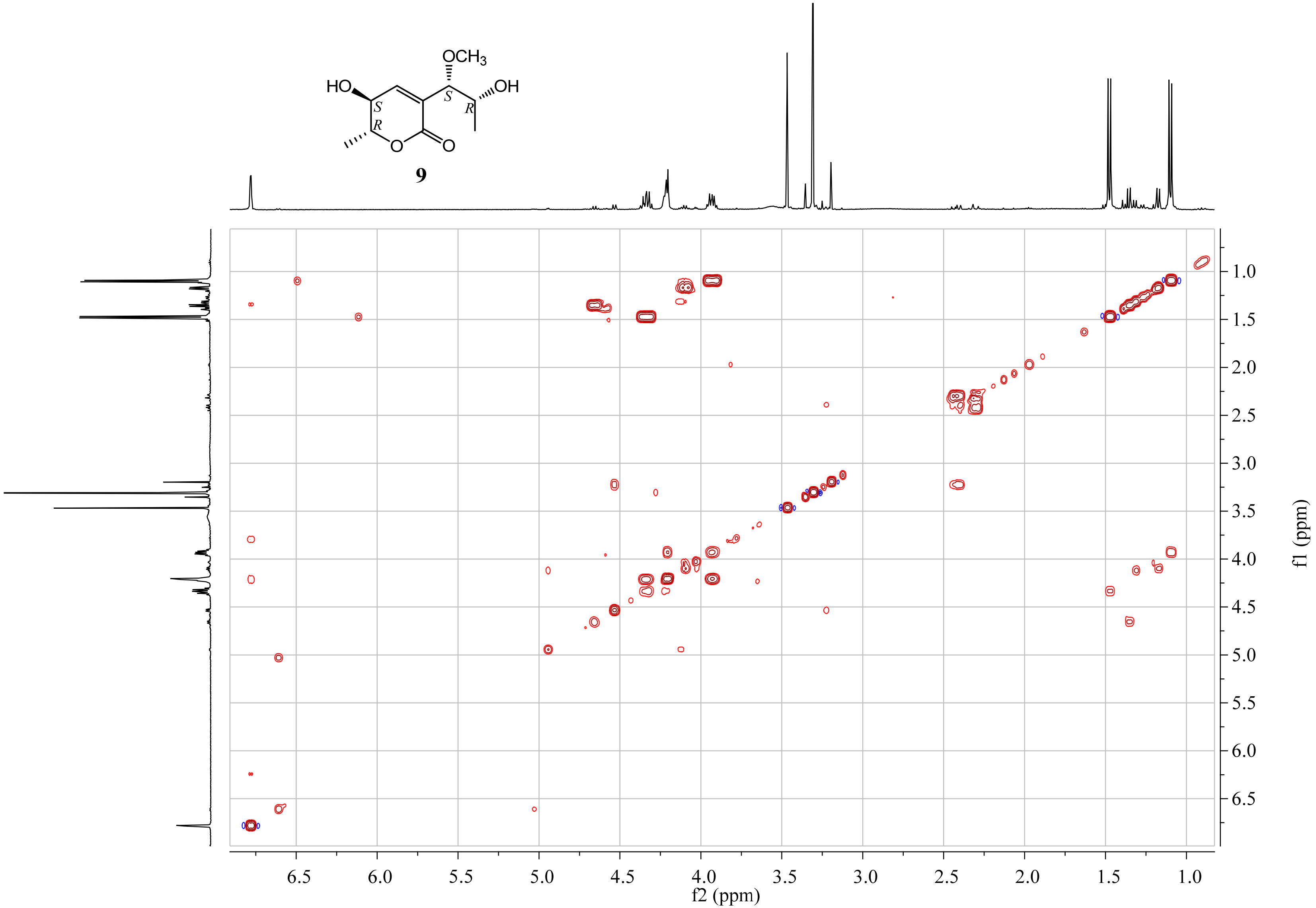

Figure S62. Difference NOE spectrum of **9** in $CDCl_3$.

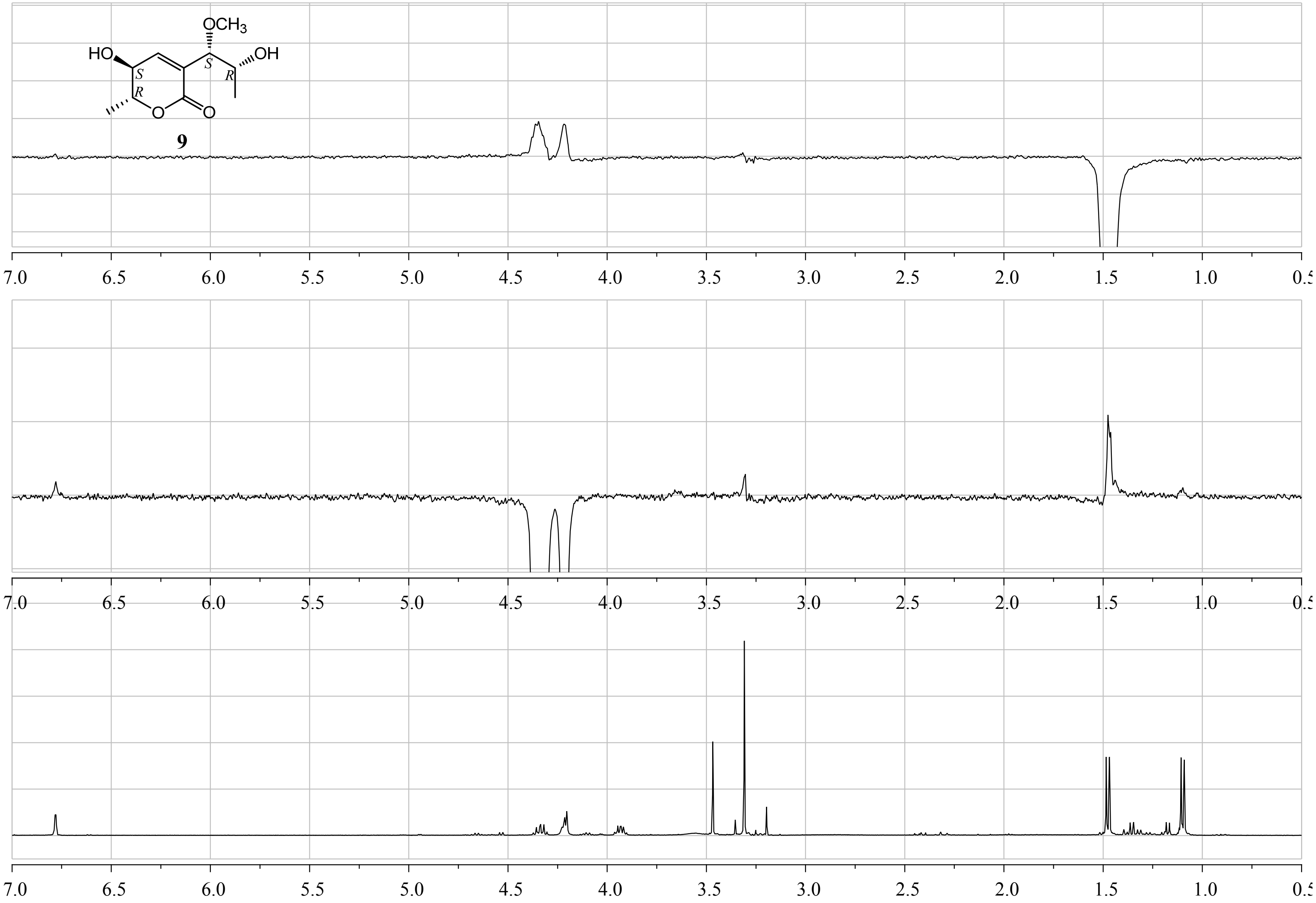

Figure S63. 400 M Hz ^{1}H NMR spectrum of the (*S*)- and (*R*)-MTPA esters of **9** in pyridine-d_6.

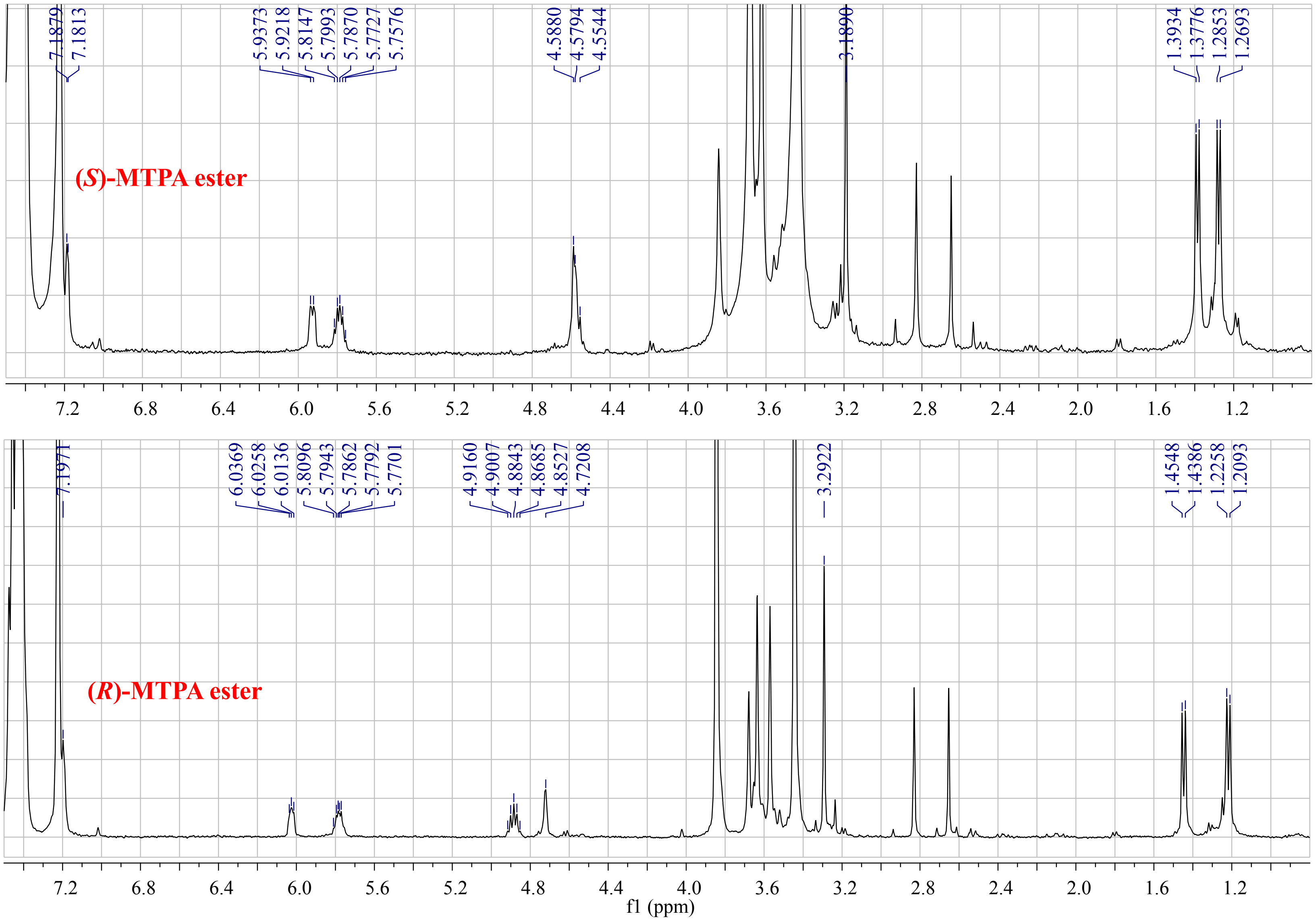

Figure S64. Positive ion ESI-MS spectrum of **11**.

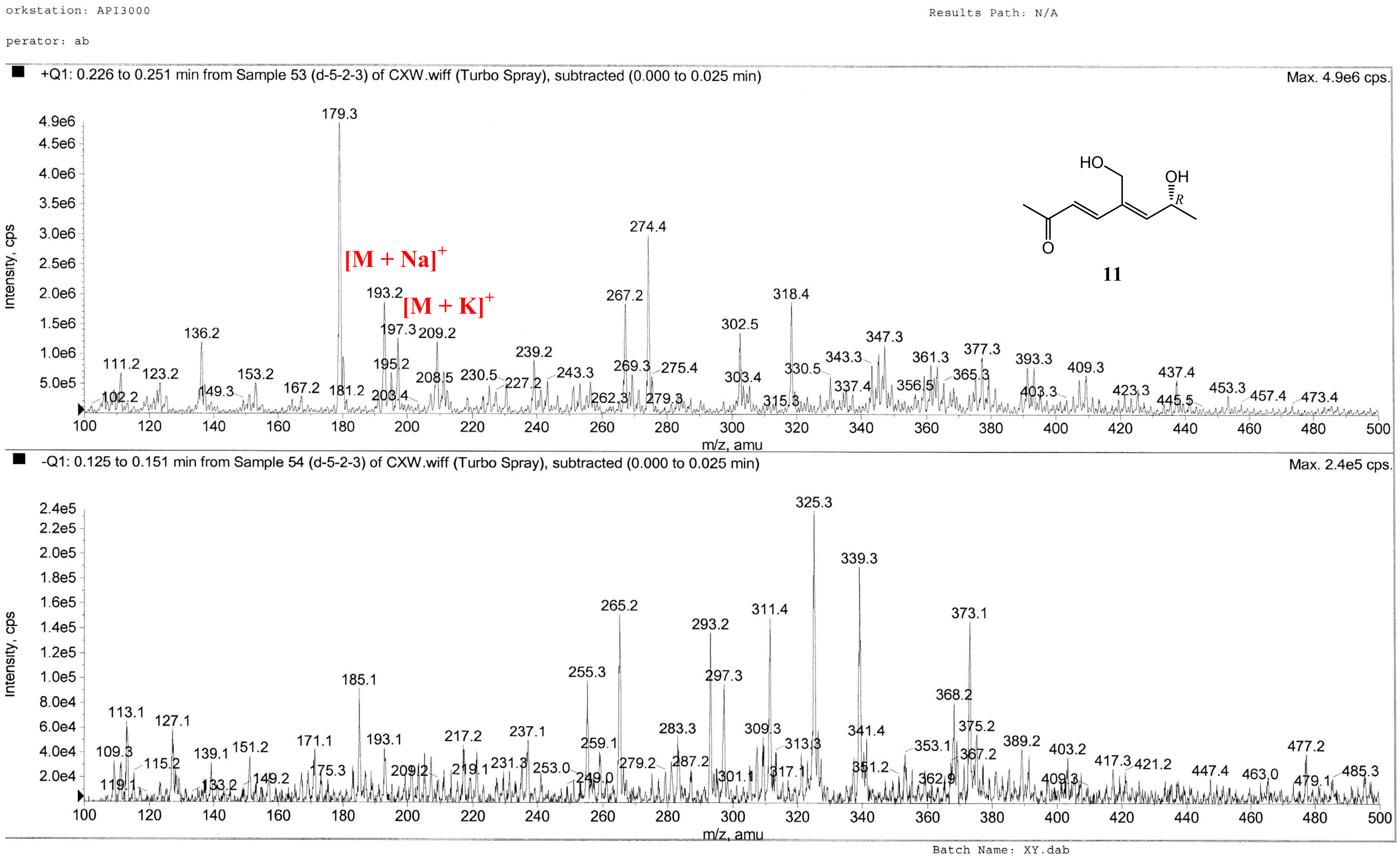

Figure S65. Positive ion HR-ESI-MS spectrum of **11**.

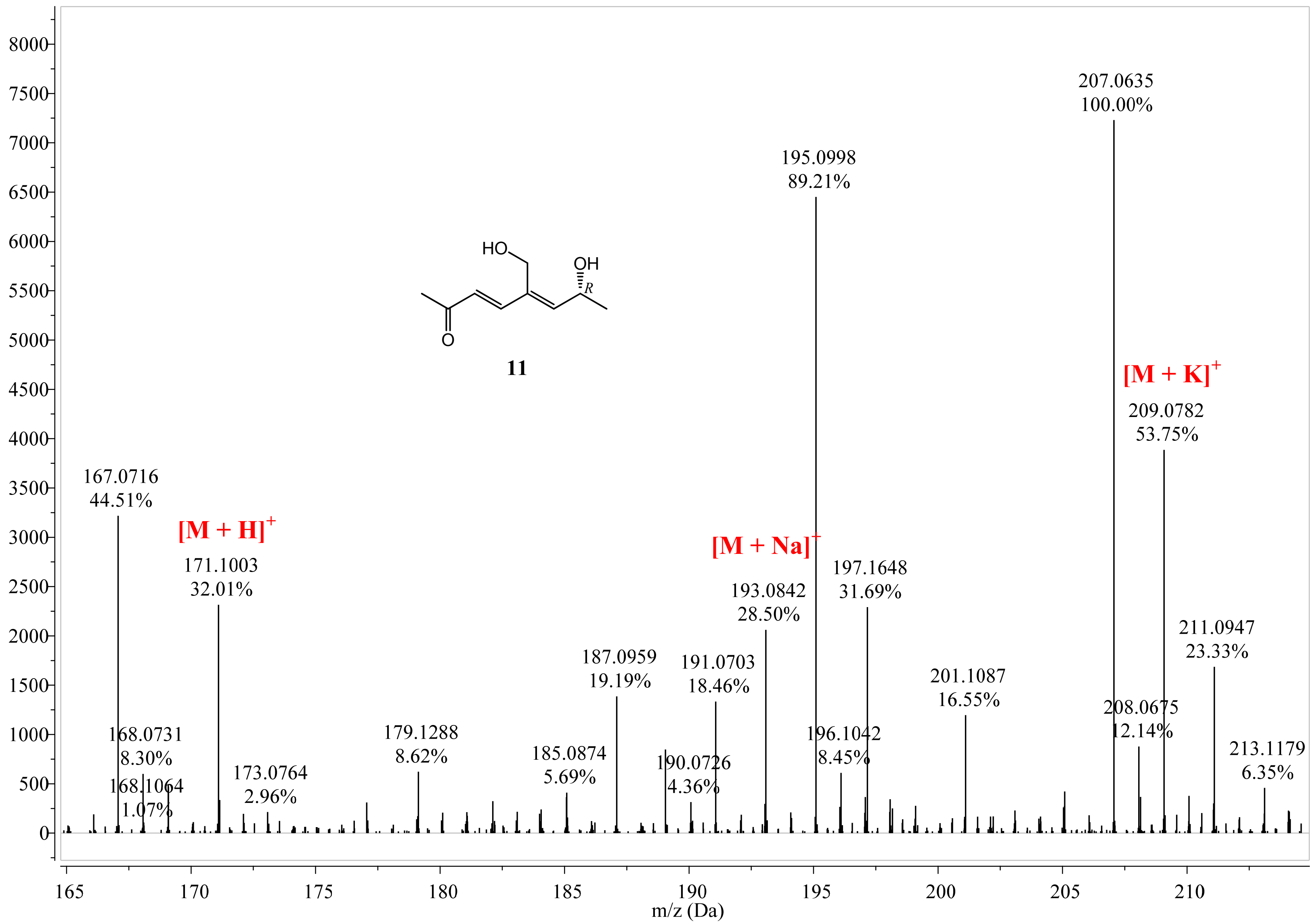

Figure S66. 400 Hz ^{1}H NMR spectrum of **11** in CD_3OD.

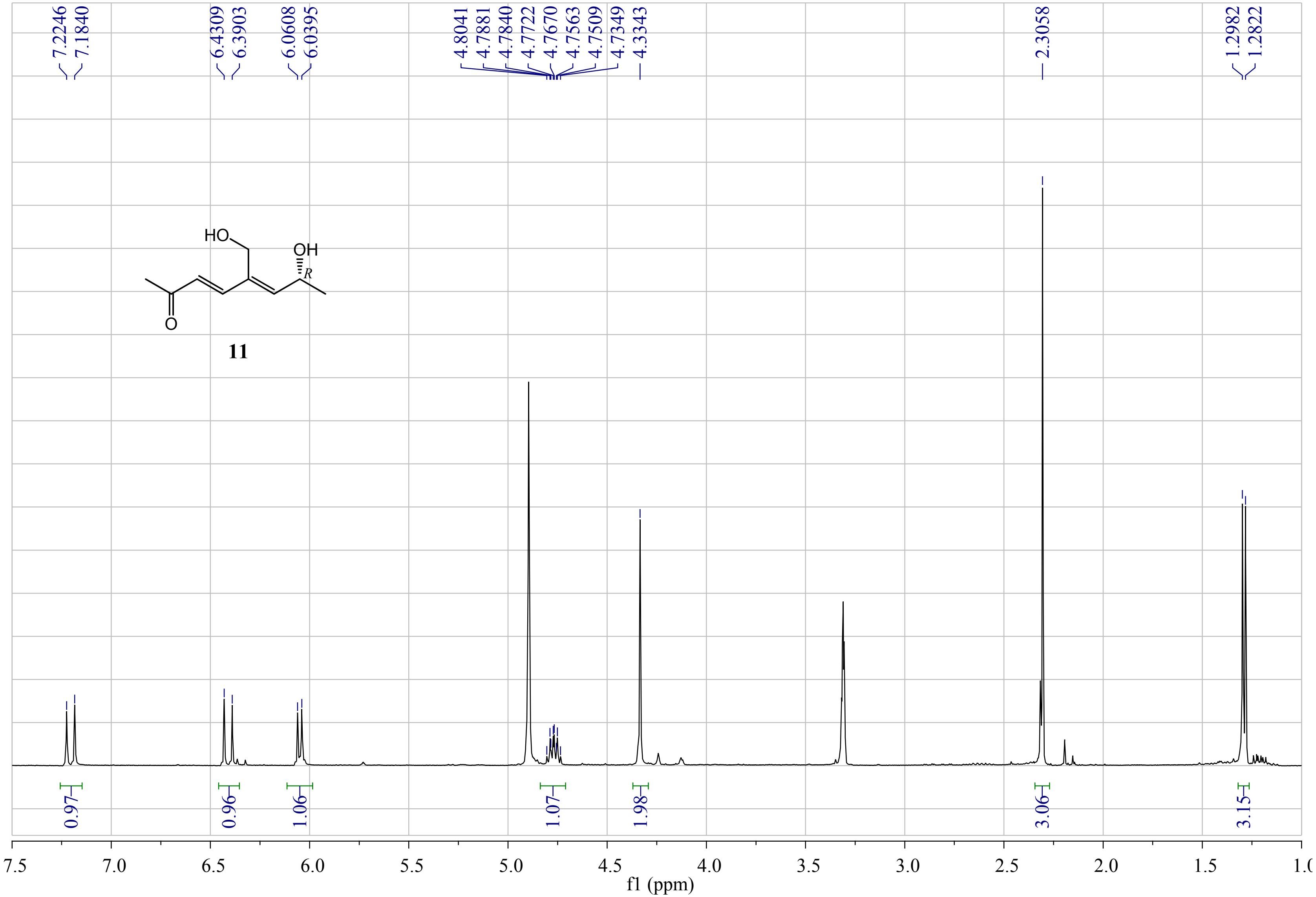

Figure S67. 100 Hz ^{13}C NMR spectrum of **11** in CD_3OD.

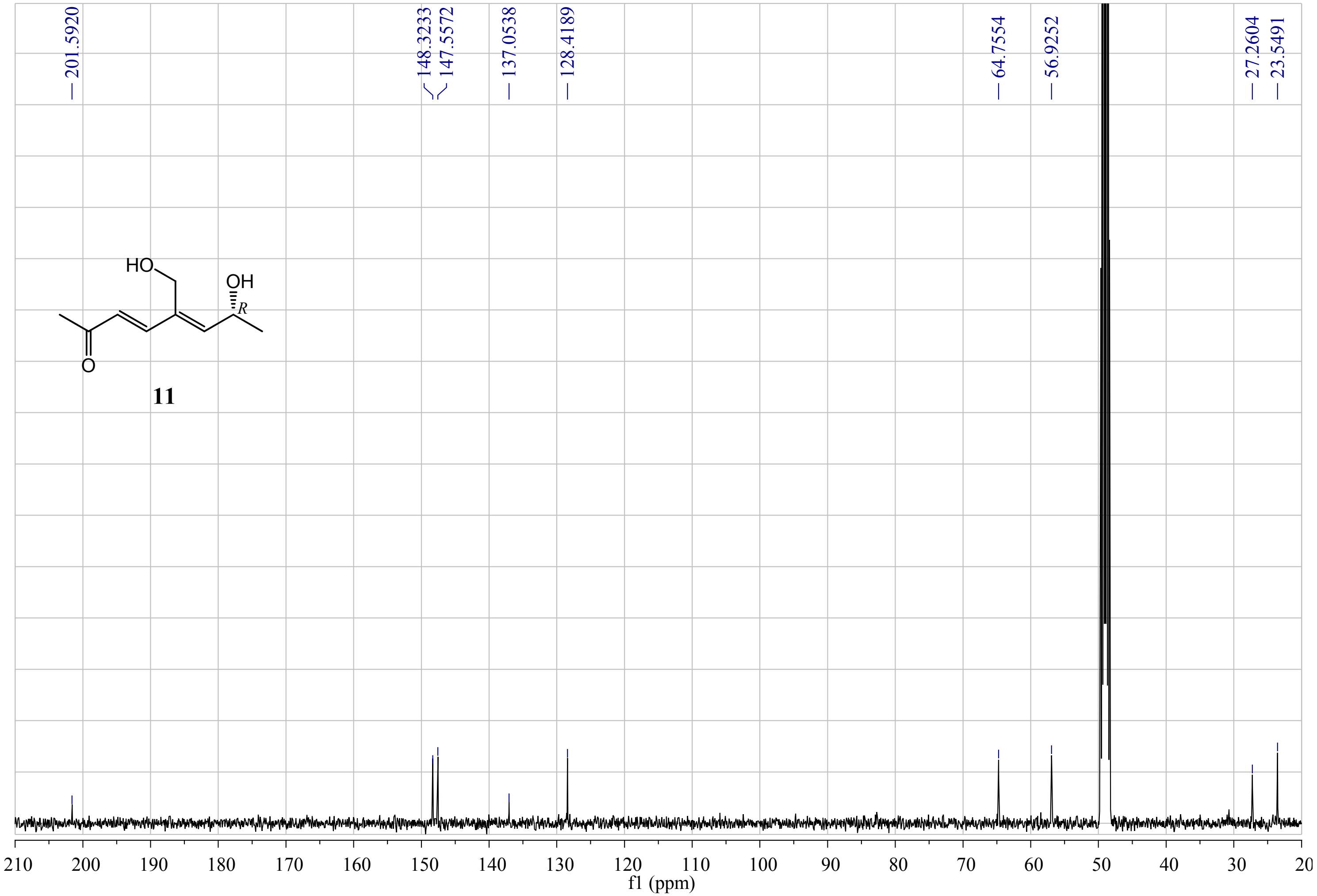

© 2014 by the authors; licensee MDPI, Basel, Switzerland. This article is an open access article distributed under the terms and conditions of the Creative Commons Attribution license (http://creativecommons.org/licenses/by/3.0/).
